# Synthesis and Antifungal Activity of 1,2,4-Oxadiazole Derivatives

**DOI:** 10.3390/molecules30081851

**Published:** 2025-04-20

**Authors:** Lili Yu, Kuan Yang, Lin Yao, Nana Wang, Hui Kang, Guangda Yao, Xiaomeng Li, Bei Qin

**Affiliations:** 1 Xi’an Key Laboratory of Multi Synergistic Antihypertensive Innovative Drug Development, Xi’an Medical University, Xi’an 710021, China; yulili@xiyi.edu.cn (L.Y.); yangkuan@xiyi.edu.cn (K.Y.); wangnana@xiyi.edu.cn (N.W.); 2 Xi’an Innovative Antihypertensive Drugs International Science and Technology Cooperation Base, Xi’an 710021, China; 3 Institute of Drug Research, Xi’an Medical University, Xi’an 710021, China; 4 College of Pharmacy, Xi’an Medical University, Xi’an 710021, Chinayaogd2022@shanghaitech.edu.cn (G.Y.);

**Keywords:** antifungal activity, 1,2,4-oxadiazole derivatives, biological evaluation, molecular docking

## Abstract

1,2,4-Oxadiazole derivatives containing anisic acid or cinnamic acid were designed and synthesized, which were expected to be an effective Succinate dehydrogenase (SDH) inhibitor, and their structures were characterized by ^1^H NMR, ^13^C NMR, and ESI-MS. The antifungal activity of the compounds against plant pathogenic fungi was screened by the mycelial growth inhibition test in vitro. Compounds **4f** and **4q** showed significant antifungal activities against *Rhizoctonia solani (R. solani)*, *Fusarium graminearum (F. graminearum)*, *Exserohilum turcicum (E. turcicum), Botrytis cinerea (B. cinerea)*, and *Colletotrichum capsica (C. capsica)*. The *EC_5_*_0_ values of **4q** were 38.88 μg/mL, 149.26 μg/mL, 228.99 μg/mL, and 41.67 μg/mL against *R. solani*, *F. graminearum*, *E. turcicum*, and *C. capsica*, respectively, and the *EC*_50_ values of **4f** were 12.68 μg/mL, 29.97 μg/mL, 29.14 μg/mL, and 8.81 μg/mL, respectively. Compound **4f** was better than commercial carbendazim against *Exserohilum turcicum*. Compounds **4f** and **4q** showed an antifungal effect on *C. capsica* of capsicum in vivo. Molecular docking simulation showed that **4f** and **4q** interacted with the target protein through the hydrogen bond and hydrophobic interaction, in which **4q** can form hydrogen bonds with TRP173 and ILE27 of SDH, and **4f** had hydrogen bonds with TYR58, TRP173, and SER39. This also explains the possible mechanism of action between the inhibitor and target protein.

## 1. Introduction

According to the United Nations Department of Economic and Social Affairs, the global population will reach 9.7 billion by 2050 [1], challenging global food production. Worldwide, pathogens and pests can reduce crop yields by 20–40% [2,3], and the losses caused by pathogens can reach 8% to 21% [4]. Therefore, it is urgent to develop new antimicrobial agents with high efficiency, broad spectrum, and environmental protection. Succinate dehydrogenase inhibitor (SDHI) fungicides are widely used against various pathogens, such as F. graminearum [5], leaf blight [6], rust [7,8], and gray mold [9]. Since the launch of the first succinate dehydrogenase inhibitor fungicide carboxin in 1966 [10], more than 20 kinds of such fungicides have entered the market so far. Among them, boscalid, developed by BASF in 2003, has become one of the most widely used (SDHI) fungicides [11]. Boscalid led the development of SDHI fungicides. Subsequently, Fluxapyroxad, Benzovindiflupyr, Penflufen, Sedaxane [12], and other fungicides with high market prospects were introduced. Succinate dehydrogenase (SDH) is a functional component of the tricarboxylic acid cycle and aerobic respiration, plays a key role in fungal metabolism, and is an important target for antifungal research [13,14]. SDHI fungicides can inhibit or kill pathogens by affecting the respiratory chain electron transport system of pathogens and blocking their energy metabolism, thus achieving the purpose of controlling diseases caused by fungi [15,16]. Therefore, in 2009, the Action Committee on Antimicrobial Resistance (FRAC) classified fungicides such as carboxamide as SDH inhibitors (SDHIs), which typically contain aromatic rings, heterocycles, or amides [17,18].

Oxadiazole research has a history of more than 100 years, covering symmetric 1,3,4-oxadiazoles and asymmetric 1,2,4-oxadiazoles [19]. Extensive studies have revealed that oxadiazole exhibits diverse biological activities, such as antioxidant [20,21], anti-inflammatory [22,23], antibacterial [19,24,25], anti-tuberculosis [26,27], and anti-tumor activities [28,29,30]. Literature survey indicated that azole heterocyclic rings containing multiple nitrogen atoms show significant antifungal activity in vivo and in vitro [31]. Some studies also show that oxadiazole has good antibacterial [24] and antifungal activities [32,33,34].

Analysis of currently marketed SDHIs reveals that heterocycles, particularly pyrazole rings, are prevalent structures in SDHIs [35], like sedaxane (Syngenta), fluxapyroxad (BASF), bixafen (Bayer), pydiflumetofen (Syngenta), etc. Moreover, SDHI fungicides often contain a pyridine ring or an amide structure. Notably, to prevent fungal resistance to the pyrazole–ring parent nucleus, researchers are focusing on introducing other heteroatoms, such as oxygen or sulfur, into the heterocyclic structure.

**Scheme 1:**
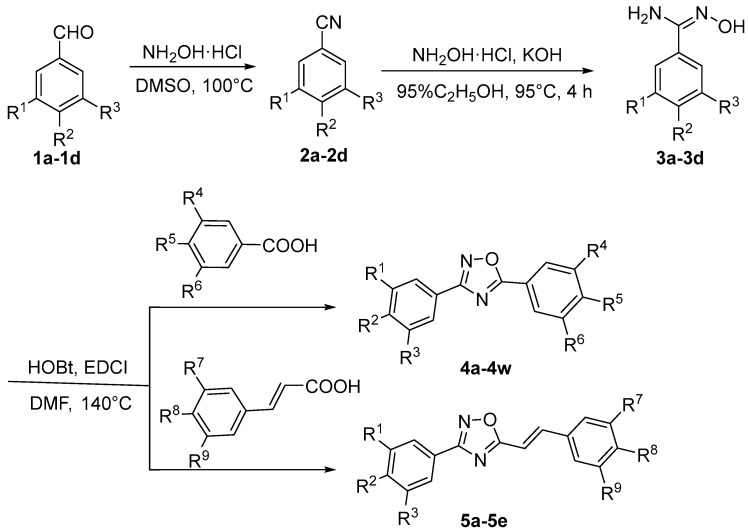
Synthetic routes and chemical structures of the target compounds.

In 2016, Li Shengkun et al. reported a series of oxadiazole derivatives with good inhibitory effects on *R. solani*, *Botrytis Cinerea*, *Sclerotinia Sclerotiorum*, and *R. solani* [36]. Wang et al. designed and synthesized furan-based SDHI fungicides effective against *R. solani* [37]. Given this, the oxygen-containing oxadiazole ring shows potential for fungicide development. Thus, introducing 1,2,4-oxadiazole into SDHI design to verify its potential as antifungal molecules is an interesting research direction. Notably, reported SDHI antifungal agents all contain at least one benzene ring, suggesting its essential role in antifungal activity. As shown in Scheme 1, based on representative SDHIs’ structural features, we designed a series of 1,2,4-oxadiazole antifungals. Their key structural feature is two benzene rings linked by an oxadiazole ring, with substituents like phenolic hydroxyl, halogen, nitro, and methoxy on the benzene rings. In this study, 27 1,2,4-oxadiazole derivatives with anisic acid or cinnamic acid were designed, synthesized, and characterized by ^1^H NMR, ^13^C NMR, and ESI-MS. Their antifungal activities against *R. solani*, *F. graminearum*, *E. turcicum*, *B. cinerea*, and *C. capsica* were evaluated via in vitro tests. The impact of the most active compounds on the E. turcicum hyphal state was assessed. Finally, molecular docking simulations explored the possible binding modes between SDH and the inhibitors.

## 2. Results

### 2.1. Synthesis of 1,2,4-Oxadiazole 

As shown in Scheme 1, 1,2,4-oxadiazole series compounds **4a**–**4w** and **5a**–**5e** containing anisic acid or cinnamic acid were synthesized. Compounds **2a**–**2b** used substituted benzaldehyde as the starting material and were heated at 100 °C in the mixture of DMSO and hydroxylamine hydrochloride until no bubbles were formed. *E*-*N*′-hydroxybenzimidamide (amidoxime **3a**–**3d**) was obtained by the reaction of **2a–2b** with hydroxylamine hydrochloride under alkaline conditions. Then, in anhydrous DMF, compounds **4a**–**4w** and **5a**–**5e** were obtained by reacting with different anisic acid or cinnamic acid derivatives with EDCI, HOBt, and TEA as the condensation agent. The specific synthesis and purification steps are listed in the experimental section, and the structure of the obtained compounds is shown in Table S1.

### 2.2. Cytotoxicity Evaluation

**Figure 1:**
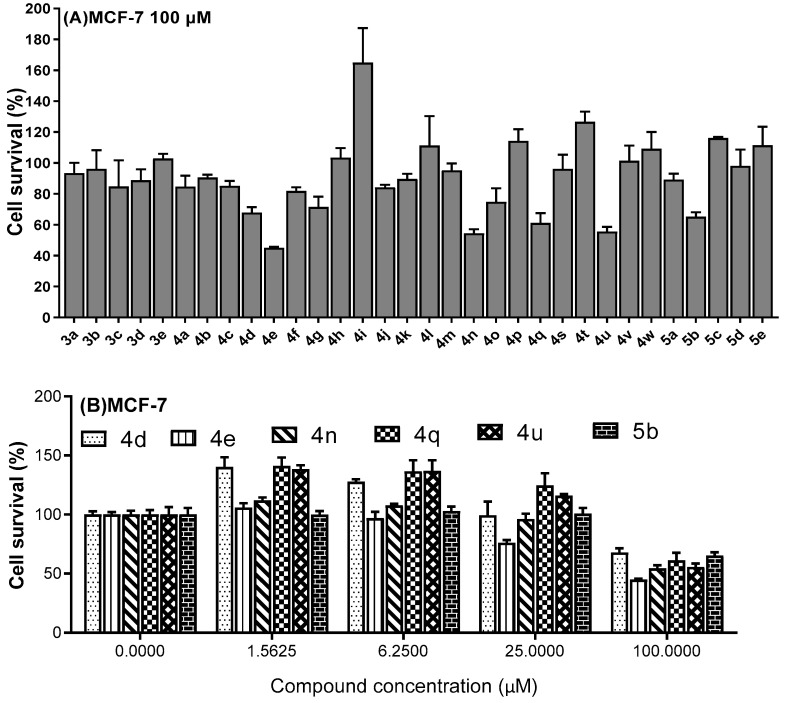
(**A**) Cytotoxicity of the target compounds on MCF-7 at 100 μM. (**B**) Cytotoxicity of the target compounds on MCF-7 at 0~100 μM (cytotoxicity of target compounds toward cells were determined by MTT assay, and data are presented as mean ± SD for three independent tests).

As the target compound of antifungal compounds, the compound will eventually be applied to the environment. In order to preliminarily analyze the impact of the compound on environmental organisms, the cytotoxicity of the target compound to the MCF-7 cell line was evaluated by the MTT method. As shown in Figure 1A, all compounds except compounds **4d**, **4e**, **4n**, **4q**, **4u**, and **5b** showed no cytotoxicity to MCF-7 cells at 100 μM, and the cell survival rate of MCF-7 cells could be maintained above 80%. In addition, some compounds such as **4i**, **4l**, **4p**, **4t**, **5c**, and **5e** even showed significant cell proliferation. To further illustrate the low toxicity of the compound, the experiment was carried out on **4d**, **4e**, **4n**, **4q**, **4u**, and **5b** in the range of 0~100 μM. It was found that the inherent cytotoxicity of these compounds to MCF-7 cells was low (IC_50_s ≥ 100 μM). Therefore, the MTT method preliminarily determined that the compound had relatively low toxicity in the 100 μM concentration order.

### 2.3. In Vitro Fungicidal Activities and Structure–Activity Relationship

The in vitro antifungal activity of the compound against three plant pathogens was evaluated at 50 μg/mL. It can be seen from Table 1 that the target compound showed different levels of antifungal activity. The structure–activity relationship was analyzed according to the experimental results. Firstly, the remarkable feature is that the activity of the derivatives containing anisic acid is superior to that containing cinnamic acid in all compounds. Among cinnamic acid derivatives, the inhibition rates of compounds **5b** and **5e** with methoxy substitution in the aromatic ring structure are better than those of chlorine substituted **5c** and unsubstituted **5a**. As shown in Table 1, the substituents of Ar_1_ were fixed to synthesize compounds **4a**–**4i**. When the benzene of Ar_2_ was monosubstituted, the inhibition rate of **4d** (*m*-OH) was superior to that of **4c** (*p*-C(CH_3_)_3_) and **4a** (*p*-Cl), and the activities of **4h** (*p*-C(CF_3_)_3_) and **4i** (*p*-NO_2_) were the lowest. When the benzene of Ar_2_ is polysubstituted, the activity of **4f** (3,4-2OH) is better than **4b** (3,4-2OCH_3_), **4e** (3,5-2OH), and **4g** (3,4,5-3OH). The antifungal activity of the active compounds against *F. graminearum*, *E. turcicum*, *C. capsica*, and *R. solani* was better than that of *B. cinerea*. When Ar_1_ is changed to the mono-methoxy-substituted benzene ring or dimethoxy-substituted benzene ring, the conclusion is almost identical, and the activity of **4q** (3,4-2OH) is the best. When Ar_2_ is fixed and the Ar_1_ structure changes, the change law is consistent. The activity of unsubstituted compounds is better than that of mono-methoxy-substituted compounds, while dimethoxy-substituted and trimethoxy-substituted compounds had the lowest activity. Overall, compounds **4f** and **4q** showed the best antifungal activity.

**Figure 2:**
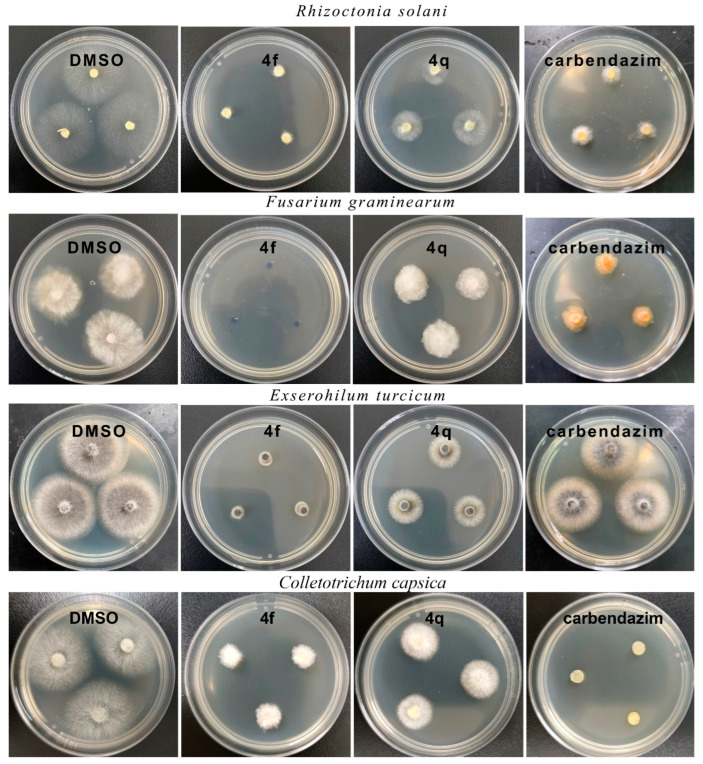
Antifungal activity test with mycelia growth inhibitory rate methods at 50 μg/mL.

Figure 2 exhibits the mycelial growth of the DMSO group, **4f** group, **4q** group, and positive drug group at drug concentration of 50 μg/mL against three plant pathogens. As can be seen from Figure 2, mycelia in the DMSO group grew faster with a good state, and the positive drug inhibited the three plant pathogens to varying degrees. At this concentration level, the inhibitory effect of **4q** against *F. graminearum*, C*. capsica*, and *B. cinerea* was slightly lower than that of positive drugs, but stronger than that of the positive drug against *E. turcicum*. It is gratifying that the inhibitory effect of **4f** on the five fungi is better than that of positive drugs at high concentration levels.

### 2.4. EC_50_ of Compounds Against Plant-Pathogenic Fungi

In order to screen the compounds with better activity, the *EC*_50_ values of potential compounds **4f** and **4q** against four plant pathogens (*R. solani*, *F. graminearum*, *E. turcicum*, and *C. capsica*) were further calculated and compared with the positive compound carbendazim. In the regression equation (Table 2), *x* represents the logarithm of molar concentration (lg*C*), and *y* represents the probability value of average inhibition rate. Table 2 manifests that compound **4q** has remarkable inhibitory activity against *R. solani*, *F. graminearum*, *E. turcicum*, and *C. capsica*. The *EC*_50_ values were 38.88, 149.26, 228.99, and 41.67 μg/mL against four pathogens, respectively, especially against *R. solani* and *C. capsica*. Compound **4g** showed a significant inhibitory effect against *F. Graminearum*, with an *EC*_50_ value of 80.55 μg/mL. Similarly, compound **4f** was also prominent against four fungi, with *EC*_50_ of 12.68, 29.97, 29.14, and 8.81 μg/mL, respectively. In particular, **4f** showed the best inhibition rate against *E. turcicum* (*EC*_50_ 29.14 μg/mL), which was much better than carbendazim (109.56 μg/mL). Therefore, **4f** was selected as the most promising candidate for further research.

### 2.5. Effect of Compounds on Plant Pathogen Morphology

**Figure 3:**
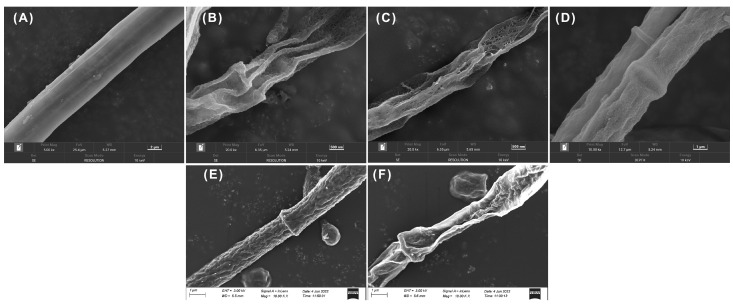
Effect of compounds on *E. turcicum. Blank* (**A**), **4f** (25 μg/mL) (**B**), **4q** (50 μg/mL) (**C**), and carbendazim (12.5 μg/mL) (**D**). Effect of compounds on *C. capsica. Blank* (**E**) and **4f** (25 μg/mL) (**F**).

Considering that **4f** and **4q** presented the strongest antifungal activity against *E. turcicum*, the effect of this compound and carbendazim on the morphology of this pathogen was studied with SEM. As can be seen from Figure 3A, the mycelial structure of the blank sample is full, linear, and homogeneous. When the mycelium was exposed to the positive drug, there was some shrinkage on the mycelium surface (Figure 3D). When the fungus was treated with **4f** (25 μg/mL) or **4q** (50 μg/mL), the mycelium structure was crinkled, collapsed, broken, and ruptured, and the morphology was seriously damaged (Figure 3B,C). These results indicated that compounds **4f** and **4q** could induce the physiological changes in fungal hyphae (*E. turcicum*), leading to the damage of cell morphology and the death of fungi. Similar results were observed during the study of the effects of **4f** (25 μg/mL) on the morphology of *C. capsica* (Figure 3E,F).

### 2.6. The Effect of Antifungal Activity In Vivo

The protective effects of compounds **4q** and **4f** and carbendazim on pepper infected by *C. capsica* were studied. The blank control was 0.1% tween-80 aqueous solution, and the treatment group was diluted to 500 μg/mL and 1000 μg/mL by 0.1% tween-80 aqueous solution from 10 mg/mL

**Figure 4:**
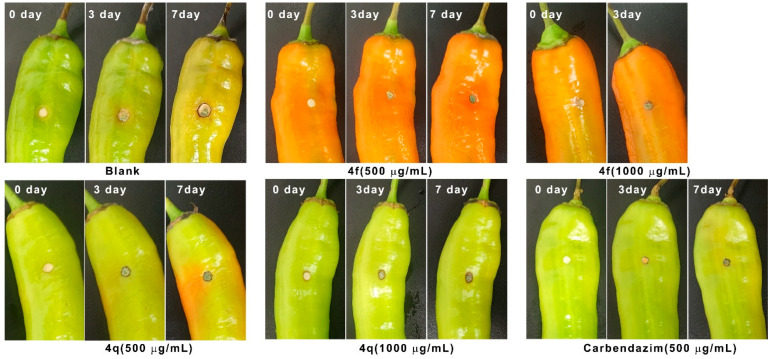
Protective and curative activity of compounds against *C. capsica*.

It can be seen from Figure 4 and Table 3 that compounds **4q** and **4f** have a certain protective effect on *C. capsica* in vivo, among which compound **4f** is the most effective, and the control efficacy for both reached 100% at the concentration of 500 μg/mL and 1000 μg/mL, which was equal to that of the positive drug. The control efficacy of compound **4q** at 1000 μg/mL was 76.83%, slightly lower than that of the positive drug.

### 2.7. Analysis of Molecular Docking

**Figure 5:**
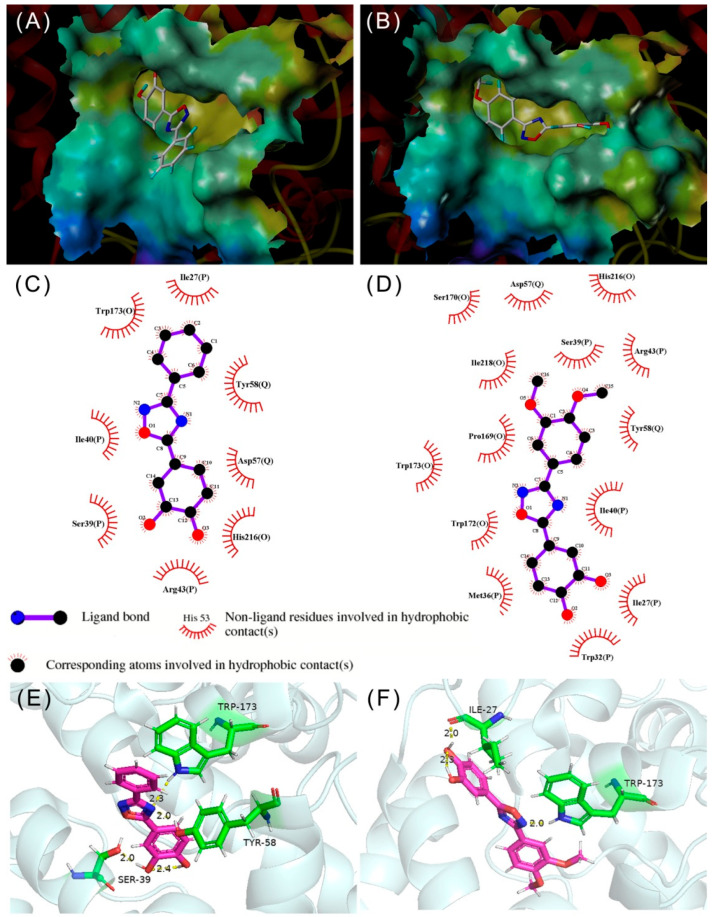
Three-dimensional docking of compounds with the active pocket of protein 2fbw (**A**,**B**). Two-dimensional ligand hydrophobic interaction (**C**,**D**) of compounds with amino acid residues. Hydrogen bond interaction of compounds with amino acid residues (**E**,**F**).

In order to study the possible mechanism of antifungal activity of compounds, the relationship between compounds and SDH (PDB Code: 2fbw) was studied by Sybyl [38]. Figure 5 and Figure S5 illustrate the hydrogen bond interactions between **4f, 4q**, and carbendazim with amino acid residues, the 3D docking of compounds with the active pocket of protein 2fbw, and the 2D ligand hydrophobic interactions between compounds and amino acid residues.

It can be seen from Figure 5E that the nitrogen atom on the oxadiazole ring of **4f** can form a hydrogen bond with the phenolic hydroxyl on TYR58 and the amino of the indole ring on TRP173, with 2.0 Å and 2.3 Å bond lengths. The phenolic hydroxyl hydrogen atom of **4f** can form a hydrogen bond with the hydroxyl on SER39 with a bond length of 2.0 Å. In addition, **4f** forms a hydrophobic interaction with TRP173, ILE27, TYR58, ASP57, HIS216, ARG43, SER39, and ILE40 (Figure 5C).

The nitrogen atom on the oxadiazole ring of **4q** can form the amino of the indole ring on TRP173, with a bond length of 2.0 Å (Figure 5F). The phenolic hydroxyl hydrogen atom of **4q** can form a hydrogen bond with the carbonyl oxygen atom of the peptide bond on ILE27, with a bond length of 2.0 Å (Figure 5F). In addition, **4q** forms a hydrophobic interaction with TRP173, PRO169, ILE218, SER170, ASP57, SER39, HIS216, ARG43, TYR58, ILE40, ILE27, TRP32, MET36, and TRP172 (Figure 5D). Carbendazim may form two hydrogen bonds with SER39, with bond lengths of 2.0 Å and 2.8 Å, and it also forms hydrophobic interactions with TRP173, PRO169, SER39, ARG43, HIS216, ILE218, ILE40, and MET36 (Figure S5).The hydrogen bonding, hydrophobic interaction, and spatial complementarity between the ligand and amino acid residues enable the ligand to be perfectly embedded in the hydrophobic pocket (Figure 5A,B). The total score function of the Surflex-Dock molecular docking module was used to score the interaction between small molecules and target proteins. The greater the total score value, the better the matching and binding between small molecular compounds and macromolecular proteins. The total score for the docking of **4f** and **4q** with SDH molecules were 6.0886 and 5.4148, respectively, while the docking score for carbendazim was only 4.3215, further indicating that **4f** and **4q** have strong binding with SDH.

## 3. Experimental Section

### 3.1. Chemistry

All the chemicals were commercially procured from J&K Scientific Ltd. (Beijing, China). The melting points were taken on a micro melting point instrument (JHX-4B, Shanghai Jiahang Instruments Co., Ltd., Shanghai, China). NMR spectra were recorded on a Bruker Avance 400 spectrometer (Bruker, Billerica, MA, USA, 400 MHz for ^1^H NMR, 101 MHz for ^13^C NMR). Yields are given after purification. ESI-MS spectra were obtained using an Agilent triple-quadrupole system (6460, Agilent Technologies, Santa Clara, CA, USA) equipped by Electrospray Source ESI operating in both positive and negative ions. The purity of the compounds was checked by thin-layer chromatography (TLC) with silica gel plates. Molecular docking was performed with Surflex-Dock implemented in SYBYL (version 2.1.1, Tripos, Inc, St. Louis, MO, USA).

#### 3.1.1. Synthesis of Benzonitrile

Hydrochloric acid chloride (2.55 g, 37 mmol) was suspended in DMSO (25 mL), and benzaldehyde was added with stirring. The mixture was heated to reflux at 100 °C until the reaction system was free of bubbles. After cooling to room temperature, the mixture was slowly poured into 100 mL of ice water, and the solid **1c**–**1e** was filtered from the mixture and washed twice with petroleum ether.

Methoxybenzonitrile (**2c**) was prepared from 4-methylbenzaldehyde (2.72 g, 20 mmol)—white solid, yield 95%.

3,4-dimethoxybenzonitrile (**2d**) was prepared from 3,4-dimethoxybenzaldehyde (3.32 g, 20 mmol)—white solid, yield 95%. *C*alculated *m*/*z* for C_9_H_9_NO_2_: 163.06), found: 163.09. 

3,4,5-trimethoxybenzonitrile (**2e**) was prepared from 3,4,5-trimethoxybenzaldehyde (3.92 g, 20 mmol)—white solid, yield 95%. *C*alculated *m*/*z* for C_10_H_11_NO_3_: 193.07), found: 193.09.

#### 3.1.2. Synthesis of (*E*)-*N*′-Hydroxybenzimidamide

The arylamidoximes were synthesized according to the method reported in the literature [39]. A suitable amount of benzonitrile (**2a**–**2e**) was dissolved in 95% ethanol (30 mL), and hydroxylamine hydrochloride (1.39 g, 20 mmol) and 10% potassium hydroxide (10 mL) were added with stirring. The vessel was placed in an oil bath and refluxed at 95 °C for 4 h. After cooling to room temperature, the mixture was concentrated to dryness. The obtained solid was suspended in ethyl acetate, the insoluble material was removed by filtration, and the filtrate was collected and concentrated under reduced pressure to give solid. The resulting crude product was purified as follows.

(*E*)-*N*′-Hydroxybenzimidamide (**3a**)

Prepared from **2a** (1.03 g, 10 mmol). Purification procedure: column chromatography (eluent: ethyl acetate in petroleum ether 65%). White solid, yield 85%. m.p.: 67.3–69.3 °C. ^1^H NMR (400 MHz, DMSO-*d*_6_) δ 9.64 (s, 1*H*, –O*H*), 7.70–7.65 (m, 2*H*, Ar-*H*), 7.39–7.36 (m, 3*H*, Ar-*H*), 5.82–5.77 (d, 2*H*, –N*H*_2_). Calculate *m*/*z* for C_7_H_8_N_2_O: 136.15, found: 136.09.

(*E*)-*N*′-Hydroxy-4-methylbenzimidamide (**3b**)

Prepared from **2b** (1.51 g, 10 mmol). Purification procedure: column chromatography (eluent: ethyl acetate in petroleum ether 65%).White solid, yield 87%. m.p.: 143.5–145.1 °C; ^1^H NMR (400 MHz, DMSO-*d*_6_) δ 9.54 (s, 1*H*, –O*H*), 7.57–7.55 (d, 2*H*, Ar-*H*), 7.19–7.17(d, 2*H*, Ar-*H*), 5.76 (s, 2*H*, –N*H*_2_), 2.31(s, 3*H*, –C*H*_3_). Calculate *m*/*z* for C_8_H_10_N_2_O: 150.18, found: 150.09.

(*E*)-*N*′-Hydroxy-4-methoxybenzimidamide (**3c**)

Prepared from **2c** (1.36 g, 10 mmol). Purification procedure: column chromatography (eluent: ethyl acetate in petroleum ether 60%). White solid, yield 82%. m.p.: 119.5–121.2 °C. ^1^H NMR (400 MHz, DMSO-*d*_6_) δ 9.47 (s, 1*H*, –O*H*), 7.62–7.60 (d, 2*H*, Ar-*H*), 6.94–6.92 (d, 2*H*, Ar-*H*), 5.74 (s, 2*H*, –N*H*_2_), 3.77 (s, 3*H*, –OC*H*_3_). Calculate *m*/*z* for C_8_H_10_N_2_O_2_: 166.18, found: 166.09.

(*E*)-*N*′-Hydroxy-3,4-dimethoxybenzimidamide (**3d**)

Prepared from **2d** (1.63 g, 10 mmol). Purification procedure: column chromatography (eluent: ethyl acetate in petroleum ether 60%). White solid, yield 88%. m.p.: 163.2–164.5 °C. ^1^H NMR (400 MHz, DMSO-*d*_6_) δ 9.47 (s, 1*H*, –O*H*), 7.25–7.22 (t, 2*H*, Ar-*H*), 6.95–6.93 (d, 1*H*, Ar-*H*), 5.765.74(s, 2*H*, –N*H*_2_), 3.77(s, 6*H*, –OC*H*_3_). Calculate *m*/*z* for C_9_H_12_N_2_O_3_: 196.20, found: 196.01.

(*E*)-*N*′-Hydroxy-3,4,5-trimethoxybenzimidamide (**3e**)

Prepared from **2e** (1.93g, 10 mmol). Purification procedure: column chromatography (eluent: ethyl acetate in petroleum ether 60%). White solid, yield 81%. m.p.: 168.2–169.1 °C. ^1^H NMR (400 MHz, DMSO-*d*_6_) δ 9.57 (s, 1*H*, –O*H*), 6.99 (s, 2*H*, Ar-*H*), 5.84 (s, 2*H*, –N*H*_2_), 3.79 (s, 6*H*, –OC*H*_3_), 3.67(s, 3*H*, –OC*H*_3_). Calculate *m*/*z* for C_10_H_14_N_2_O_4_: 226.23, found: 226.09.

#### 3.1.3. Synthesis of 1,2,4-Oxadiazoles

An appropriate amount of carboxylic acid was dissolved in anhydrous DMF (20 mL), followed by the addition of EDCI, HOBt, and 100 μL anhydrous TEA. The appropriate **3a**–**3d** were added and sonicated until they were completely dissolved. After refluxing at 140 °C for 1 h, the mixture was cooled to room temperature, and the distilled water (30 mL) was added. The resulting solution was extracted with ethyl acetate (3 × 20 mL), washed with brine, and dried over anhydrous Na_2_SO_4_, and the solvent was removed by a rotary evaporator. The resulting crude solid was purified as detailed below.

5-(4-Chlorophenyl)-3-phenyl-1,2,4-oxadiazole (**4a**)

Prepared from **2a** (0.20 g, 1.47 mmol), 4-chlorobenzoic acid (0.18 g, 1.15 mmol), EDCI (0.25 g, 1.30 mmol), HOBt (0.17 g, 1.26 mmol). Purification procedure: column chromatography (eluent: ethyl acetate in petroleum ether 14.3% with 1% TEA). White solid, yield 65%. m.p.: 120.3–123.4 °C. ^1^H NMR (400 MHz, CDCl_3_) δ 8.20–8.18 (m, 4*H*, Ar-*H*), 7.58–7.52 (m, 5*H*, Ar-*H*). ^13^C NMR (101 MHz, CDCl_3_) δ 174.78, 169.03, 139.19, 131.33, 129.53, 129.46, 129.43, 128.90, 127.52, 127.47, 126.73, 122.73. Calculate *m*/*z* for C_14_H_9_ClN_2_O: 256.04, found: 256.09.

5-(3,4-Dimethoxyphenyl)-3-phenyl-1,2,4-oxadiazole (**4b**)

Prepared from **2a** (0.25 g, 1.84 mmol), 3,4-dimethoxybenzoic acid (0.16 g, 0.88 mmol), EDCI (0.34 g, 1.77 mmol), HOBt (0.24 g, 1.78 mmol). Purification procedure: column chromatography (eluent: ethyl acetate in petroleum ether 50% with 1% TEA). Light yellow solid, yield 60%. m.p.: 104.9–106.7 °C. ^1^H NMR (400 MHz, CDCl_3_) δ 8.21–8.17 (m, 2*H*, Ar-*H*), 7.88–7.86 (q, 1*H*, Ar-*H*), 7.72 (d, 1*H*, Ar-*H*) 7.56–7.46 (m, 3*H*, Ar-*H*), 7.04–7.02 (d, 1*H*, *Ar*-*H*), 4.04–4.01 (d, 6*H*, –OC*H*_3_) ^13^C NMR (101 MHz, CDCl_3_) δ 175.60, 168.85, 152.79, 149.23, 131.13, 128.84, 128.82, 128.62, 127.51, 127.17, 127.06, 122.07, 116.85, 111.05, 110.39, 56.17, 56.11. Calculate *m*/*z* for C_16_H_14_N_2_O_3_: 282.10, found: 282.19.

5-(4-(tert-Butyl)phenyl)-3-phenyl-1,2,4-oxadiazole (**4c**)

Prepared from **2a** (0.50 g, 3.67 mmol), 4-(tert-butyl) benzoic acid (0.49 g, 2.75 mmol), EDCI (0.64 g, 3.34 mmol), HOBt (0.45 g, 3.33 mmol). Purification procedure: column chromatography (eluent: ethyl acetate in petroleum ether 14.3% with 1% TEA). Light yellow solid, yield 45%. m.p.: 50.5–51.8 °C. ^1^H NMR (400 MHz, CDCl_3_) δ 8.22–8.14 (m, 4*H*, Ar-*H*), 7.61–7.51 (m, 5*H*, Ar-*H*), 1.40–1.37 (d, 9*H,* –C(C*H*_3_)_3_). ^13^C NMR (101 MHz, CDCl_3_) δ 175.79, 168.90, 156.49, 131.13, 128.84, 128.03, 127.54, 127.09, 126.11, 121.52, 35.23, 31.12. Calculate *m*/*z* for C_18_H_18_N_2_O: 278.14, found: 278.09.

3-(3-Phenyl-1,2,4-oxadiazol-5-yl) phenol (**4d**)

Prepared from **2a** (0.50 g, 3.67 mmol), 3-hydroxybenzoic acid (0.43 g, 3.12 mmol), EDCI (0.70 g, 3.65 mmol), HOBt (0.50 g, 3.70 mmol). Purification procedure: column chromatography (eluent: ethyl acetate in petroleum ether 25%). White solid, yield 78%. m.p.: 184.3–186.5°C. ^1^H NMR (400 MHz, DMSO-*d*_6_) δ 10.13(s, 1*H*, –O*H*), 8.12–8.09 (m, 2*H*, Ar-*H*), 7.64–7.57 (m, 5*H*, Ar-*H*), 7.50–7.46 (t, 1*H*, Ar-*H*), 7.14–7.11 (q, 1*H*, Ar-*H*). ^13^C NMR (101 MHz, DMSO-*d*_6_) δ 176.01, 168.65, 158.49, 132.10, 131.31, 129.73, 128.65, 127.91, 127.54, 126.64, 124.81, 120.95, 119.04, 114.57. Calculate *m*/*z* for C_14_H_10_N_2_O_2_: 238.07, found: 237.99.

5-(3-Phenyl-1,2,4-oxadiazol-5-yl) benzene-1,3-diol (**4e**)

Prepared from **2a** (0.20 g, 1.47 mmol), 3,5-dihydroxybenzoic acid (0.19 g, 1.23 mmol), EDCI (0.28 g, 1.46 mmol), HOBt (0.20 g, 1.47 mmol). Purification procedure: column chromatography (eluent: ethyl acetate in petroleum ether 25%). Light yellow solid, yield 33%. m.p.: 265.0–267.2 °C. ^1^H NMR (400 MHz, DMSO-*d*_6_) δ 9.96 (s, 2*H*, –O*H*), 8.09–8.07 (m, 2*H*, Ar-*H*), 7.64–7.58 (m, 3*H*, Ar-*H*), 7.05–7.04 (d, 2*H*, Ar-*H*), 6.54–6.53 (t, 1*H*, Ar-*H*). ^13^C NMR (101 MHz, DMSO-*d*_6_) δ 176.07, 168.60, 159.71, 132.09, 129.73, 128.65, 127.90, 127.53, 126.67, 125.06, 107.75, 106.18. Calculate *m*/*z* for C_14_H_10_N_2_O_3_: 254.07, found: 254.11.

4-(3-Phenyl-1,2,4-oxadiazol-5-yl) benzene-1,2-diol (**4f**)

Prepared from **2a** (0.24 g, 1.76 mmol), 3,4-dihydroxybenzoic acid (0.19 g, 1.25 mmol), EDCI (0.28 g, 1.46 mmol), HOBt (0.20 g, 1.48 mmol). Purification procedure: column chromatography (eluent: ethyl acetate in petroleum ether 50%). Light yellow solid, yield 42%. m.p.: 237.5–239.2 °C. ^1^H NMR (400 MHz, DMSO-*d*_6_) δ 9.89 (s, 3*H*, –O*H*), 8.09–8.06 (m, 2*H*, Ar-*H*), 7.62–7.52 (m, 5*H*, Ar-*H*), 6.98–6.96 (d, 1*H*, Ar-*H*). ^13^C NMR (101 MHz, DMSO-*d*_6_) δ 176.12, 168.43, 151.25, 146.44, 131.92, 129.66, 127.48, 126.92, 120.98, 116.77, 115.08, 114.63. Calculate *m*/*z* for C_14_H_10_N_2_O_3_: 254.07, found: 253.99.

5-(3-Phenyl-1,2,4-oxadiazol-5-yl) benzene-1,2,3-triol (**4g**)

Prepared from **2a** (0.40 g, 2.94 mmol), 3,4,5-trihydroxybenzoic acid (0.21 g, 1.24 mmol), EDCI (0.28 g, 1.46 mmol), HOBt (0.20 g, 1.48 mmol). Purification procedure: column chromatography (eluent: 11% Methanol, 44% CH_2_Cl_2_ in petroleum ether with 2% HAc). Grey solid, yield 37%. m.p.: 259.3–262.2 °C. ^1^H NMR (400 MHz, DMSO-*d*_6_) δ 9.50 (s, 3*H*, –O*H*), 8.07–8.05 (m, 2*H*, Ar-*H*), 7.61–7.59 (d, 3*H*, Ar-*H*), 7.15 (s, 2*H*, Ar-*H*) ^13^C NMR (101 MHz, DMSO-*d*_6_) δ 176.30, 168.42, 146.94, 139.20, 131.94, 129.69, 127.47, 126.93, 113.42, 107.46. Calculate *m*/*z* for C_14_H_10_N_2_O_4_: 270.06, found: 269.99.

3-Phenyl-5-(4-(trifluoromethyl)phenyl)-1,2,4-oxadiazole (**4h**)

Prepared from **2a** (0.22 g, 1.62 mmol), 4-(trifluoromethyl) benzoic acid (0.26 g, 1.37 mmol), EDCI (0.35 g, 1.83 mmol), HOBt (0.25 g, 1.85 mmol). Purification procedure: column chromatography (eluent: ethyl acetate in petroleum ether 50%). White solid, yield 55%. m.p.: 101.1–101.8 °C. ^1^H NMR (400 MHz, CDCl_3_) δ 8.39–8.37 (d, 2*H*, Ar-*H*), 8.22–8.19 (q, 2*H*, Ar-*H*), 7.87–7.85 (d, 2*H*, Ar-*H*), 7.57–7.55 (m, 3*H*, Ar-*H*). ^13^C NMR (101 MHz, DMSO-*d*_6_) δ 174.69, 168.89, 133.27, 132.95, 132.30, 129.79, 129.33, 127.59, 127.49, 126.98, 126.94, 126.32. Calculate *m*/*z* for C_15_H_9_F_3_N_2_O: 290.07, found: 291.07.

5-(4-Nitrophenyl)-3-phenyl-1,2,4-oxadiazole (**4i**)

Prepared from **2a** (0.23 g, 1.70 mmol), 4-nitrobenzoic acid (0.21 g, 1.26 mmol), EDCI (0.28 g, 1.46 mmol), HOBt (0.21 g, 1.55 mmol). Purification procedure: column chromatography (eluent: ethyl acetate in petroleum ether 18%). White solid, yield 77%. m.p.: 223.0–225.2 °C. ^1^H NMR (400 MHz, CDCl_3_) δ 8.45 (s, 4*H*, -Ar-*H*), 8.22–8.20(q, 2*H*, -Ar-*H*), 7.59–7.55 (m, 3*H*, -Ar-*H*). ^13^C NMR (101 MHz, CDCl_3_) δ 173.64, 169.41, 150.17, 131.62, 129.54, 129.23, 129.01, 127.56, 126.31, 124.39. Calculate *m*/*z* for C_14_H_9_N_3_O_3_: 267.06, found: 268.07.

5-(4-Chlorophenyl)-3-(4-methoxyphenyl)-1,2,4-oxadiazole (**4j**)

Prepared from **2b** (0.50 g, 3.01 mmol), 4-chlorobenzoic acid (0.39 g, 2.50 mmol), EDCI (0.57 g, 2.97 mmol), HOBt (0.41 g, 3.03 mmol). Purification procedure: column chromatography (eluent: ethyl acetate in petroleum ether 14% with 1% TEA). White solid, yield 55%. m.p.: 134.5–134.9 °C. ^1^H NMR (400 MHz, CDCl_3_) δ 8.19–8.11 (q, 4*H*, Ar-*H*), 7.57–7.55 (d, 2*H*, Ar-*H*), 7.05–7.03 (d, 2*H*, Ar-*H*), 3.91 (s, 3*H*, –OC*H*_3_). ^13^C NMR (101 MHz, CDCl_3_) δ 174.50, 168.72, 161.99, 139.06, 129.48, 129.43, 129.39, 129.13, 129.09, 122.83, 119.17, 114.27, 114.22, 55.42. Calculate *m*/*z* for C_15_H_11_ClN_2_O_2_: 286.05, found: 286.09.

5-(3,4-Dimethoxyphenyl)-3-(4-methoxyphenyl)-1,2,4-oxadiazole (**4k**)

Prepared from **2b** (0.50 g, 3.01 mmol), 3,4-dimethoxybenzoic acid (0.46 g, 2.53 mmol), EDCI (0.57 g, 2.97 mmol), HOBt(0.41 g, 3.03 mmol). Purification procedure: column chromatography (eluent: ethyl acetate in petroleum ether 50% with 1% TEA). Light red solid, yield 44%. m.p.: 123.3–124.5 °C. ^1^*H* NMR (400 MHz, CDCl_3_) δ 8.14–8.12 (m, 2*H*, *Ar*-*H*), 7.87–7.84 (q, 1*H*, *Ar*-*H*), 7.71 (d, 1*H*, *Ar*-*H*), 7.05–7.01 (m, 3*H, Ar*-*H*), 4.04–4.00 (d, 6*H*, –OC*H*_3_), 3.91 (s, 3*H*, –OC*H*_3_). ^13^C NMR (101 MHz, CDCl_3_) δ 175.31, 168.53, 161.84, 152.71, 149.19, 129.10, 122.01, 119.51, 116.95, 114.19, 111.02, 110.38, 56.16, 56.09, 55.39. Calculate *m*/*z* for C_17_H_16_N_2_O_4_: 312.11, found: 312.19.

5-(4-(tert-Butyl)phenyl)-3-(4-methoxyphenyl)-1,2,4-oxadiazole (**4l**)

Prepared from **2b** (0.50 g, 3.01 mmol), 4-(tert-butyl) benzoic acid (0.45 g, 2.53 mmol), EDCI (0.57 g, 2.97 mmol), HOBt (0.41 g, 3.03 mmol). Purification procedure: column chromatography (eluent: ethyl acetate in petroleum ether 20% with 1% TEA). Light yellow solid, yield 44%. m.p.: 77.5–81.0 °C. ^1^H NMR (400 MHz, CDCl_3_) δ 8.17–8.13 (m, 4*H*, Ar-*H*), 7.60–7.58 (d, 2*H*, Ar-*H*), 7.05–7.03 (m, 2*H*, Ar-*H*), 3.91 (s, 3*H*, –OC*H*_3_), 1.40 (s, 9*H*, –C(C*H*_3_)_3_). ^13^C NMR (101 MHz, CDCl_3_) δ 175.51, 168.59, 161.86, 156.36, 129.13, 127.99, 126.07, 121.60, 119.54, 114.21, 55.40, 35.21, 31.12. Calculate *m*/*z* for C_19_H_20_N_2_O_2_: 308.15, found: 308.19.

5-(4-Chlorophenyl)-3-(3,4-dimethoxyphenyl)-1,2,4-oxadiazole (**4m**)

Prepared from **2c** (0.30 g, 1.53 mmol), 4-chlorobenzoic acid (0.20 g, 1.28 mmol), EDCI (0.28 g, 1.46 mmol), HOBt (0.20 g, 1.48 mmol). Purification procedure: column chromatography (eluent: ethyl acetate in petroleum ether 25% with 1% TEA). White solid, yield 52%. m.p.: 153.4–154.3 °C. ^1^*H* NMR (400 MHz, CDCl_3_) δ 8.19–8.17 (d, 2*H*, Ar-*H*), 7.81–7.79 (q, 1*H*, Ar-*H*), 7.67 (d, 1*H*, Ar-*H*), 7.58–7.54 (m, 2*H*, Ar-*H*), 7.02–6.96 (q, 1*H*, Ar-*H*), 4.02–3.97 (t, 6*H*, –OC*H*_3_). ^13^C NMR (101 MHz, CDCl_3_) δ 174.55, 168.79, 151.56, 149.14, 139.12, 129.49, 129.44, 122.76, 120.98, 119.25, 110.99, 109.82, 56.07, 55.99. Calculate *m*/*z* for C_16_H_13_ClN_2_O_3_: 316.06, found: 316.09.

5-(4-(tert-Butyl)phenyl)-3-(3,4-dimethoxyphenyl)-1,2,4-oxadiazole (**4n**)

Prepared from **2c** (0.20 g, 1.02 mmol), 4-(tert-butyl)benzoic acid (0.15 g, 0.84 mmol), EDCI (0.20 g, 1.04 mmol), HOBt (0.14 g, 1.04 mmol). Purification procedure: column chromatography (eluent: ethyl acetate in petroleum ether 50% with 1% TEA). Light yellow solid, yield 52.2%. m.p.: 98.1–101.5 °C. ^1^H NMR (400 MHz, CDCl_3_) δ 8.18–8.16 (d, 2*H*, -Ar-*H*), 7.84–7.81 (q, 1*H*, -Ar-*H*), 7.70–7.69 (d, 1*H*, -Ar-*H*), 7.60–7.58 (d, 3*H*, -Ar-*H*), 7.02–7.00 (d, 1*H*, -Ar-*H*), 4.03 (s, 3*H*, –OC*H*_3_), 3.99 (s, 3*H*, –OC*H*_3_), 1.40 (s, 9*H*, –C(C*H*_3_)_3_). ^13^C NMR (101 MHz, CDCl_3_) δ 175.58, 168.66, 156.44, 151.40, 149.09, 128.01, 126.08, 121.52, 120.96, 119.62, 110.96, 109.87, 56.08, 56.03, 55.99, 35.21, 31.11. Calculate *m*/*z* for C_20_H_22_N_2_O_3_: 338.16, found: 338.19.

3-(3-(3,4-Dimethoxyphenyl)-1,2,4-oxadiazol-5-yl)phenol (**4o**)

Prepared from **2c** (0.3 g, 1.53 mmol), 3-hydroxybenzoic acid (0.18 g, 1.17 mmol), EDCI (0.28 g, 1.46 mmol), HOBt (0.21 g, 1.55 mmol). Purification procedure: column chromatography (eluent: 33% ethyl acetate and 33% CH_2_Cl_2_ in petroleum ether). White solid, yield 34%. m.p.: 177.0–178.0 °C. ^1^H NMR (400 MHz, CDCl_3_) δ 7.82–7.78 (m, 3*H*, Ar-*H*), 7.66 (d, 1*H*, Ar-*H*), 7.46–7.42 (q, 1*H*, Ar-*H*), 7.13–7.11 (m, 1*H*, Ar-*H*), 7.00–6.98 (d, 1*H*, Ar-*H*), 4.00–3.97 (d, 6*H*, Ar-*H*). ^13^C NMR (101 MHz, DMSO-*d*_6_) δ 175.57, 168.47, 158.46, 151.95, 149.43, 131.27, 124.87, 120.96, 120.84, 119.01, 118.85, 114.54, 112.29, 110.01, 56.06, 55.99. Calculate *m*/*z* for C_16_H_14_N_2_O_4_: 298.10, found: 298.21.

5-(3-(3,4-Dimethoxyphenyl)-1,2,4-oxadiazol-5-yl)benzene-1, 3-diol (**4p**)

Prepared from **2c** (0.30 g, 1.53 mmol), 3,5-dihydroxybenzoic acid (0.20 g, 1.30 mmol), EDCI (0.28 g, 1.46 mmol), HOBt (0.21 g, 1.55 mmol). Purification procedure: column chromatography (eluent: 33% ethyl acetate in petroleum ether). Light yellow solid, yield 51%. m.p.: 140.4–151.0 °C. ^1^H NMR (400 MHz, DMSO-*d*_6_) δ 9.95 (s, 2*H*, –O*H*), 7.68–7.66 (q, 1*H*, *Ar*-*H*), 7.54 (d, 1*H*, *Ar*-*H*), 7.18–7.16 (d, 1*H*, *Ar*-*H*), 7.04–7.03 (d, 2*H*, *Ar*-*H*), 6.52–6.51 (t, 1*H*, *Ar*-*H*), 3.87 (d, 6*H*,-OC*H*_3_). ^13^C NMR (101 MHz, DMSO-*d*_6_) δ 175.73, 168.43, 159.70, 151.95, 149.45, 125.13, 120.93, 118.89, 112.34, 110.01, 107.65, 106.14, 56.09, 56.00. Calculate *m*/*z* for C_16_H_14_N_2_O_5_: 314.09, found: 314.11.

4-(3-(3,4-Dimethoxyphenyl)-1,2,4-oxadiazol-5-yl)benzene-1,2-diol (**4q**)

Prepared from **2c** (0.30 g, 1.53 mmol), 3,4-dihydroxybenzoic acid (0.20 g, 1.30 mmol), EDCI (0.28 g, 1.46 mmol), HOBt (0.21 g, 1.55 mmol). Purification procedure: column chromatography (eluent: 50% ethyl acetate in petroleum ether). Grey solid, yield 34%. m.p.: 209.9–210.2 °C. ^1^H NMR (400 MHz, DMSO-*d*_6_) δ 7.67–7.65 (q, 1*H*, *Ar*-*H*), 7.55–7.51 (m, 3*H*, *Ar*-*H*), 7.17–7.14 (d, 1*H*, *Ar*-*H*), 6.96–6.94 (d, 1*H*, *Ar*-*H*), 3.87–3.85 (d, 6*H*, –OC*H*_3_) ^13^C NMR (101 MHz, DMSO-*d*_6_) δ 175.77, 168.26, 151.83, 151.05, 149.40, 146.38, 120.92, 120.86, 119.16, 116.75, 115.08, 114.75, 112.29, 110.01, 56.07, 55.99. Calculate *m*/*z* for C_16_H_14_N_2_O_5_: 314.09, found: 314.11

5-(4-Chlorophenyl)-3-(3,4,5-trimethoxyphenyl)-1,2,4-oxadiazole (**4s**)

Prepared from **2d** (0.10 g, 0.44 mmol), 4-chlorobenzoic acid (0.06 g, 0.38 mmol), EDCI (0.08 g, 0.42 mmol), HOBt (0.06 g, 0.44 mmol). Purification procedure: column chromatography (eluent: ethyl acetate in petroleum ether 67% with 1% TEA). White solid, yield 65%. m.p.: 178.7–181.0 °C. ^1^H NMR (400 MHz, CDCl_3_) δ 8.21–8.19 (d,2*H*, *Ar*-*H*), 7.58–7.56 (d, 2*H, Ar*-*H*), 7.43 (s, 2*H*, *Ar*-*H*), 4.00–3.91 (d, 9*H*, –OC*H*_3_). ^13^C NMR (101 MHz, CDCl_3_) δ 174.76, 168.86, 153.56, 140.54, 139.26, 129.54, 129.49, 122.65, 121.93, 104.55, 61.01, 56.31. Calculate *m*/*z* for C_17_H_15_ClN_2_O_4_: 346.07, found: 346.01.

5-(3,4-Dimethoxyphenyl)-3-(3,4,5-trimethoxyphenyl)-1,2,4-oxadiazole (**4t**)

Prepared from **2d** (0.10 g, 0.44 mmol), 3,4-dimethoxybenzoic acid (0.06 g, 0.33 mmol), EDCI (0.08 g, 0.42 mmol), HOBt (0.06 g, 0.44 mmol). Purification procedure: column chromatography (eluent: ethyl acetate in petroleum ether 33% with 1% TEA). White solid, yield 59%. m.p.: 158.6–158.6 °C. ^1^H NMR (400 MHz, CDCl_3_) δ 7.89–7.86 (q, 1*H*, Ar-*H*), 7.72–7.71 (d, 1*H*, Ar-*H*), 7.44 (s, 2*H*, Ar-*H*), 7.05–7.02 (d, 1*H*, Ar-*H*), 4.05 (s, 3*H*, –OC*H*_3_), 4.01 (d, 9*H*, –OC*H*_3_), 3.95 (s, 3*H*, –OC*H*_3_). ^13^C NMR (101 MHz, CDCl_3_) δ 175.59, 168.68, 153.52, 152.86, 149.24, 140.38, 122.29, 122.15, 116.75, 111.05, 110.40, 104.55, 61.00, 56.33, 56.22, 56.22, 56.13. Calculate *m*/*z* for C_19_H_20_N_2_O_6_: 372.13, found: 372.19.

5-(4-(tert-Butyl)phenyl)-3-(3,4,5-trimethoxyphenyl)-1,2,4-oxadiazole (**4u**)

Prepared from **2d** (0.50 g, 2.21 mmol), 4-(tert-butyl)benzoic acid (0.33 g, 1.85 mmol), EDCI (0.42 g, 2.19 mmol), HOBt (0.30 g, 2.22 mmol). Purification procedure: column chromatography (eluent: ethyl acetate in petroleum ether 50% with 1% TEA). White solid, yield 53%. m.p.: 101.5–102.3 °C. ^1^H NMR (400 MHz, CDCl_3_) δ 8.18–8.16 (d, 2*H*, *Ar*-*H*), 7.60–7.58 (d, 2*H*, *Ar*-*H*), 7.45 (s, 2*H*, *Ar*-*H*), 4.00 (s, 6*H*, –OC*H*_3_), 3.95 (s, 3*H*, –OC*H*_3_). ^13^C NMR (101 MHz, CDCl_3_) δ 175.76, 168.72, 156.56, 153.52, 140.39, 128.04, 126.11, 122.31, 121.42, 104.57, 61.00, 56.31, 35.23, 31.11. Calculate *m*/*z* for C_21_H_24_N_2_O_4_: 368.17, found: 368.29.

3-(3-(3,4,5-Trimethoxyphenyl)-1,2,4-oxadiazol-5-yl) phenol (**4v**)

Prepared from **2d** (0.30 g, 1.33 mmol), 3-hydroxybenzoic acid (0.16 g, 1.16 mmol), EDCI (0.25 g, 1.30 mmol), HOBt (0.18 g, 1.33 mmol). Purification procedure: column chromatography (eluent: 78% ethyl acetate in petroleum ether). White solid, yield 48%. m.p.: 182.0–183.5 °C. ^1^H NMR (400 MHz, CDCl_3_) δ 7.85–7.80 (m, 2*H*, Ar-*H*), 7.49–7.41 (m, 3*H*, Ar-*H*), 7.15–7.12 (m, 1*H*, Ar-*H*), 4.00–3.96 (d, 9*H*, –OC*H*_3_). ^13^C NMR (101 MHz, DMSO-*d*_6_) δ 175.81, 168.51, 158.48, 153.84, 140.60, 131.32, 124.74, 121.87, 120.96, 119.07, 114.57, 104.71, 60.64, 56.48. Calculate *m*/*z* for C_17_H_16_N_2_O_5_: 328.11, found: 328.01.

3-(3,4-Dimethoxyphenyl)-5-(4-methoxyphenyl)-1,2,4-oxadiazole (**4w**)

Prepared from **2c** (0.30 g, 1.53 mmol), (E)-3-(4-chlorophenyl) acrylic acid (0.19 g, 1.25 mmol), EDCI (0.29 g, 1.51 mmol), HOBt (0.20 g, 1.48 mmol). Purification procedure: column chromatography (eluent: 33% ethyl acetate in petroleum ether with 1% TEA). White solid, yield 64%. m.p.: 157.0–158.0 °C. ^1^H NMR (400 MHz, CDCl_3_) δ 8.20–8.16 (m, 2*H*, -Ar-*H*), 7.82–7.80 (q, 1*H*, -Ar-*H*), 7.69–7.68 (d, 1*H*, -Ar-*H*), 7.08–7.05 (m, 2*H*, -Ar-*H*), 7.02–6.99 (d, 1*H*, -Ar-*H*), 4.02 (s, 3*H*, –OC*H*_3_), 3.99 (s, 3*H*, –OC*H*_3_), 3.93 (s, 3*H*, –OC*H*_3_). ^13^C NMR (101 MHz, CDCl_3_) δ 175.37, 168.57, 163.10, 151.37, 149.08, 130.07, 126.49, 120.90, 119.67, 116.88, 114.47, 113.85, 110.95, 109.84, 56.07, 55.99, 55.54. Calculate *m*/*z* for C_17_H_16_N_2_O_4_: 312.11, found: 312.19.

(*E*)-3-Phenyl-5-styryl-1,2,4-oxadiazole (**5a**)

Prepared from **2a** (0.25 g, 1.84 mmol), cinnamic acid (0.22 g, 1.21 mmol), EDCI (0.35 g, 1.83 mmol), HOBt (0.25 g, 1.85 mmol). Purification procedure: column chromatography (eluent: 14% CH_2_Cl_2_ in petroleum ether with 2% TEA). White solid, yield 40%. m.p.: 92.6–93.0 °C. ^1^H NMR (400 MHz, CDCl_3_) δ 8.17–8.14 (m, 2*H*, Ar-*H*), 7.94–7.88 (m, 1*H*, –*H*C=C*H*–), 7.66–7.63 (m, 2*H*, Ar-*H*), 7.56–7.50 (m, 3*H*, Ar-*H*) 7.49–7.44 (m, 3*H*, Ar-*H*), 7.13–7.06 (m, 1*H*, –*H*C=C*H*–). ^13^C NMR (101 MHz, CDCl_3_) δ 175.24, 175.20, 168.72, 142.73, 134.40, 131.19, 130.57, 129.10, 128.88, 127.96, 127.46, 126.93, 110.23. Calculate *m*/*z* for C_16_H_12_N_2_O: 248.09, found: 248.19.

(*E*)-3-Phenyl-5-(3,4,5-trimethoxystyryl)-1,2,4-oxadiazole (**5b**)

Prepared from **2a** (0.25 g, 1.84 mmol), (E)-3-(3,4,5-trimethoxyphenyl)acrylic acid (0.40 g, 1.68 mmol), EDCI (0.35 g, 1.83 mmol), HOBt (0.25 g, 1.85 mmol). Purification procedure: column chromatography (eluent: 17% ethyl acetate and 17% CH_2_Cl_2_ in petroleum ether with 2% TEA). Yellow solid, yield 56%. m.p.: 126.8–128.2 °C. ^1^H NMR (400 MHz, CDCl_3_) δ 8.16–8.12 (m, 2*H*, *Ar*-*H*), 7.86–7.80 (q, 1*H*, –C*H*=C*H–*), 7.55–7.51 (m, 3*H*, *Ar*-*H*), 7.04–6.98 (q, 1*H*, –C*H*=C*H–*), 6.87–6.85 (d, 2*H*, *Ar*-*H*), 3.96–3.91 (t, 9*H*, –OC*H*_3_). ^13^C NMR (101 MHz, CDCl_3_) δ 175.20, 168.67, 153.54, 142.62, 140.25, 131.20, 129.91, 128.88, 127.41, 126.90, 109.46, 105.00, 61.05, 56.18. Calculate *m*/*z* for C_19_H_18_N_2_O_4_: 338.13, found: 338.19.

(*E*)-5-(4-Chlorostyryl)-3-phenyl-1,2,4-oxadiazole (**5c**)

Prepared from **2a** (0.20 g, 1.47 mmol), (E)-3-(4-chlorophenyl)acrylic acid (0.23 g, 1.26 mmol), EDCI (28 g, 1.46 mmol), HOBt (0.20 g, 1.47 mmol). Purification procedure: column chromatography (eluent: 25% ethyl acetate and 25% CH_2_Cl_2_ in petroleum ether). Yellow solid, yield 57%. m.p.: 162.5–164.0 °C. ^1^H NMR (400 MHz, CDCl_3_) δ 8.17–8.14 (m, 2*H*, Ar-*H*), 7.89–7.85 (d, 1*H*, –C*H*=C*H*–), 7.59–7.51 (m, 5*H*, Ar-*H*), 7.45–7.43 (d, 2*H*, Ar-*H*), 7.09–7.05 (d, 1*H*, –C*H*–C*H*–). ^13^C NMR (101 MHz, DMSO-*d*_6_) δ 175.74, 168.51, 141.94, 135.65, 133.70, 132.08, 130.59, 129.75, 129.53, 127.49, 126.69, 111.49. Calculate *m*/*z* for C_16_H_11_ClN_2_O: 282.06, found: 282.09.

(*E*)-3-(4-Methoxyphenyl)-5-styryl-1,2,4-oxadiazole (**5d**)

Prepared from **2b** (0.28 g, 1.69 mmol), cinnamic acid (0.21 g, 1.42 mmol), EDCI (0.32 g, 1.67 mmol), HOBt (0.23 g, 1.7 mmol). Purification procedure: column chromatography (eluent: 67% CH_2_Cl_2_ in petroleum ether with 1% TEA). Light yellow solid, yield 38%. m.p.: 124.2–125.5 °C. ^1^H NMR (400 MHz, CDCl_3_) δ 8.12–8.07 (m, 2*H*, *Ar*-*H*), 7.92–7.88 (d, 1*H*, –*HC=CH*–), 7.65–7.63 (m, 2*H*, *Ar*-*H*), 7.50–7.44 (m, 3*H*, *Ar*-*H*), 7.11–7.07 (d, 1*H*, –*HC=CH*–), 7.05–7.02 (d, 2*H*, *Ar*-*H*), 3.90 (s, 3*H*, –OC*H*_3_). ^13^C NMR (101 MHz, DMSO-*d*_6_) δ 175.54, 168.17, 162.15, 143.08, 134.73, 131.07, 129.45, 129.16, 128.83, 119.01, 115.09, 110.75, 55.85. Calculate *m*/*z* for C_17_H_14_N_2_O_2_: 278.11, found: 278.19.

(*E*)-3-(3,4-Dimethoxyphenyl)-5-styryl-1,2,4-oxadiazole (**5e**)

Prepared from **2c** (0.30 g, 1.53 mmol), cinnamic acid (0.19 g, 1.28 mmol), EDCI (0.28 g, 1.46 mmol), HOBt (0.20 g, 1.47 mmol). Purification procedure: column chromatography (eluent: 25% ethyl acetate and 25% CH_2_Cl_2_ in petroleum ether). Light yellow solid, yield 51%. m.p.: 115.4–116.5 °C. ^1^H NMR (400 MHz, CDCl_3_) δ 7.93–7.89 (d, 1*H*, –*HC=CH*–), 7.78–7.76 (q, 1*H*, *Ar*-*H*), 7.65–7.63 (m, 3*H*, *Ar*-*H*), 7.48–7.45 (m, 3*H*, *Ar*-*H*), 7.11–7.07 (d, 1*H*, –*HC=CH*–), 7.01–6.99 (d, 1*H*, -*Ar*-*H*), 4.01 (s, 3*H*, –OC*H*_3_), 3.98(s, 3*H*, –OC*H*_3_). ^13^C NMR (101 MHz, CDCl_3_) δ 175.00, 168.47, 151.43, 149.11, 142.60, 134.41, 130.53, 129.08, 127.92, 120.84, 119.46, 110.98, 110.23, 109.76, 56.06, 55.98. Calculate *m*/*z* for C_18_H_16_N_2_O_3_: 308.12, found: 308.19.

### 3.2. Cell Culture and Cytotoxicity Evaluation

The MCF-7 cell lines were grown in the RPMI 1640 medium (Gibco, Billings, MT, USA) supplemented with 10% fetal bovine serum (Gibco, USA), 100 μg/mL penicillin, and 100 μg/mL streptomycin. Cells were incubated at 37 °C in an incubator with 5% CO_2_.

Cell viability was determined using the MTT assay. Briefly, MCF-7 cell lines were seeded at 5000–8000 cells/well in 96-well plates. In this study, different groups contained cells suspended with various compound concentrations (0–100 μM), and three replicated wells were put up for each group. The control cells received 1% DMSO. Following a continuous 24 h of incubation, the culture medium was replaced by 90 μL fresh culture medium, and 10 μL MTT solution was added. After incubating for 4 h at 37 °C, the medium containing MTT was removed from the plate, and 150 mL DMSO was added to each well and shaken for 10 min; then, the results were recorded using a microplate reader to measure the absorbance of the wells at 490 nm.

### 3.3. Fungicidal Activity Testing

#### 3.3.1. Plant Pathogens

Plant pathogens *R. solani*, *F. graminearum*, and *E. turcicum* were provided by the school of plant protection of Southwest University. After the plant pathogen was removed from the storage tube, the strain was cultured in PDA solid medium at 25 °C for one week to obtain new mycelium and conduct the antifungal test.

#### 3.3.2. In Vitro Antifungal Assay

The antifungal activity of the target compound was tested by the mycelial growth inhibition rate method. The compounds were detected at a concentration of 50 μg/mL, and carbendazim was used as the positive control. The antifungal activity was carried out according to literature reports [38,40]. After drilling the mycelium (3 mm), it was inoculated into the PDA medium containing compounds, and the Petri dish was put into the incubator at 25 °C for 12–48 h; then, the colony diameter was measured with 50 index vernier caliper by the cross method, and the data were recorded.

The inhibition rate of the compound against the growth of the strain was calculated according to Formula (1), expressed as the average inhibition rate ± standard deviation (S.D.).

(1) $$Inhibition\;rate\left(\%\right)=\frac{\left[\left(A-B\right)-(C-B)\right]}{\left(A-B\right)}\times 100\%$$

- A: The average diameter of the blank medium colony.
- B: Diameter of mushroom cake (3 mm).
- C: The average diameter of the colonies in the medium containing compound.

#### 3.3.3. Determination of EC_50_

According to the results of preliminary screening of antifungal activity, the derivatives with mycelial growth inhibition rates greater than 40% were selected, and the *EC*_50_ value was determined. The PDA medium containing different concentrations of the compound was prepared by the two-fold dilution method, the final concentration of the compound in the medium was adjusted to 0–100.00 μg/mL, and three parallel samples were set for each concentration. The inhibition rate of the compound against different plant pathogens was calculated according to Formula (1). The toxicity regression equation of the tested compound was obtained, taking the logarithm of compound concentration (lg*c*) as the independent variable (*x*) and the probability value of inhibition rate as the dependent variable (*y*), and the *EC*_50_ value of each tested compound was calculated.

#### 3.3.4. Study on the State of Fungi

*E. turcicum* was observed using scanning electron microscopy (SEM) [41,42]. Then, 1.5 mL *E. turcicum* in the PDB medium was incubated to the logarithmic stage, washed three times by centrifugation (7000 rpm) with 1mL PBS (pH = 7.2), and then suspended in 1.5 mL PBS. Compounds dissolved in DMSO were added, and an equal volume of the DMSO solution was added to the control group and incubated in a shaker with 180 rpm at 30 °C for 24~48 h. After incubation, the hyphaes were washed by centrifugation with PBS (pH = 7.2) for three times. Then, 1 mL 2.5% glutaraldehyde fixative was added to fix the hyphaes overnight at 4 °C. After removing the fixator, the mycelia were dehydrated with a graded ethanol series and dispersed in tert-butanol. After freeze-drying, they were observed using a scanning electron microscope (TESCAN, MIRA4, Brno, Czech Republic) at an accelerated voltage of 10 keV.

#### 3.3.5. Determination of the Antifungal Activity In Vivo

The 0.1% tween-80 aqueous solution was used as a blank control, and carbendazim was used as a positive control.

The peppers were washed with water and dried, and the surface was wiped with 75% ethanol for sterilization. The infected area was pricked with a diameter of 4 mm on the surface of the green pepper with a sterile inoculation needle. After spraying the antifungus compound solution on the surface and resting for 12 h, the activated cake (4 mm in diameter) was inoculated into the infected area. After culturing at 25 °C for 1 to 7 days, the plaque diameter was measured by the cross method, and the efficacy was calculated according to Formula (2).

(2) $$Control\;efficacy\left(\%\right)=\frac{A-B}{A-C}\times 100\%$$

- A: The average diameter of the blank medium colony.
- B: The average diameter of the colonies in the medium containing compound.
- C: Diameter of mushroom cake (3 mm/4 mm).

### 3.4. Molecular Docking

Molecular docking was performed with Surflex-Dock implemented in SYBYL (version 2.1.1). The crystal structure of SDH (PDB code 2fbw) was downloaded from the RCSB Protein Data Bank (http://www.rcsb.org/, accessed on 18 April 2025), and the protein structure with Sybyl was prepared to remove the bound water and ligands in the protein.

A compound structure database was established in Sybyl, each small molecule was hydrogenated and charged (Gasteiger-Hückel), and the Tripos force field was selected for energy optimization (optimization times 10,000, energy convergence standard 0.005 kcal/mol·A). Then, the related forms were created.

The compound structure database is established in Sybyl, each small molecule is hydrogenated and charged (Gasteiger-Hückel), and the Tripos force field is selected for energy optimization with optimization times of 10,000 and energy convergence standard of 0.005 kcal/mol·A. The molecular docking study (standard mode) was completed by using the Surflex-Dock module. The ligand mode was selected to generate the active pocket, and other parameters adopted the default values of SYBYL. Ligplot (version 2.2.4) and Pymol (version 2.5) were used to analyze the interaction between the inhibitor and SDH.

## 4. Conclusions

1,2,4-oxadiazole derivatives for SDH inhibitors were synthesized by the multi-step reaction from substituted benzaldehyde and anisic acid or cinnamic acid derivatives. In the preliminary screening of biological activity, it was found that the target compounds of anisic acid derivatives usually showed more excellent activity and a wider antifungal spectrum. The activity of unsubstituted benzaldehyde is higher than that of mono-substitution, and that of mono-substitution is higher than that of multi substitution. In addition, the target compounds containing phenolic hydroxyl groups, especially at positions 3 and 4 of the aromatic ring, have higher antifungal activity than other substituents. At the same time, some target molecules have excellent antifungal activity, which is highlighted by compounds **4f** and **4q**. Among them, **4f** has the best growth inhibition rate of 100% against *F. graminearum* at a concentration of 50 μg/mL. In particular, the inhibitory effect of **4f** against *E. turcicum* was significantly better than that of carbendazim. Compounds **4f** and **4q** showed antifungal effects on *C. capsica* of capsicum in vivo. Computer simulation of molecular docking shows that **4f** and **4q** interact with SDH through hydrogen bonds and hydrophobic interactions. Compound **4q** can form hydrogen bonds with TRP173 and ILE27 of SDH, and **4f** has hydrogen bonds with TYR58, TRP173, and SER39. We will further study the structural optimization of antifungal agents and the interaction relationship between key compounds and targets in order to obtain new SDH inhibitors to improve antifungal activity and find out the mode of action.

## Figures and Tables

**Table 1:** The inhibition rate (%) of 1,2,4-oxadiazole derivatives against phytopathogens at 50 μg/mL.

Compound	The Inhibition Rate (%)				
	*R. solani*	*F. graminearum*	*E. turcicum*	*B. cinerea*	*C. capsica*
**4a**	<10	19.06 ± 7.15	17.54 ± 2.68	<10	-
**4b**	18.68 ± 5.82	23.75 ± 7.22	60.39 ± 1.68	<10	<10
**4c**	10.74 ± 3.54	33.47 ± 3.88	30.07 ± 1.95	<10	<10
**4d**	18.60 ± 2.71	40.22 ± 1.50	46.11 ± 0.74	36.77 ± 9.53	22.28 ± 2.97
**4e**	16.61 ± 1.47	30.07 ± 4.51	26.47 ± 3.09	<10	18.84 ± 3.10
**4f**	80.87 ± 2.40	100	85.71 ± 1.97	38.85 ± 5.35	86.85 ± 3.11
**4g**	39.91 ± 3.44	56.86 ± 6.74	38.67 ± 1.00	<10	47.65 ± 1.05
**4h**	<10	28.98 ± 8.54	<10	<10	<10
**4i**	<10	<10	<10	<10	-
**4j**	<10	<10	<10	<10	<10
**4k**	22.59 ± 8.85	21.67 ± 2.53	<10	<10	<10
**4l**	<10	27.58 ± 1.78	24.16 ± 3.77	<10	<10
**4m**	11.73 ± 5.11	<10	15.16 ± 0.77	-	-
**4n**	<10	<10	29.79 ± 1.26	<10	<10
**4o**	24.43 ± 1.65	55.10 ± 2.99	28.09 ± 1.65	<10	20.82 ± 0.59
**4p**	<10	<10	11.25 ± 1.91	<10	0
**4q**	47.76 ± 8.13	43.48 ± 3.70	43.54 ± 0.99	32.84 ± 12.16	55.54 ± 1.34
**4s**	<10	<10	<10	<10	<10
**4t**	<10	28.27 ± 2.19	<10	<10	-
**4u**	<10	<10	<10	<10	<10
**4v**	<10	26.07 ± 9.47	15.46 ± 3.53	<10	<10
**4w**	<10	<10	<10	<10	<10
**5a**	14.52 ± 6.32	<10	21.15 ± 3.85	<10	-
**5b**	21.17 ± 2.78	36.09 ± 1.67	31.81 ± 1.44	30.16 ± 6.40	20.62 ± 4.48
**5c**	<10	<10	<10	<10	-
**5d**	<10	19.02 ± 7.40	19.22 ± 2.39	21.71 ± 6.82	-
**5e**	23.89 ± 0.59	28.74 ± 5.14	29.42 ± 2.05	30.56 ± 1.43	<10

Values are the mean ± standard deviation (SD) of three replicates. “-” means no experiment is carried out. “<10”means that the inhibition rate is lower than 10%, or in the case where the inhibition rate is lower than 10% after deducting the error value.

**Table 2:** The *EC*_50_ (μg/mL) values of part compounds against plant-pathogenic fungi.

Plant Pathogen	Compound	The Regression Equation	*R* ^2^	*EC*_50_ (μg/mL)	95% CI (μg/mL)
*R. solani*	**4q**	*y* = 2.077*x* + 1.697	0.9163	38.88	24.62–164.03
	**4f**	*y* = 4.073*x* + 0.507	0.8752	12.68	6.02–26.74
	carbendazim	*y* = 1.790*x* + 4.924	0.9265	1.10	0.68–1.78
*F. graminearum*	**4q**	*y* = 0.969*x* + 2.894	0.9479	149.26	51.84–429.78
	**4g**	*y* = 2.179*x* + 0.848	0.9496	80.55	45.54–142.46
	**4f**	*y* = 0.992*x* + 3.536	0.9335	29.97	19.44–46.22
	carbendazim	*y* = 0.926*x* + 3.804	0.9991	19.59	18.34–20.91
*E. turcicum*	**4q**	*y* = 1.025*x* + 2.580	0.9375	228.99	82.79–633.38
	**4f**	*y* = 1.412*x* + 2.933	0.9893	29.14	24.70–34.39
	carbendazim	*y* = 2.246*x* + 0.419	0.9643	109.56	65.03–184.58
*C. capsica*	**4q**	*y* = 1.596*x* + 2.414	0.9947	41.67	36.61–47.44
	**4f**	*y* = 1.184*x* + 3.882	0.9845	8.81	6.60–11.76
	carbendazim	*y* = 2.004*x* + 6.307	0.9296	0.22	0.12–0.43

**Table 3:** Efficacy in controlling *C. capsica* strains on detached pepper by **4q** and **4f**.

Treatment	Blank	Carbendazim (5 Days)		4f		4q (5 Day)	
Concentration (μg/mL)	-	500	1000	500 (5 day)	1000 (3 day)	500	1000
Control efficacy (%)	0	100	100	100	100	50.12	76.83

## Data Availability

The original contributions presented in this study are included in the article/Supplementary Material. Further inquiries can be directed to the corresponding author.

## Supplementary Materials

The following supporting information can be downloaded at: https://www.mdpi.com/article/10.3390/molecules30081851/s1. Table S1. Structures of target compounds; Figure S1. ^1^HNMR of **3a**–**3e**; Figure S2. ^1^HNMR and ^13^CNMR of **4a**–**4w**; Figure S3. ^1^HNMR and ^13^CNMR of **5a**–**5f**; Figure S4. Mass spectra; Figure S5. Three-dimensional docking of carbendazim with the active pocket of protein 2fbw (A). Two-dimensional ligand hydrophobic interaction (B) of carbendazim with amino acid residues. Hydrogen bond interaction of carbendazim with amino acid residues (C); Table S2. The primary data for Table 1 (*R. solani*); Table S3. The primary data for Table 1 (*F. graminearum*); Table S4. The primary data for Table 1 (*E. turcicum*); Table S5. The primary data for Table 1 (*B. cinerea*); Table S6. The primary data for Table 1 (*C. capsica*).
