# Supplementary material for: Synthesis and Antifungal Activity of 1,2,4-Oxadiazole Derivatives
Source: Molecules. 2025 Apr 20;30(8):1851. doi: 10.3390/molecules30081851 (PMC12029309; doi:10.3390/molecules30081851)
Supplement: Supplementary file 1 [file molecules-30-01851-s001.zip › molecules-3565403-supplementary.pdf]

Synthesis and Antifungal Activity of 1,2,4-Oxazole Derivatives

Table S1. Structures of Target Compounds

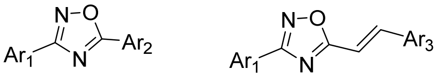

4a-4w **5a-5e**

Compound	Product	Ar1	Ar2	Ar3
**4a**	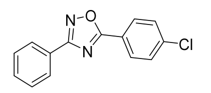	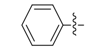	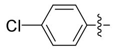	-
**4b**	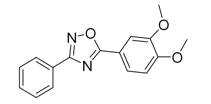	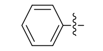	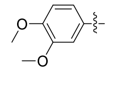	-
**4c**	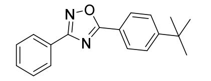	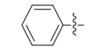	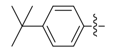	-
**4d**	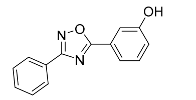	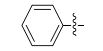	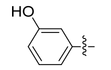	-
**4e**	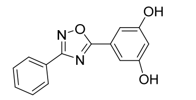	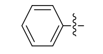	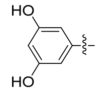	-
**4f**	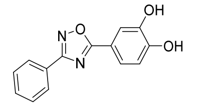	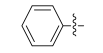	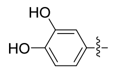	-
**4g**	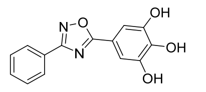	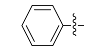	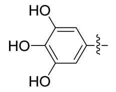	-
**4h**	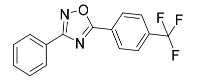	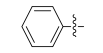	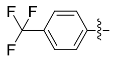	-
**4i**	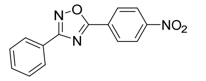	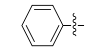	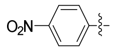	-
**4j**	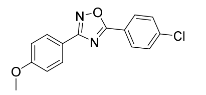	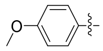	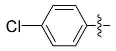	-
**4k**	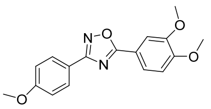	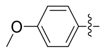	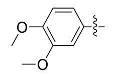	-
**4l**	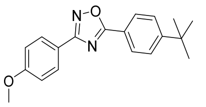	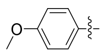	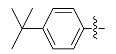	-
**4m**	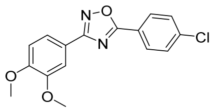	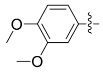	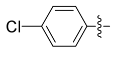	-

**4n**	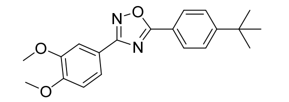	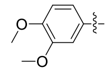	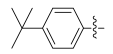	-
**4o**	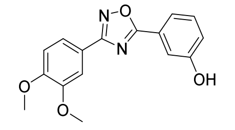	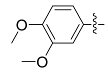	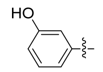	-
**4p**	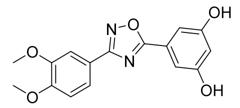	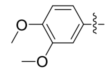	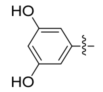	-
**4q**	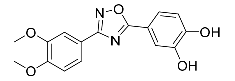	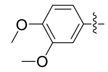	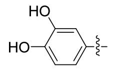	-
**4s**	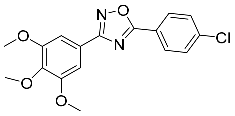	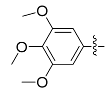	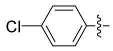	-
**4t**	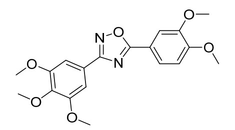	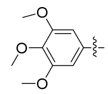	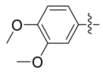	-
**4u**	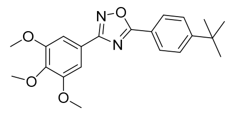	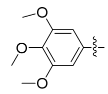	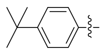	-
**4v**	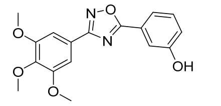	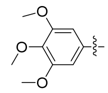	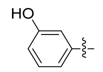	-
**4w**	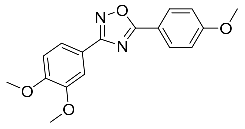	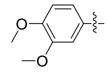	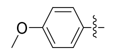	-
**5a**	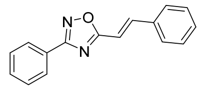	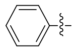	-	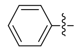
**5b**	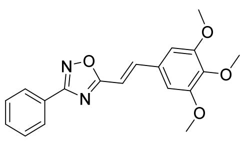	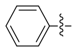	-	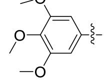
**5c**	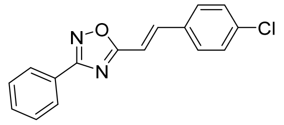	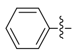	-	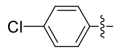
**5d**	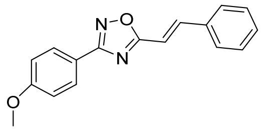	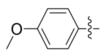	-	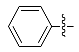
**5e**	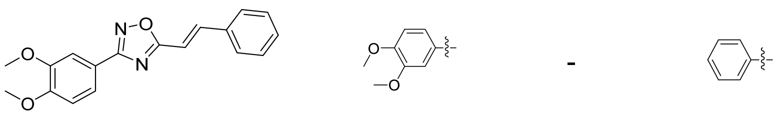		-	

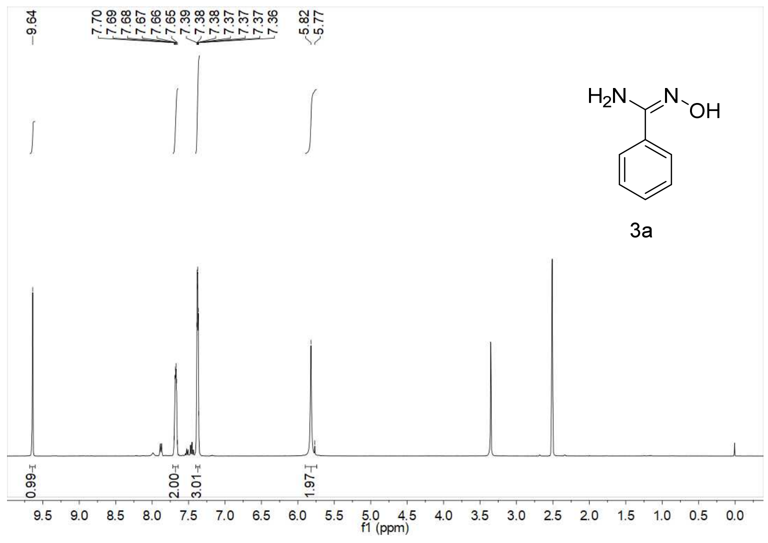

^{1}H NMR (400 MHz, DMSO-d6)

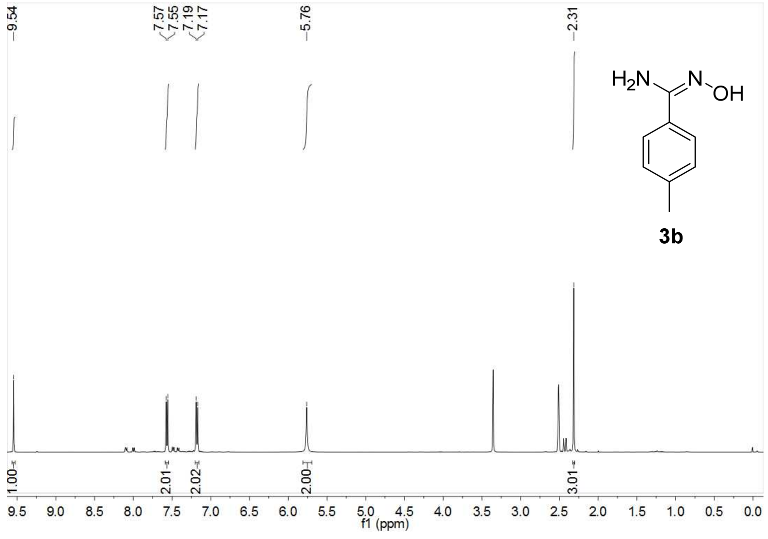

^{1}H NMR (400 MHz, DMSO)

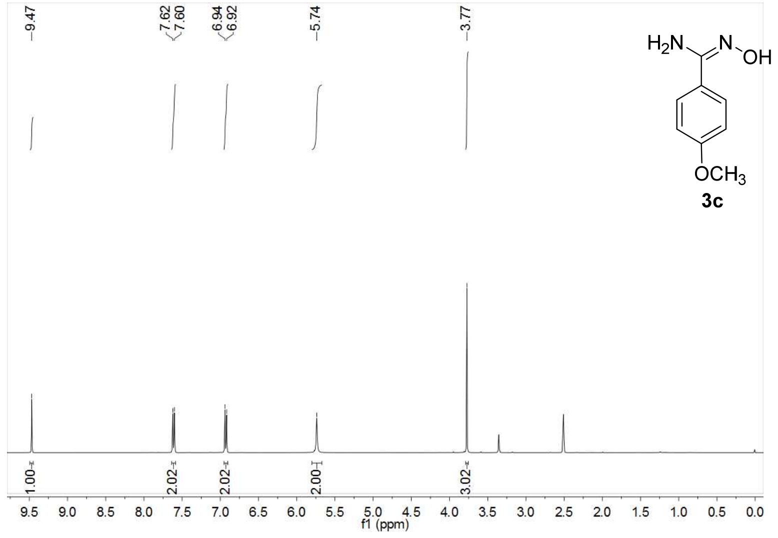

^{1}H NMR (400 MHz, DMSO-d6)

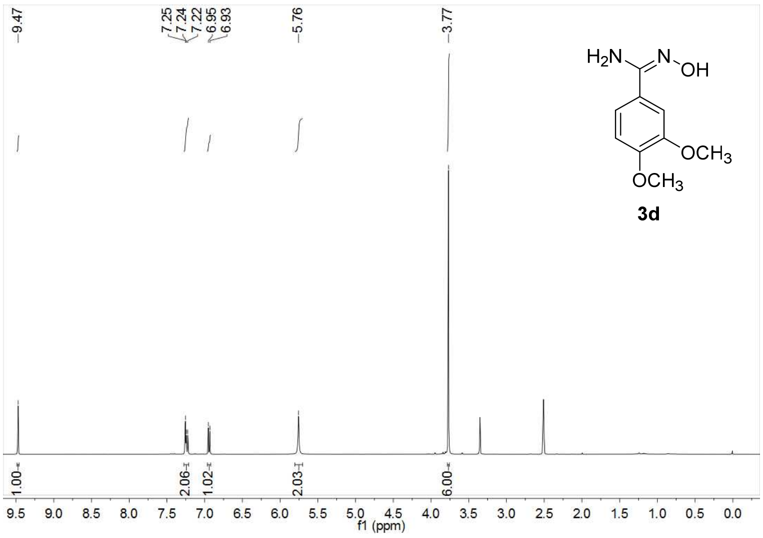

^{1}H NMR (400 MHz, DMSO-d6)

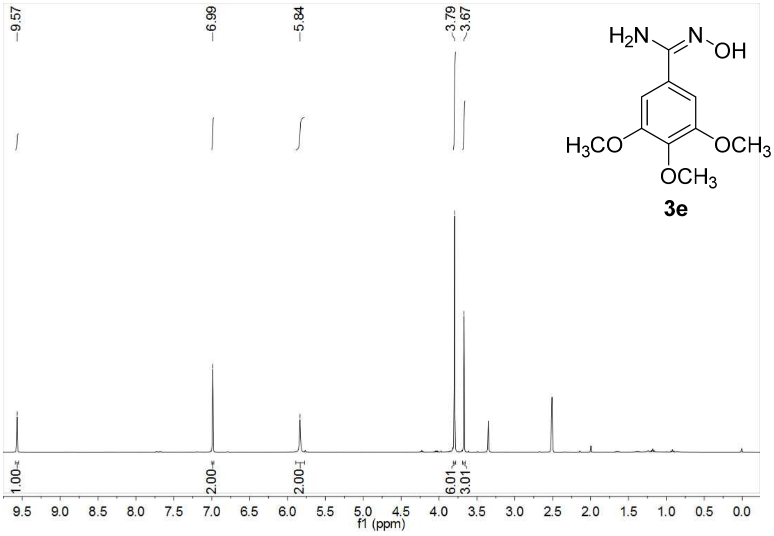

^{1}H NMR (400 MHz, DMSO-d6)

Figure S1. ^{1}HNMR of 3a-3e

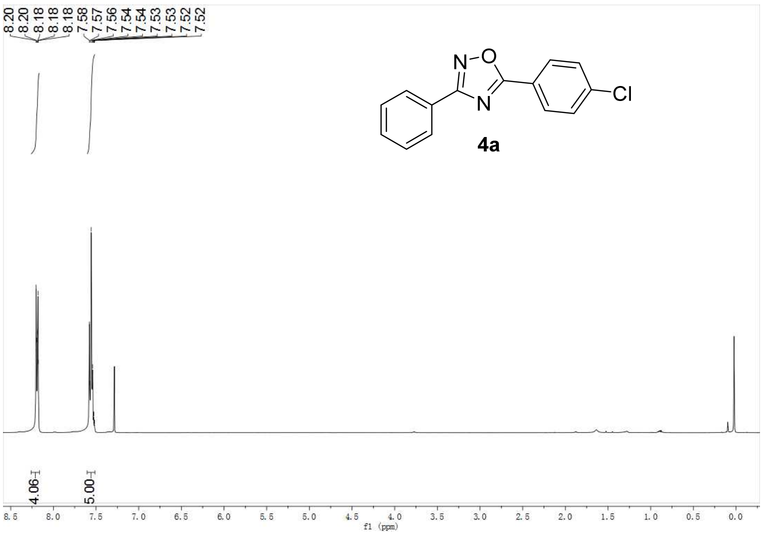

^{1}H NMR (400 MHz, $CDCl_3$)

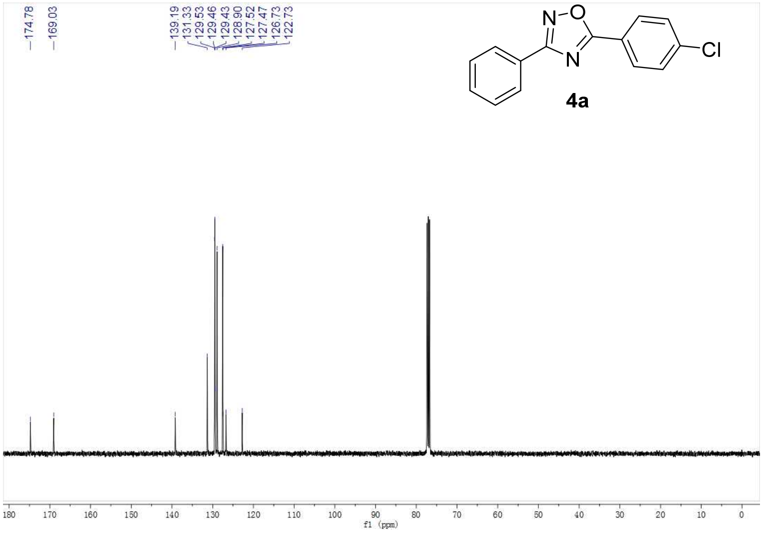

^{13}C NMR (101 MHz, $CDCl_3$)

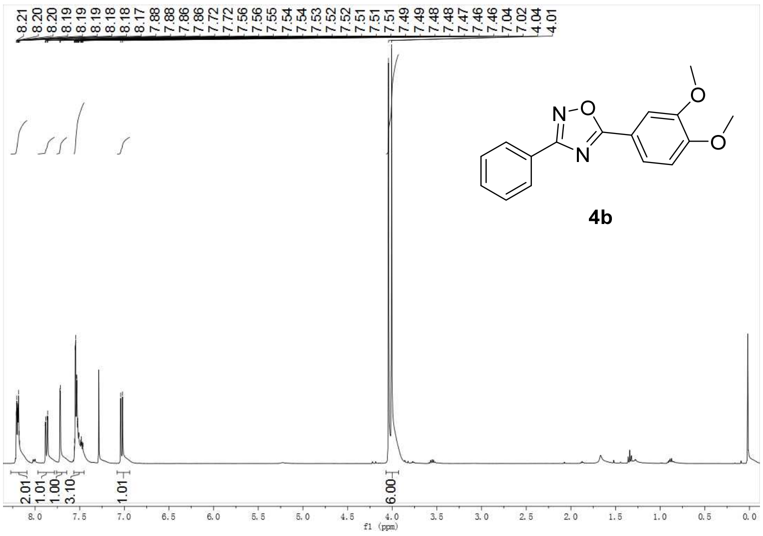

^{1}H NMR (400 MHz, $CDCl_3$)

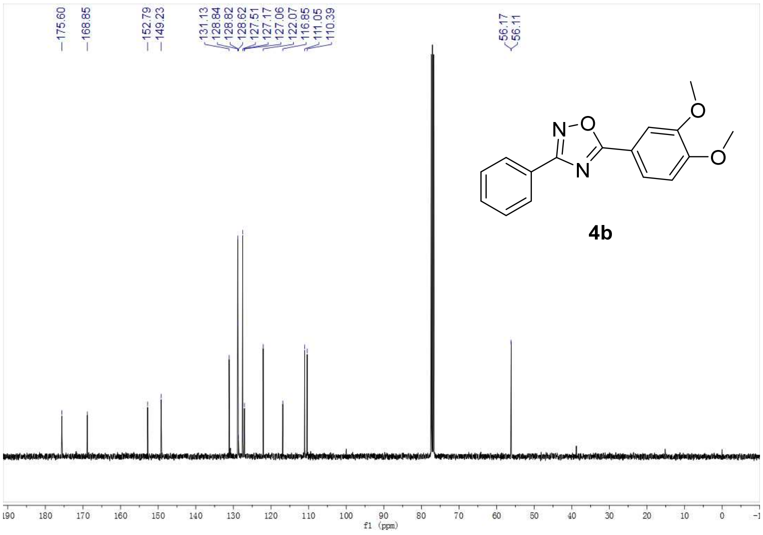

^{13}C NMR (101 MHz, $CDCl_3$)

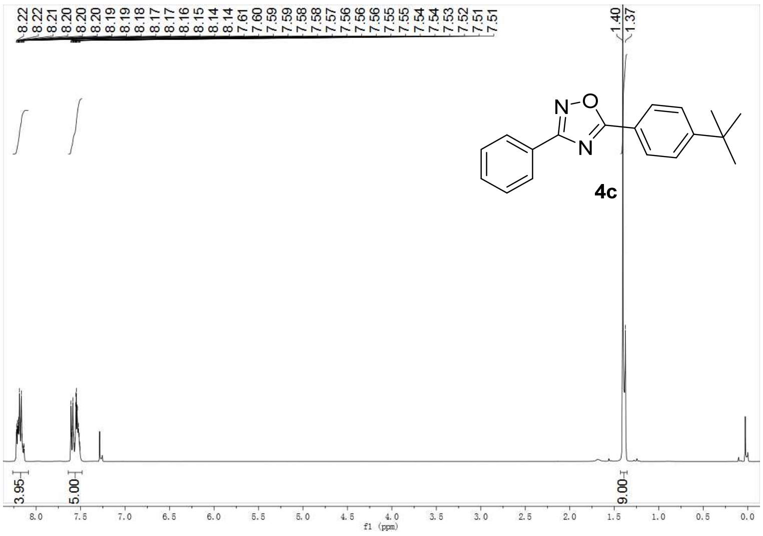

^{1}H NMR (400 M*Hz*, $CDCl_3$)

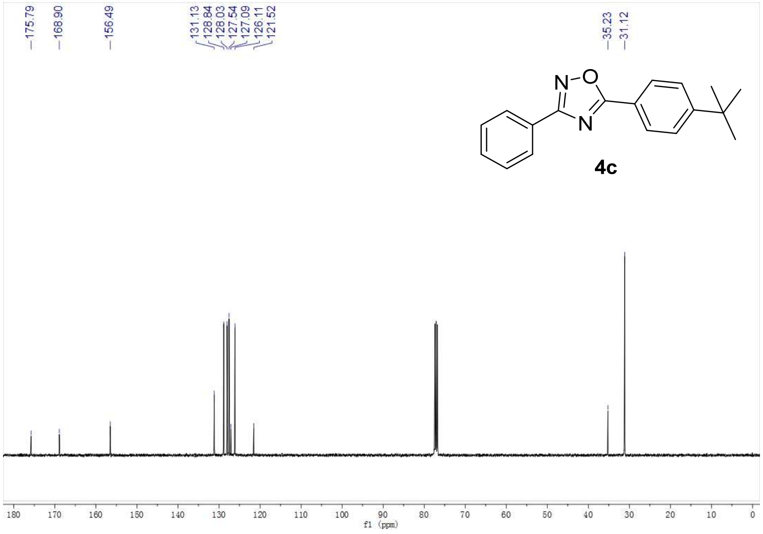

^{13}C NMR (101 MHz, $CDCl_3$)

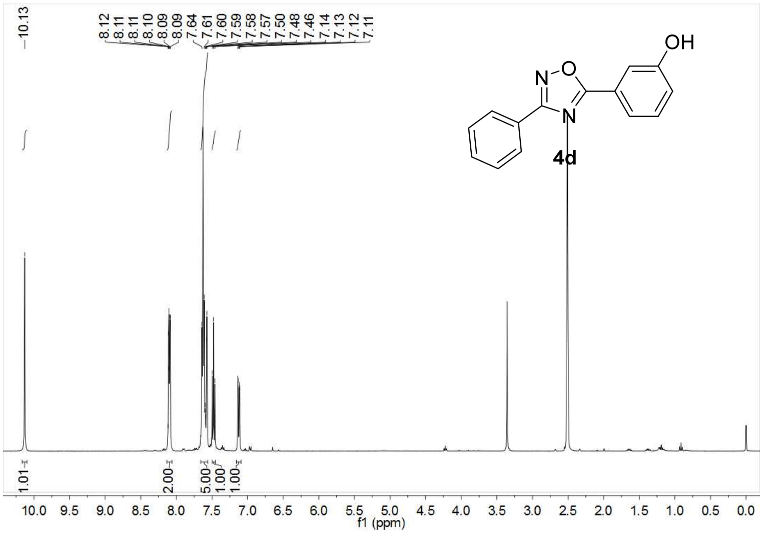

^{1}H NMR (400 MHz, DMSO-d6)

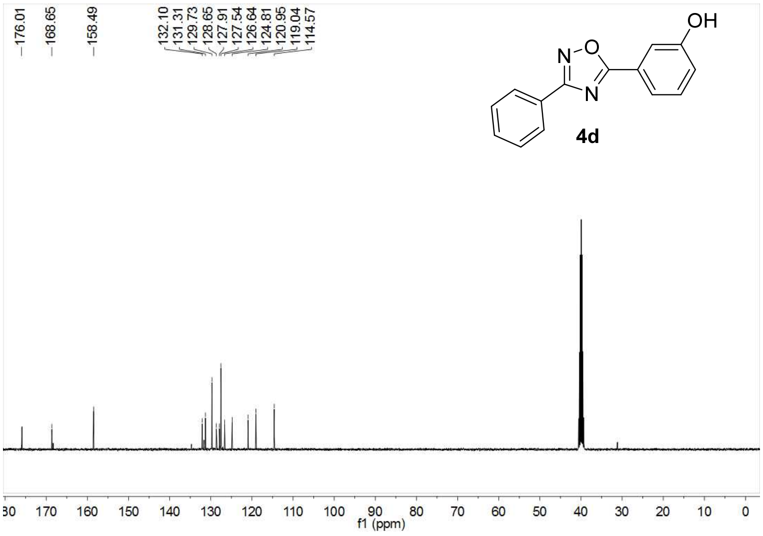

^{13}C NMR (101 MHz, DMSO-d6)

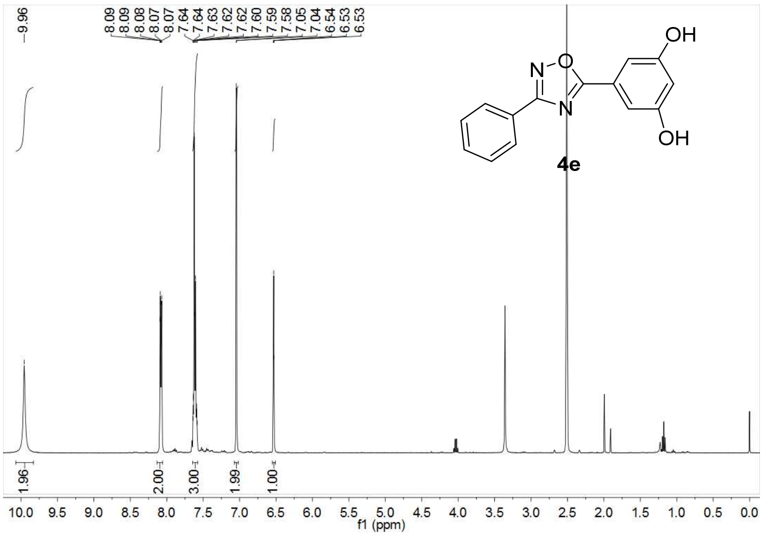

^{1}H NMR (400 MHz, DMSO-d6)

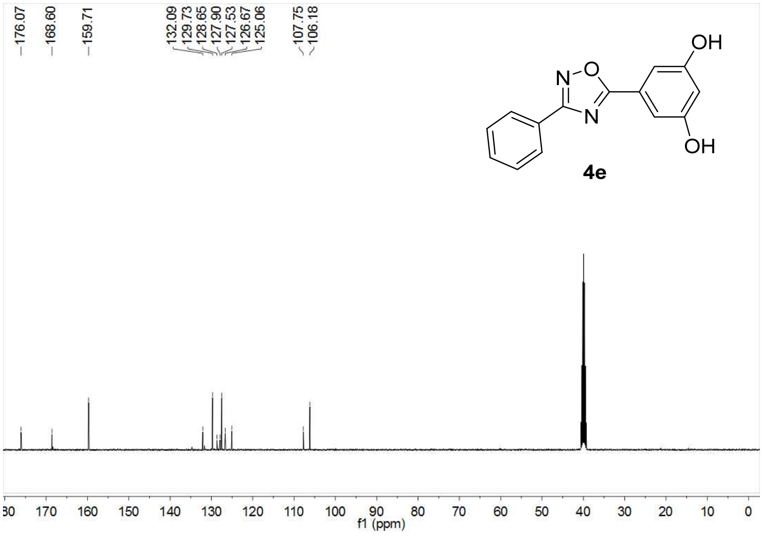

^{13}C NMR (101 MHz, DMSO-d6)

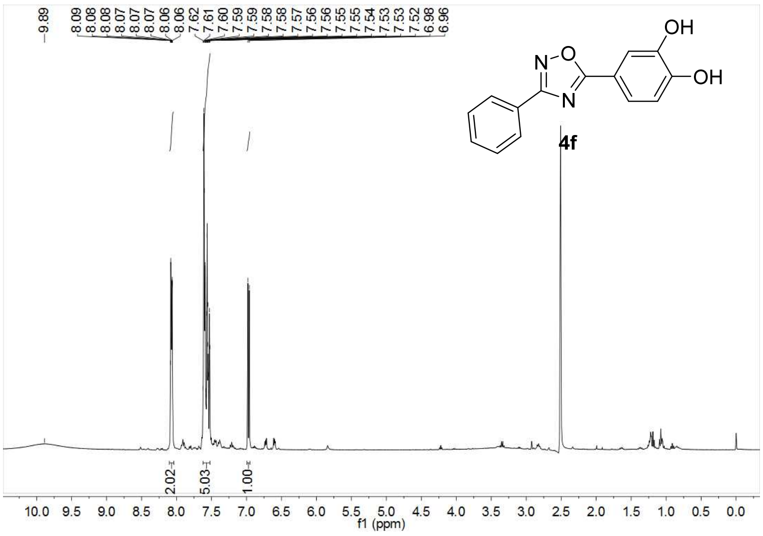

^{1}H NMR (400 MHz, DMSO-d6)

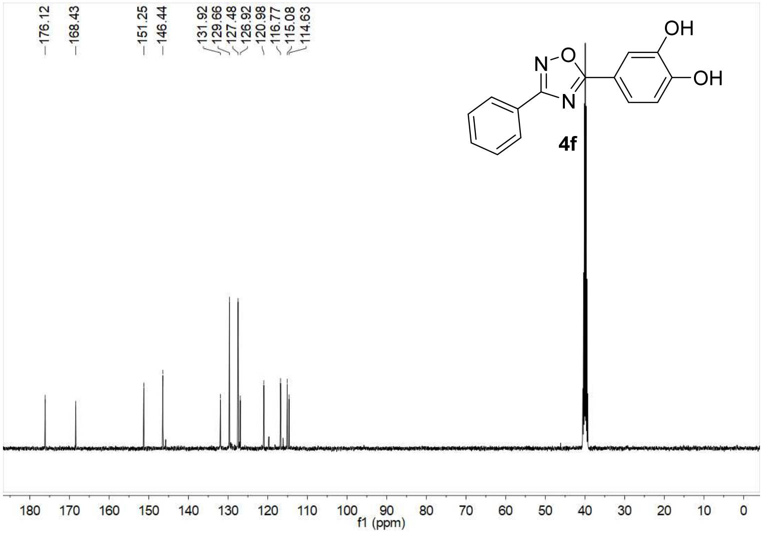

^{13}C NMR (101 MHz, DMSO-d6)

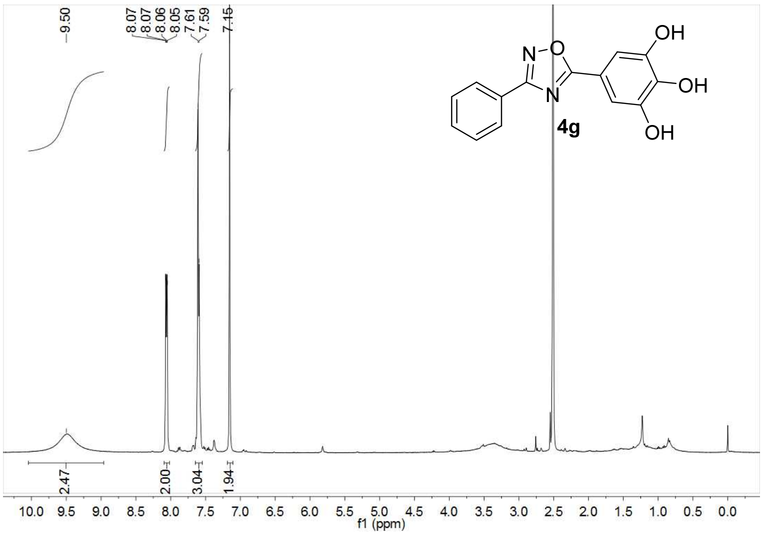

H NMR (400 MHz, DMSO-d6)

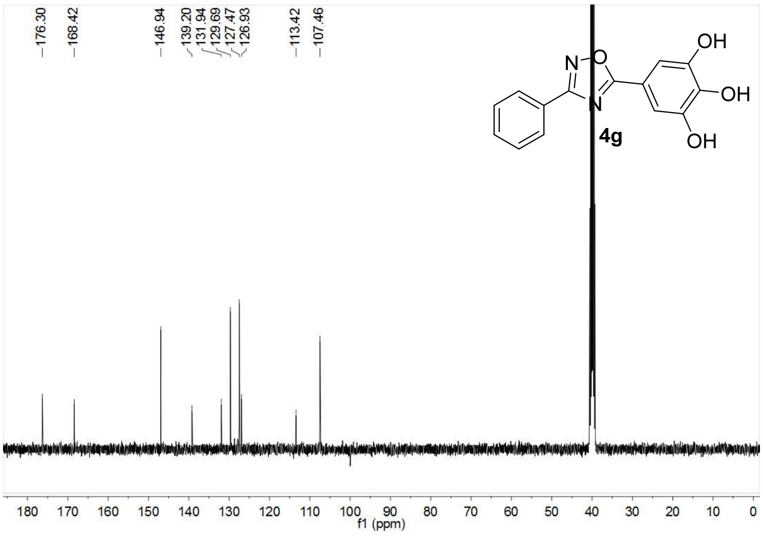

^{13}C NMR (101 MHz, DMSO-d6)

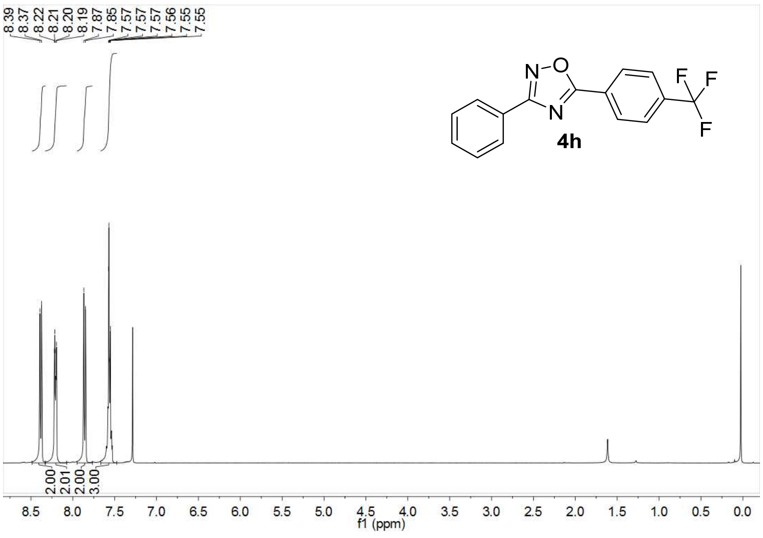

^{1}H NMR (400 MHz, $CDCl_3$)

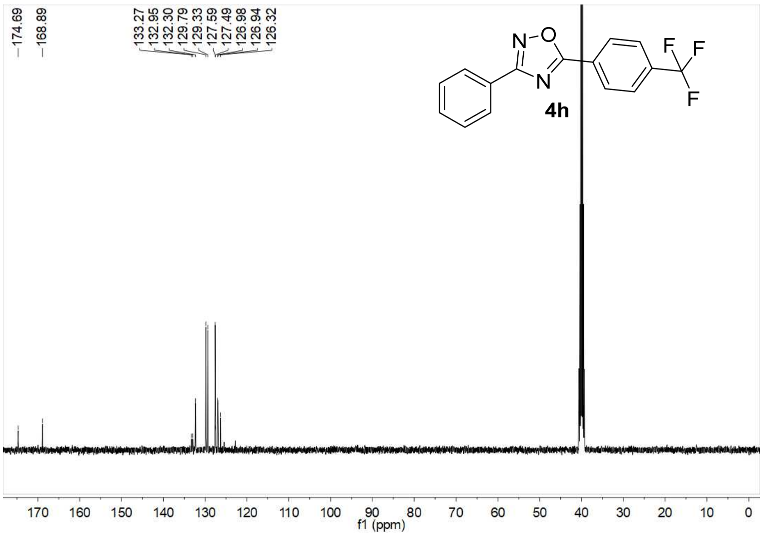

^{13}C NMR (101 MHz, DMSO-d6)

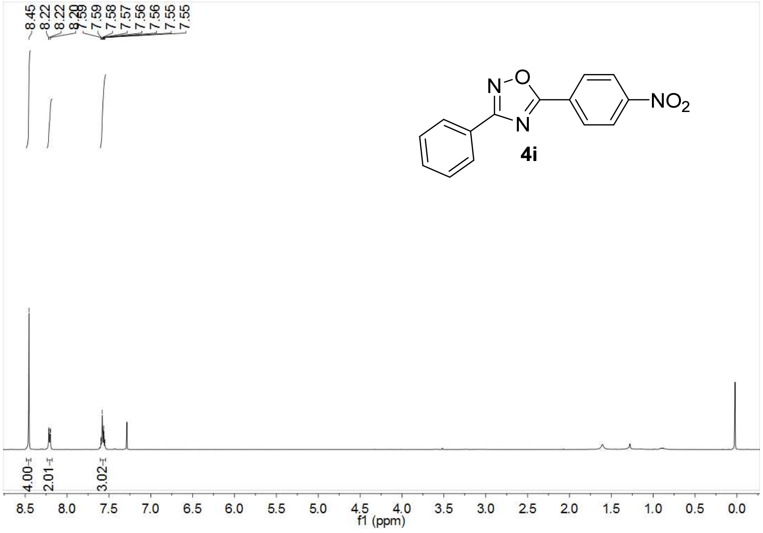

^{1}H NMR (400 MHz, $CDCl_3$)

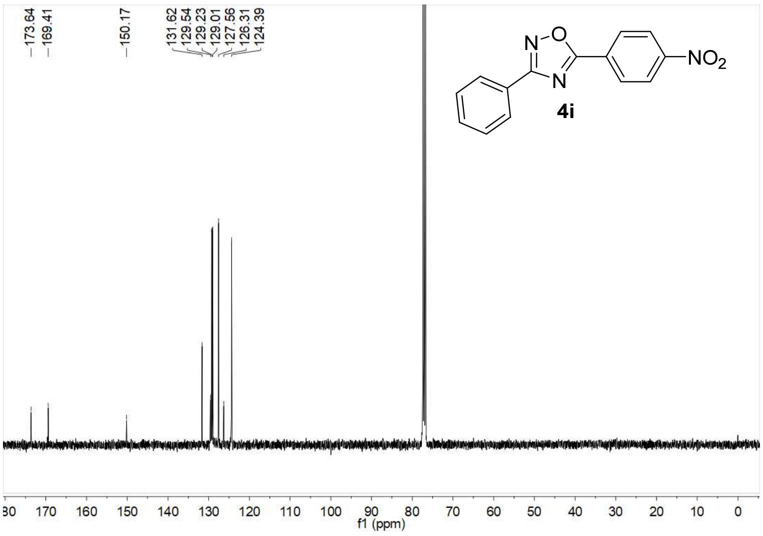

^{13}C NMR (101 MHz, $CDCl_3$)

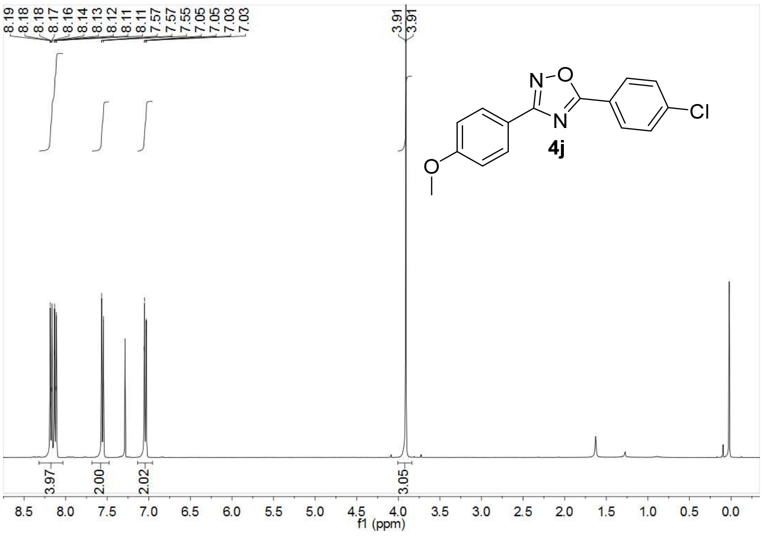

^{1}H NMR (400 MHz, $CDCl_3$)

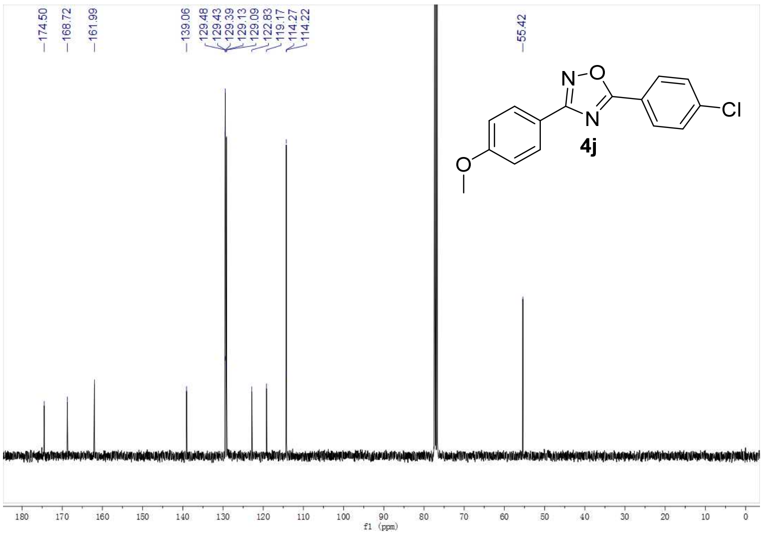

^{13}C NMR (101 MHz, $CDCl_3$)

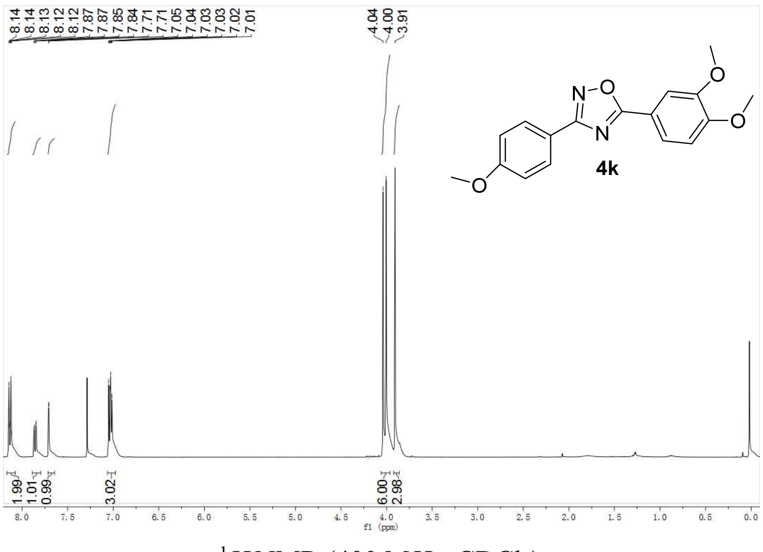

^{1}H NMR (400 MHz, $CDCl_3$)

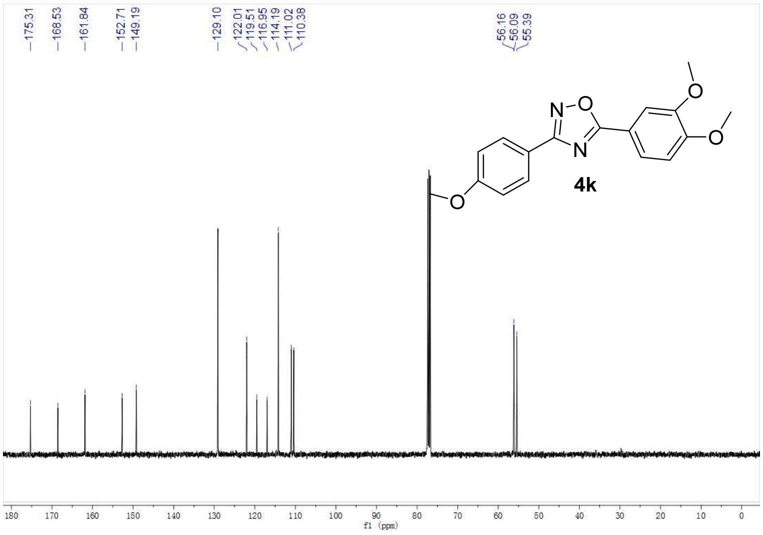

^{13}C NMR (101 MHz, $CDCl_3$)

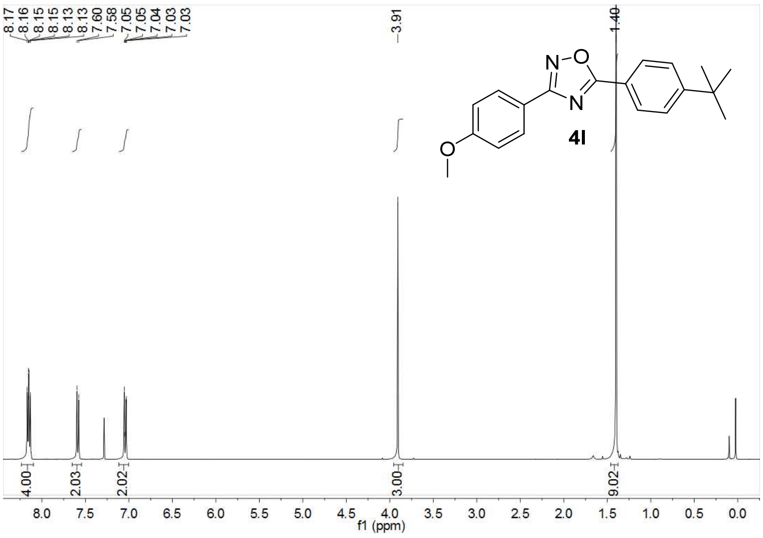

^{1}H NMR (400 MHz, $CDCl_3$)

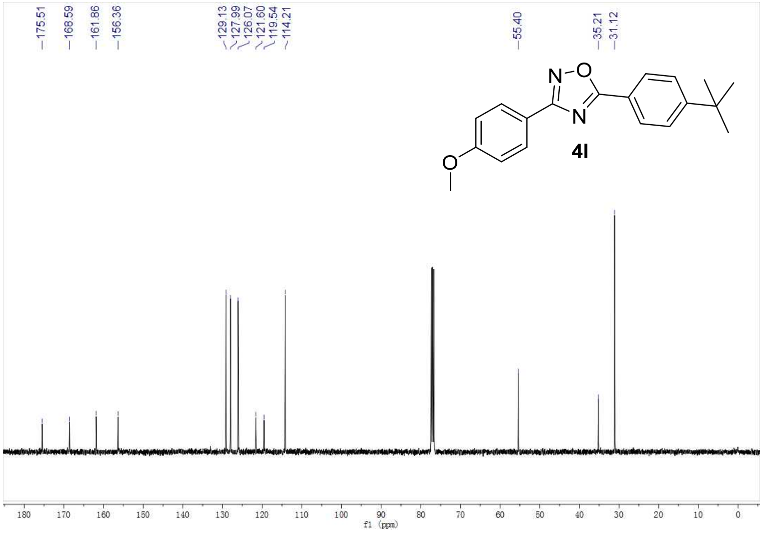

^{13}C NMR (101 MHz, $CDCl_3$)

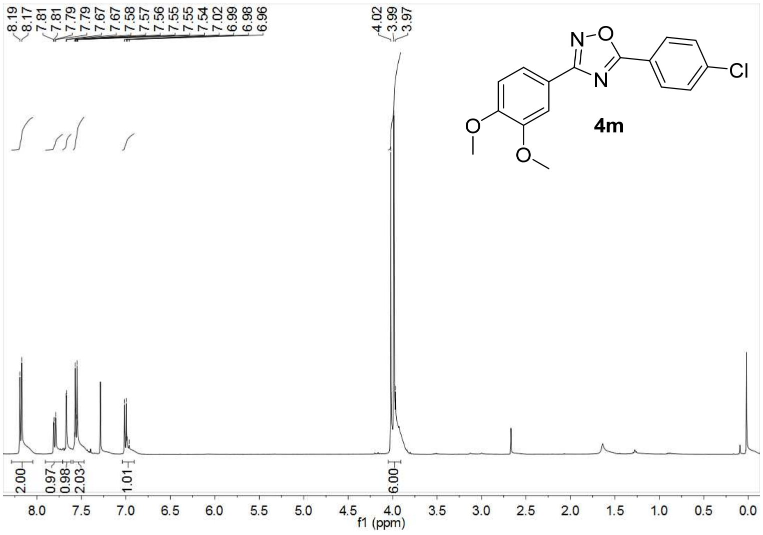

[1]*H* NMR (400 M*Hz*, $CDCl_3$)

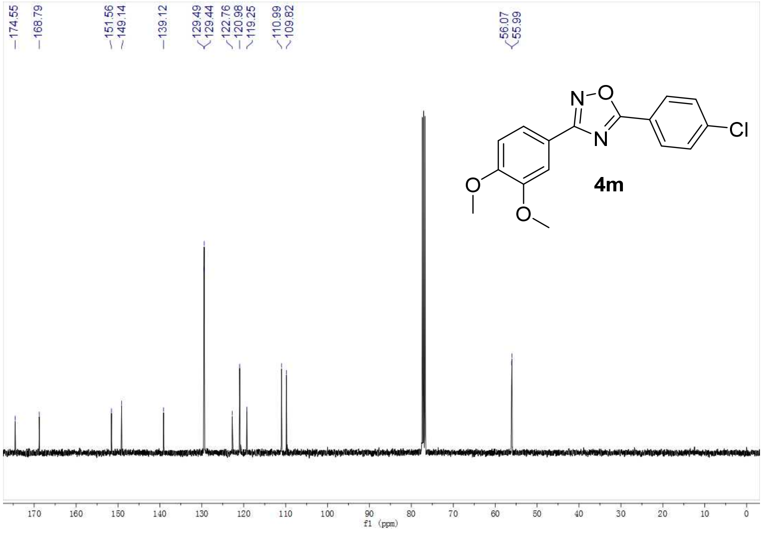

^{13}C NMR (101 MHz, $CDCl_3$)

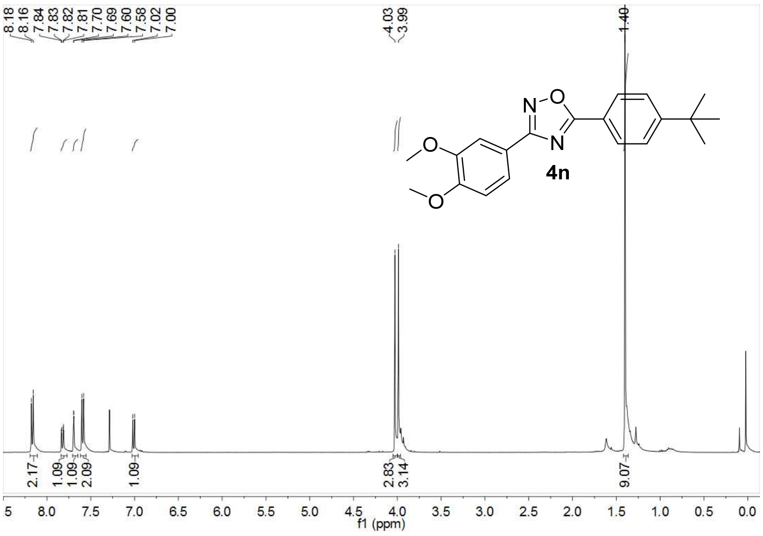

^{1}H NMR (400 MHz, $CDCl_3$)

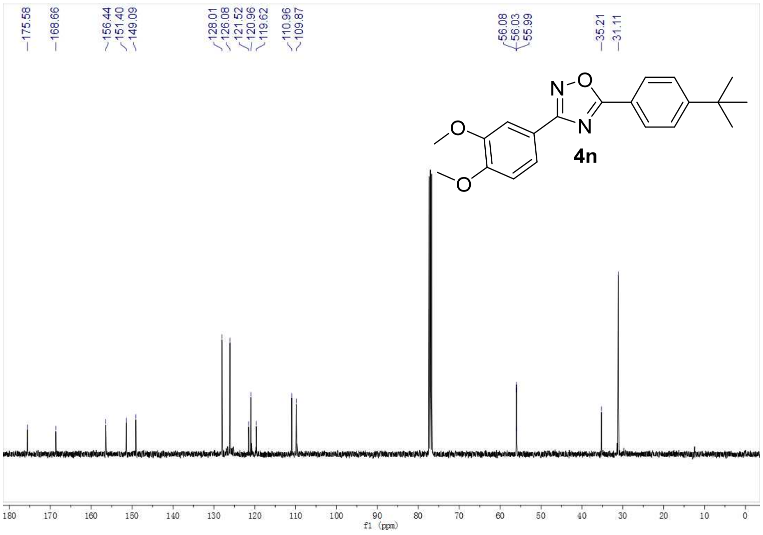

^{13}C NMR (101 MHz, $CDCl_3$)

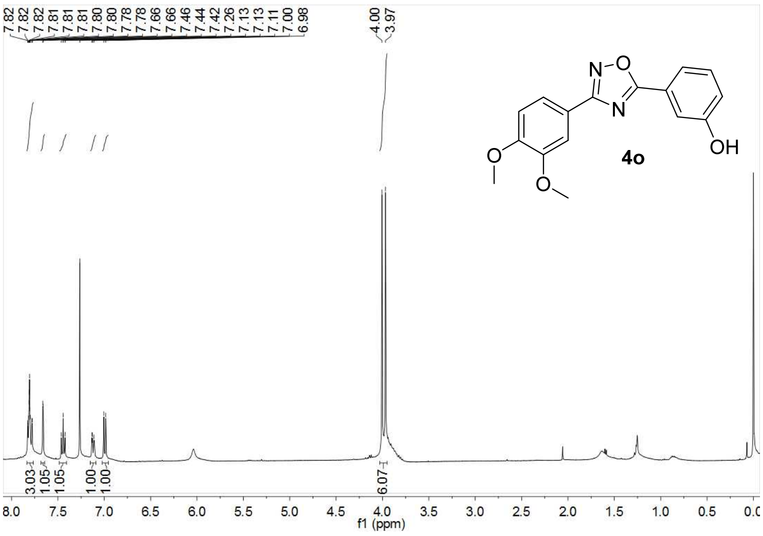

^{1}H NMR (400 MHz, $CDCl_3$)

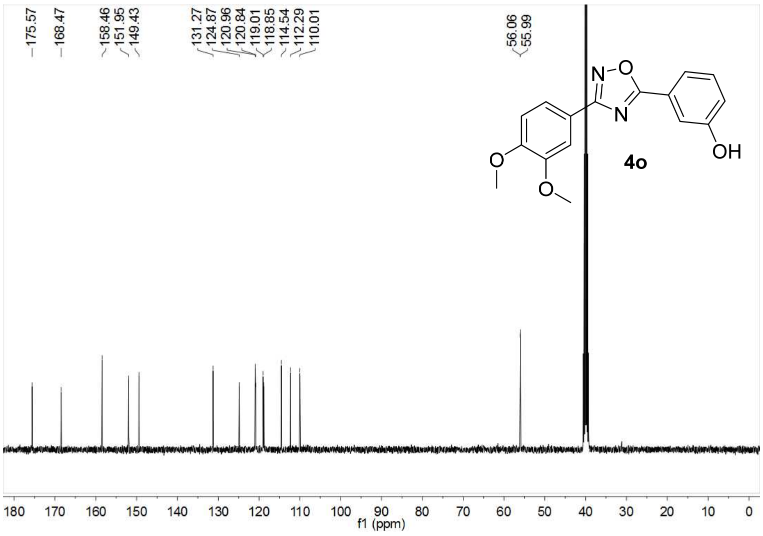

^{13}C NMR (101 MHz, DMSO-d6)

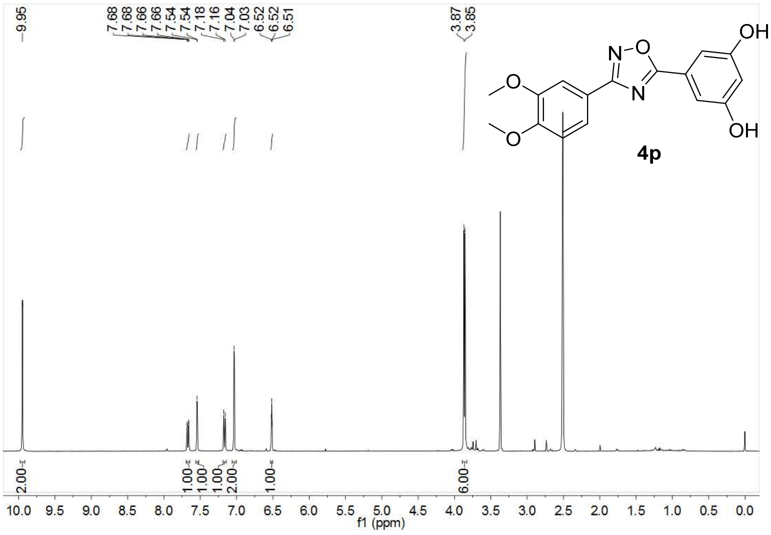

^1H NMR (400 MHz, DMSO-d6)

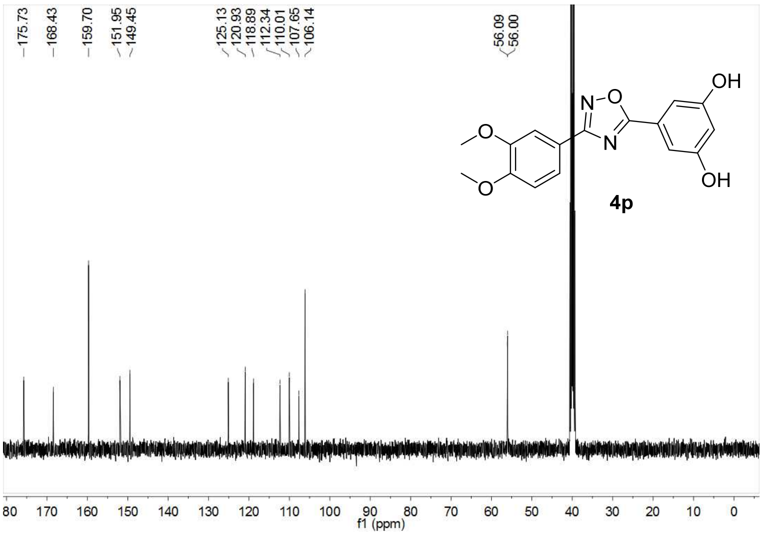

^{13}C NMR (101 MHz, DMSO-d6)

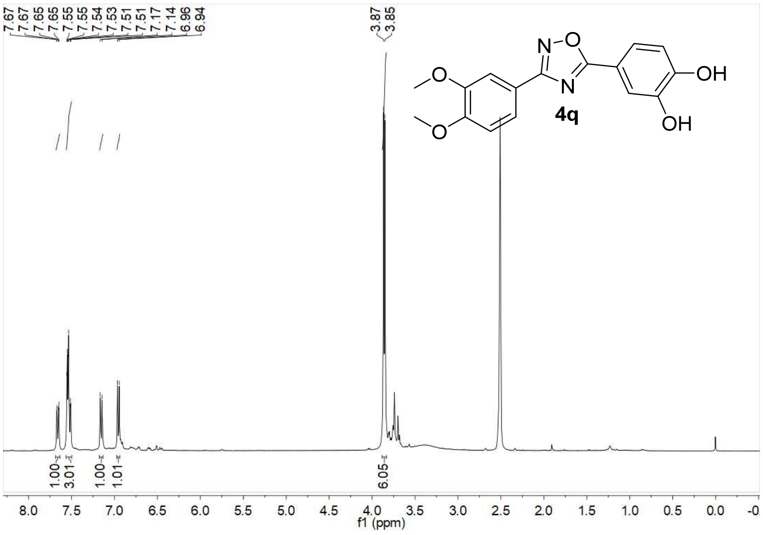

^{1}H NMR (400 MHz, DMSO-d6)

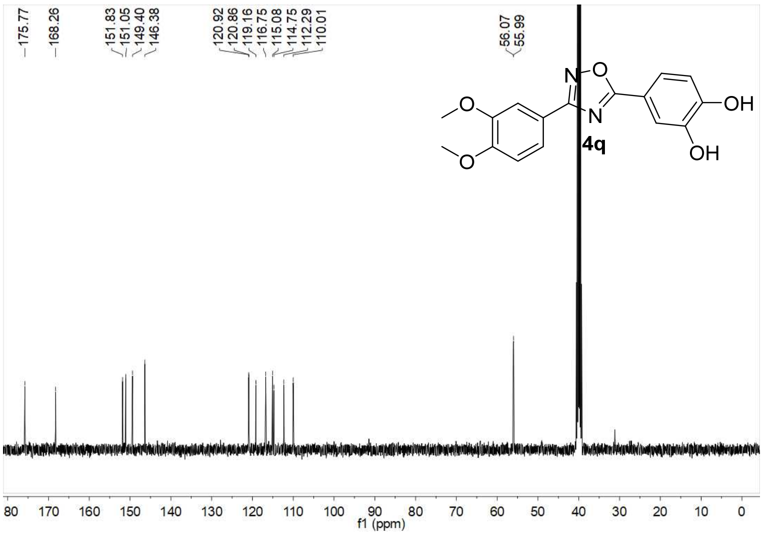

^{13}C NMR (101 MHz, DMSO-d6)

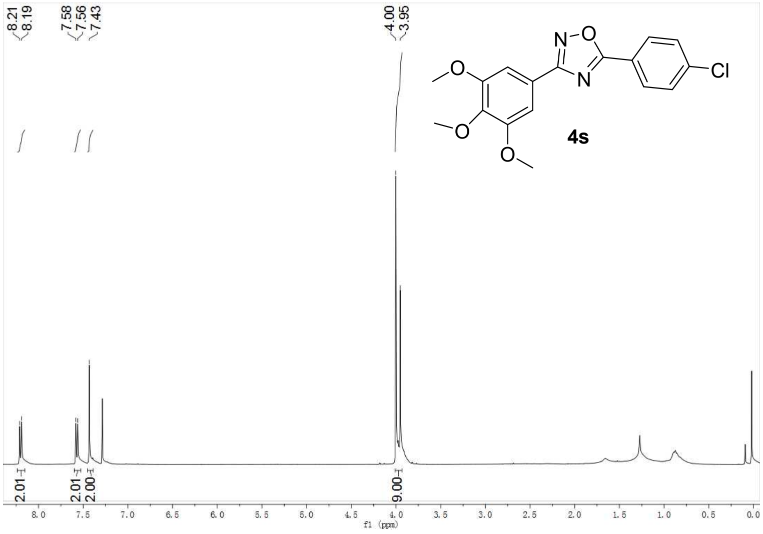

^{1}H NMR (400 MHz, $CDCl_3$)

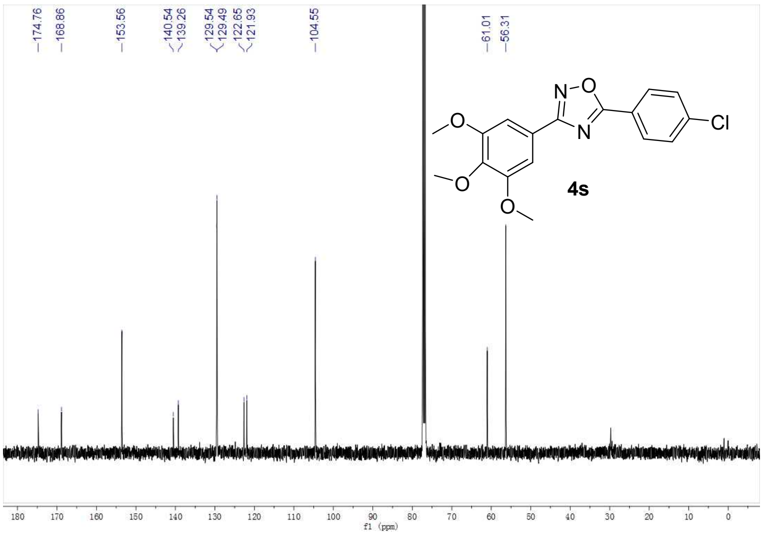

^{13}C NMR (101 MHz, $CDCl_3$)

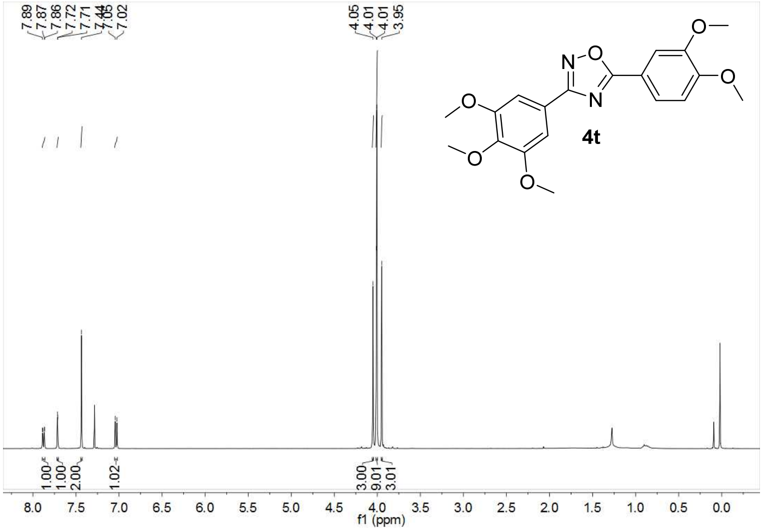

^{1}H NMR (400 MHz, $CDCl_3$)

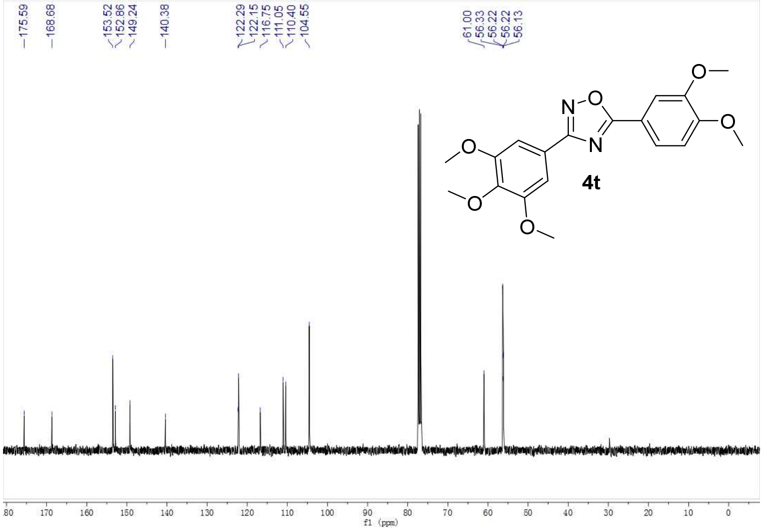

^{13}C NMR (101 MHz, $CDCl_3$)

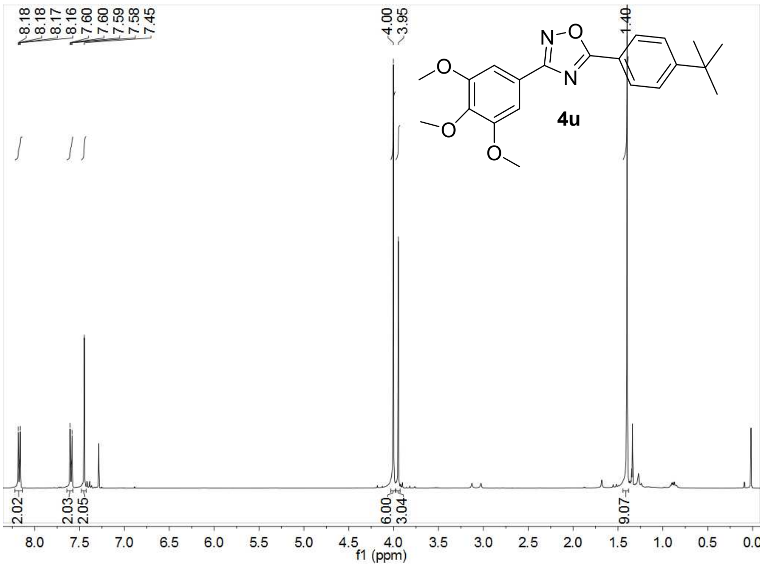

^{1}H NMR (400 MHz, $CDCl_3$)

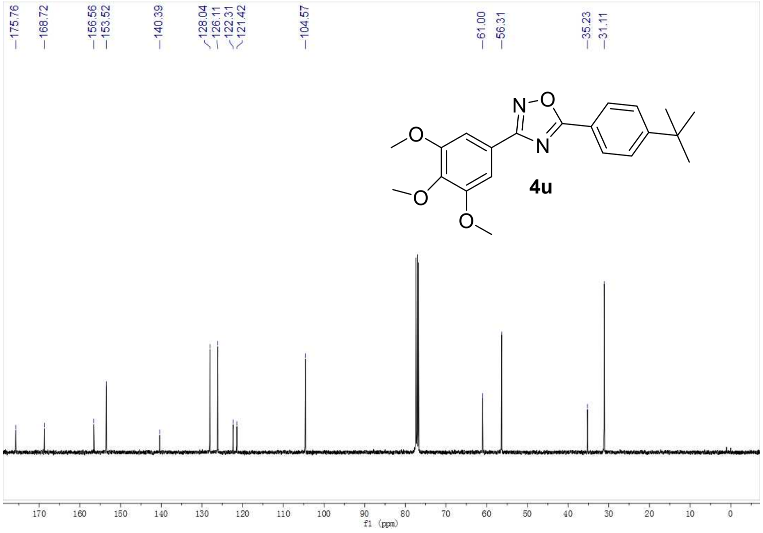

^{13}C NMR (101 MHz, $CDCl_3$)

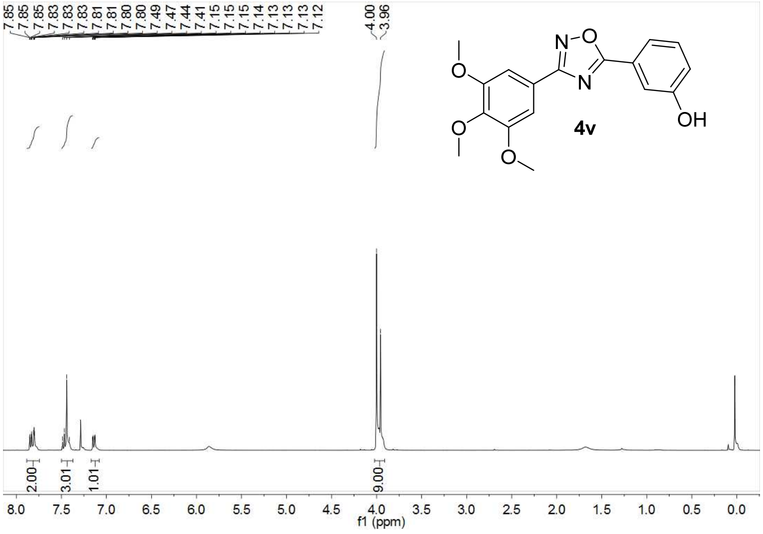

^{1}H NMR (400 MHz, $CDCl_3$)

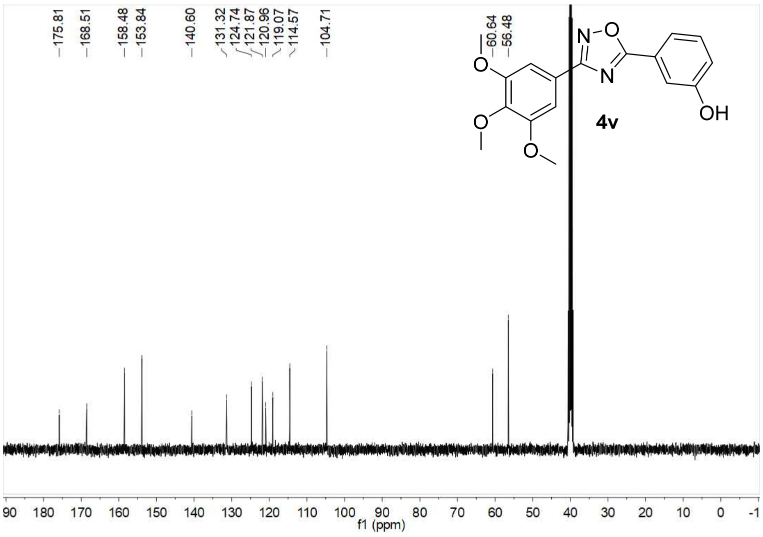

^{13}C NMR (101 MHz, DMSO-d6)

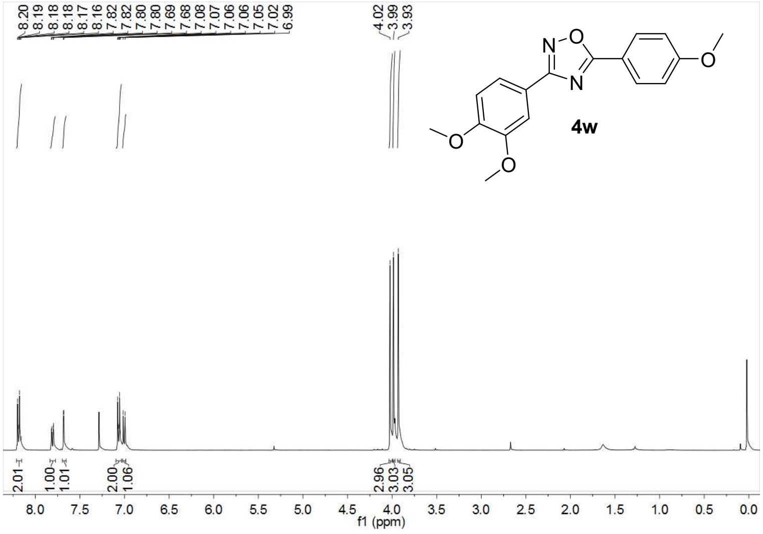

^{1}H NMR (400 MHz, $CDCl_3$)

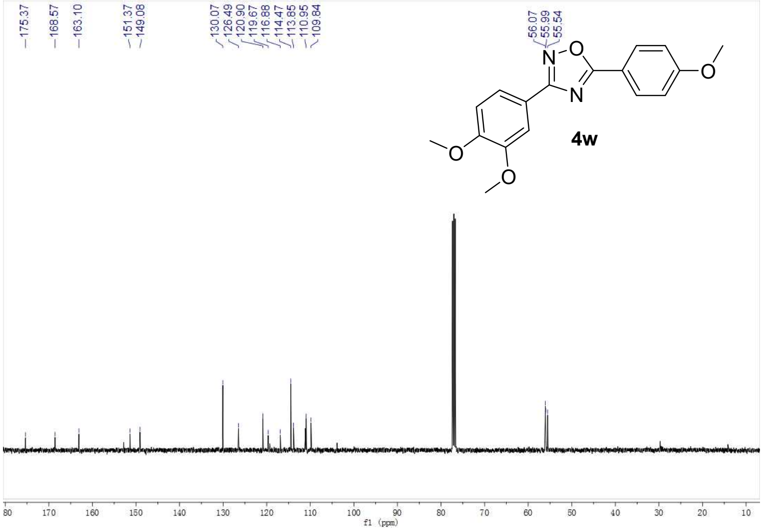

^{13}C NMR (101 MHz, $CDCl_3$)

Figure S2. ^{1}HNMR and ^{13}CNMR of 4a-4w

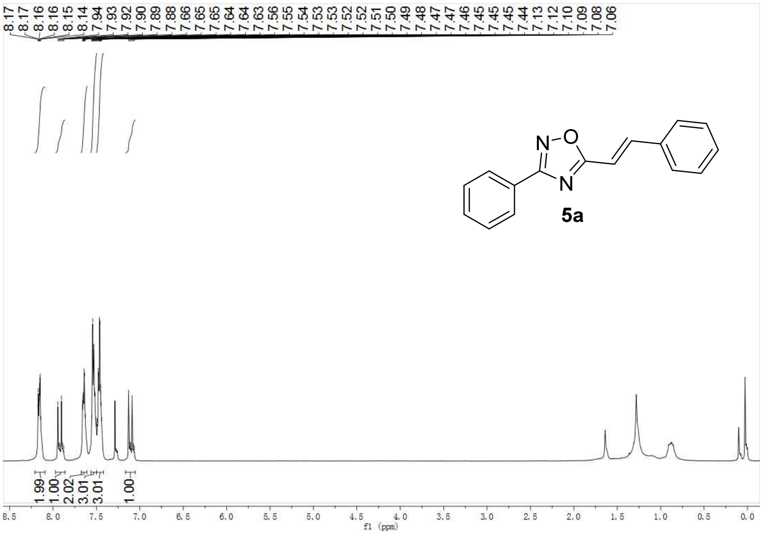

^{1}H NMR (400 MHz, $CDCl_3$)

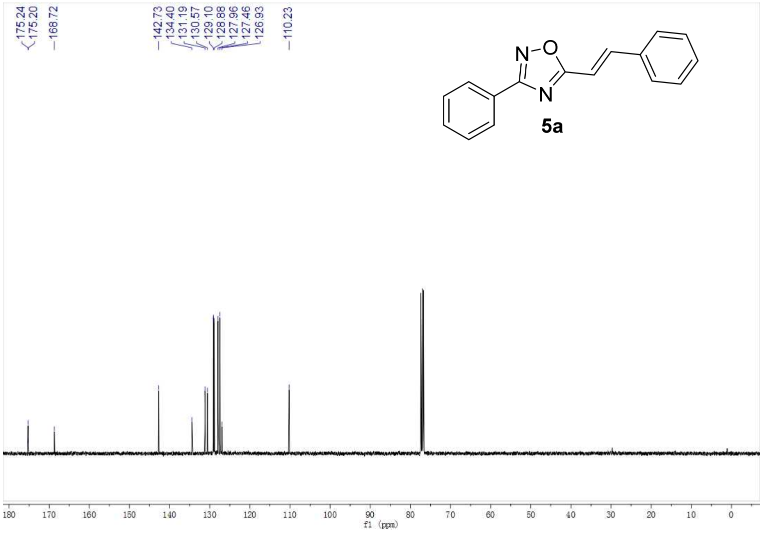

^{13}C NMR (101 MHz, $CDCl_3$)

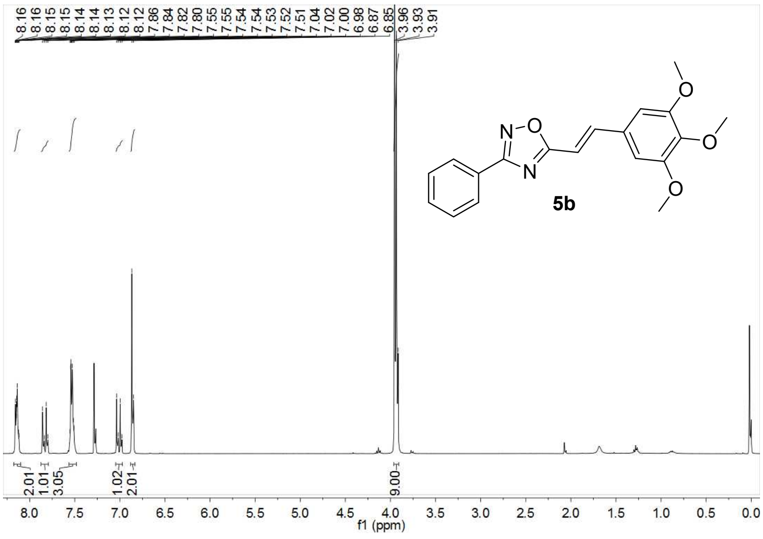

^{1}H NMR (400 MHz, $CDCl_3$)

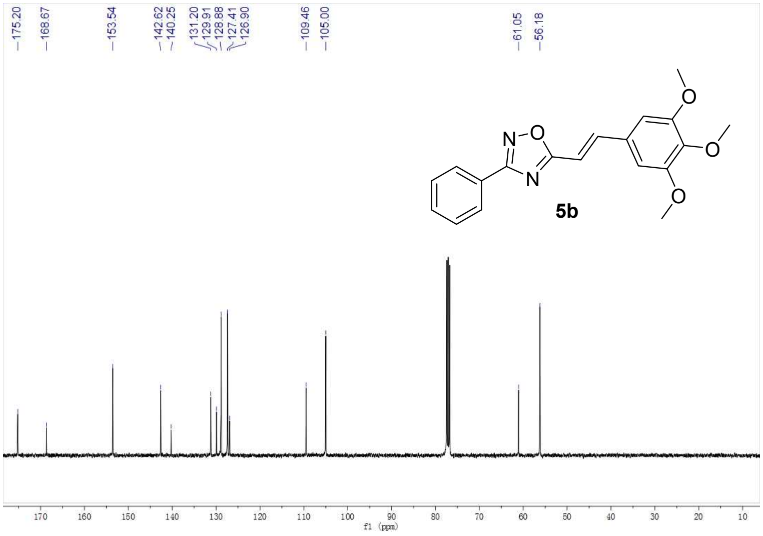

^{13}C NMR (101 MHz, $CDCl_3$)

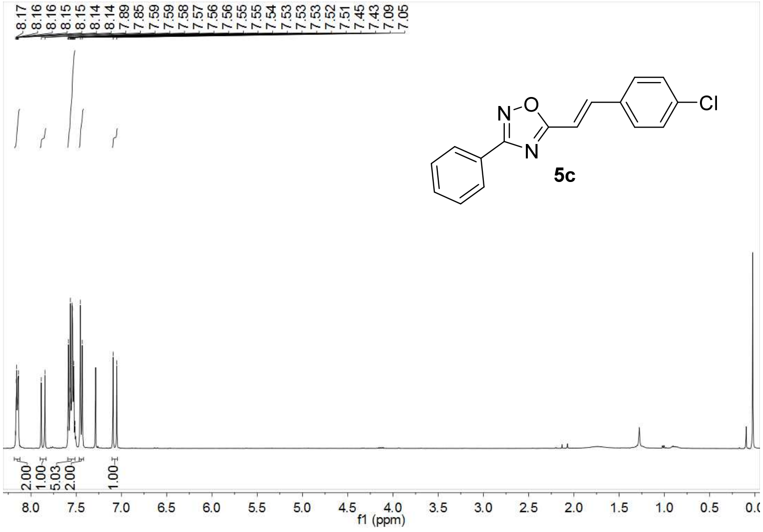

^{1}H NMR (400 MHz, $CDCl_3$)

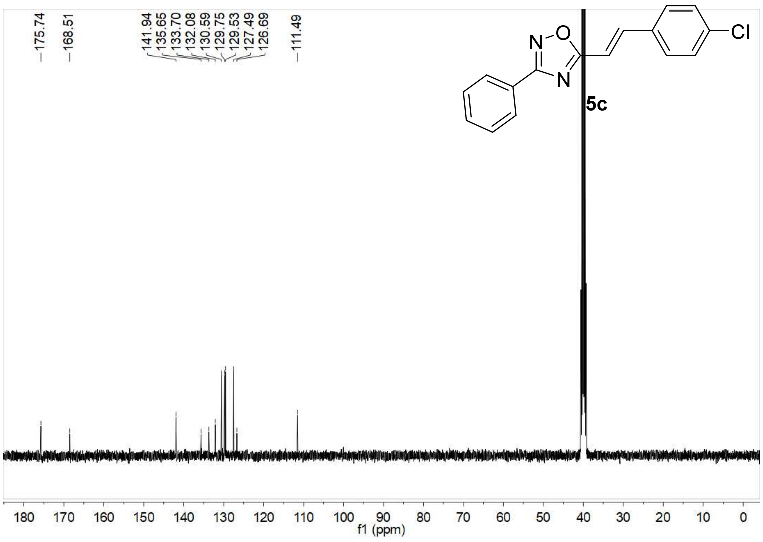

^{13}C NMR (101 MHz, DMSO-d6)

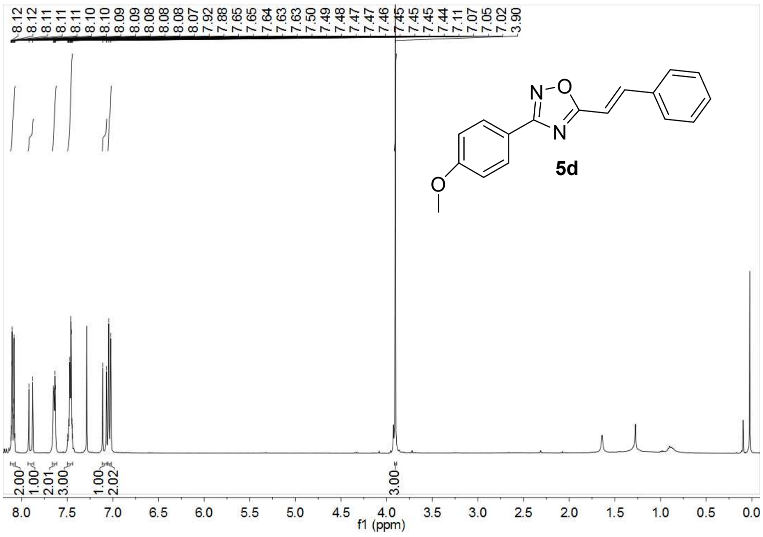

^{1}H NMR (400 MHz, $CDCl_3$)

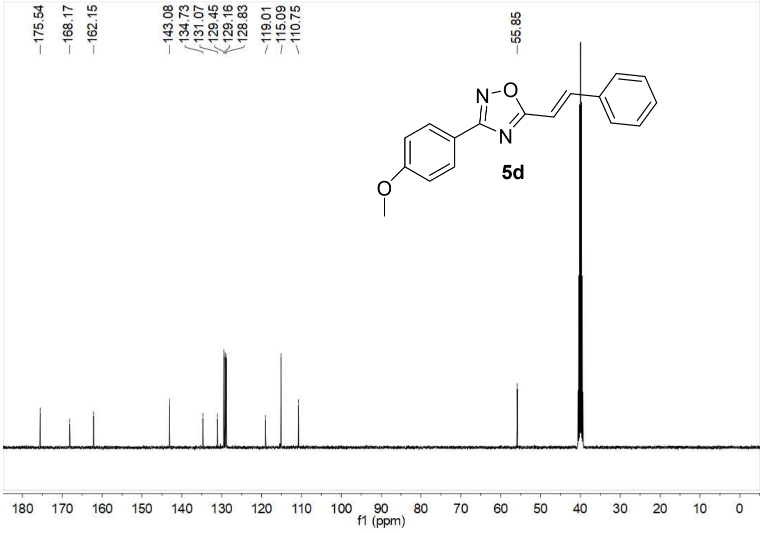

^{13}C NMR (101 MHz, DMSO-d6)

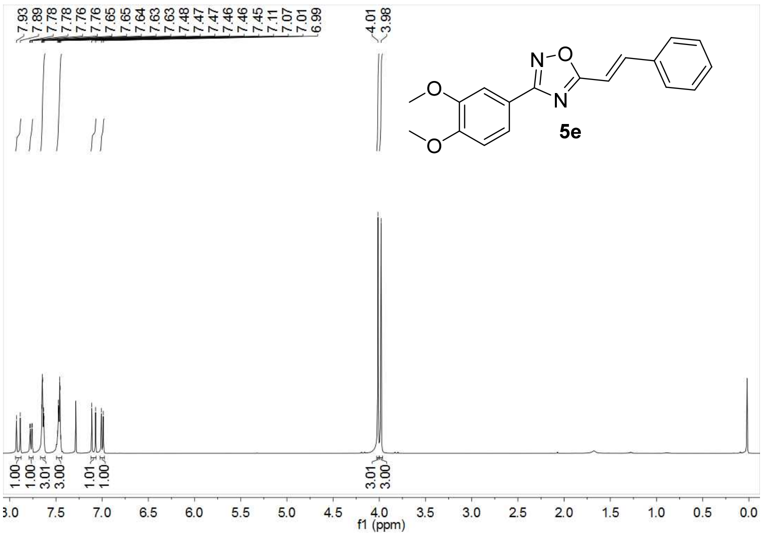

^{1}H NMR (400 MHz, $CDCl_3$)

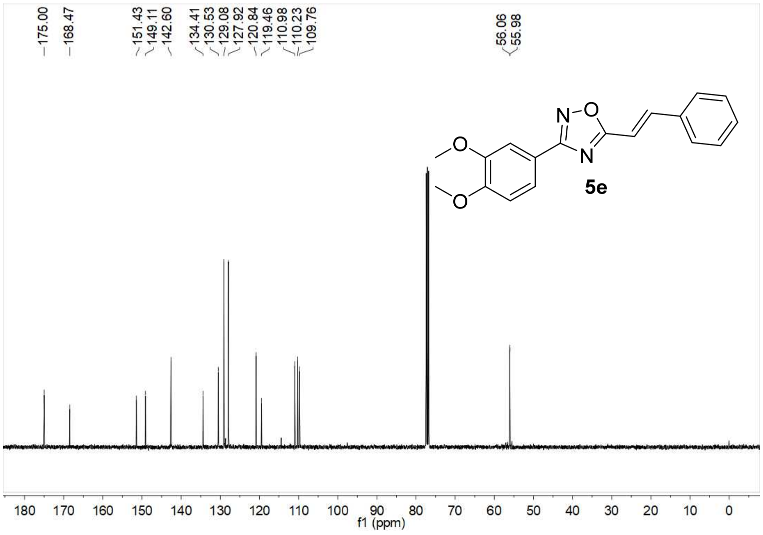

^{13}C NMR (101 MHz, $CDCl_3$)

Figure S3. ^{1}HNMR and ^{13}CNMR of 5a-5f

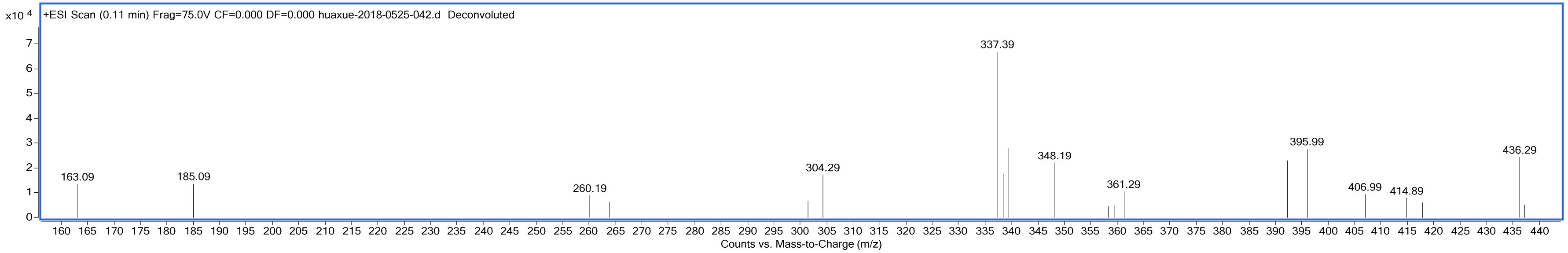

+ESI Scan (0.11 min) Frag=75.0V CF=0.000 DF=0.000 huaxue-2018-0525-042.d Deconvoluted
x10 4
163.09
185.09
260.19
304.29
337.39
348.19
361.29
395.99
406.99
414.89
436.29
Counts vs. Mass-to-Charge (m/z)

2d

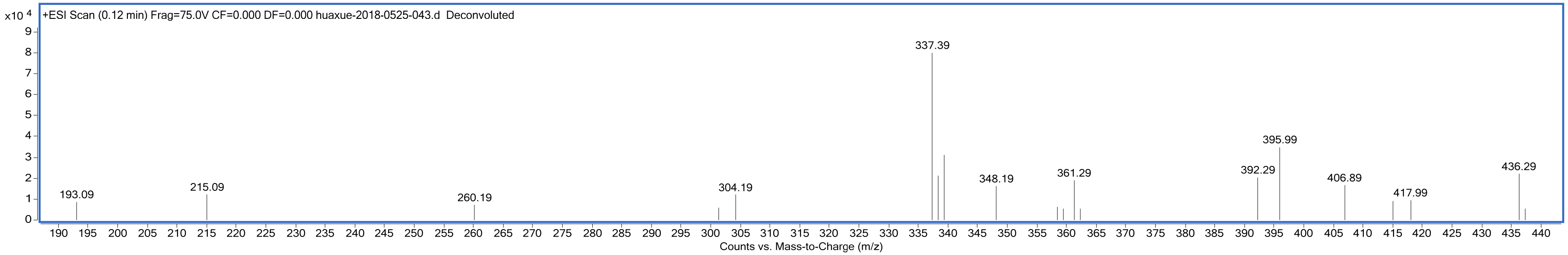

+ESI Scan (0.12 min) Frag=75.0V CF=0.000 DF=0.000 huaxue-2018-0525-043.d Deconvoluted
x10 4
193.09
215.09
260.19
304.19
337.39
348.19
361.29
392.29
395.99
406.89
417.99
436.29
Counts vs. Mass-to-Charge (m/z)

2e

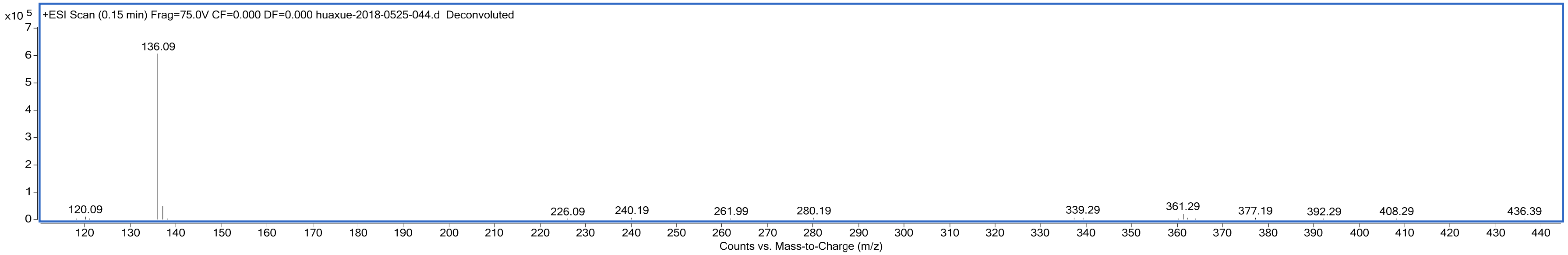

+ESI Scan (0.15 min) Frag=75.0V CF=0.000 DF=0.000 huaxue-2018-0525-044.d Deconvoluted
x10 5
120.09
136.09
226.09
240.19
261.99
280.19
339.29
361.29
377.19
392.29
408.29
436.39
Counts vs. Mass-to-Charge (m/z)

3a

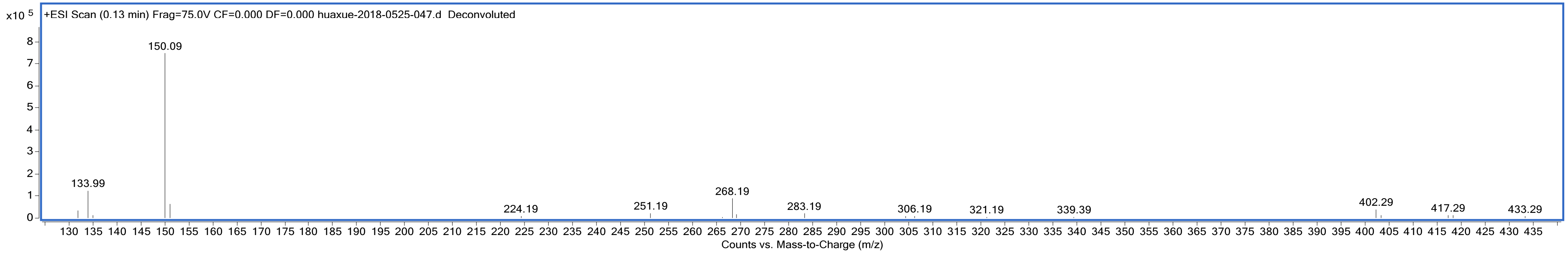
x10 5
+ESI Scan (0.13 min) Frag=75.0V CF=0.000 DF=0.000 huaxue-2018-0525-047.d Deconvoluted
150.09
133.99
224.19
251.19
268.19
283.19
306.19
321.19
339.39
402.29
417.29
433.29
Counts vs. Mass-to-Charge (m/z)

3b

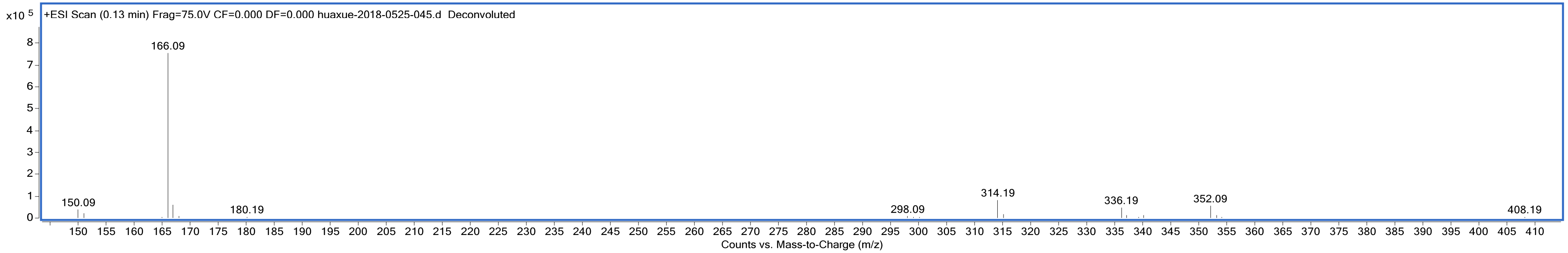
x10 5
+ESI Scan (0.13 min) Frag=75.0V CF=0.000 DF=0.000 huaxue-2018-0525-045.d Deconvoluted
166.09
150.09
180.19
298.09
314.19
336.19
352.09
408.19
Counts vs. Mass-to-Charge (m/z)

3c

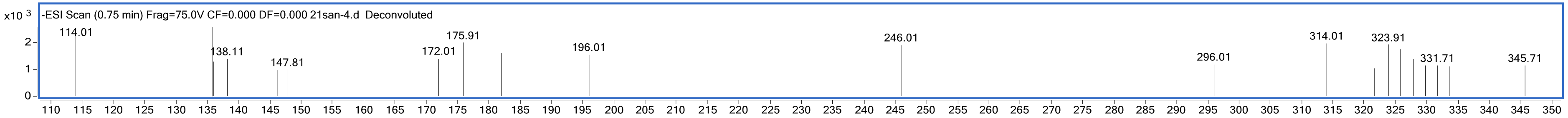
x10 3
-ESI Scan (0.75 min) Frag=75.0V CF=0.000 DF=0.000 21san-4.d Deconvoluted
114.01
138.11
147.81
172.01
175.91
196.01
246.01
296.01
314.01
323.91
331.71
345.71

3d

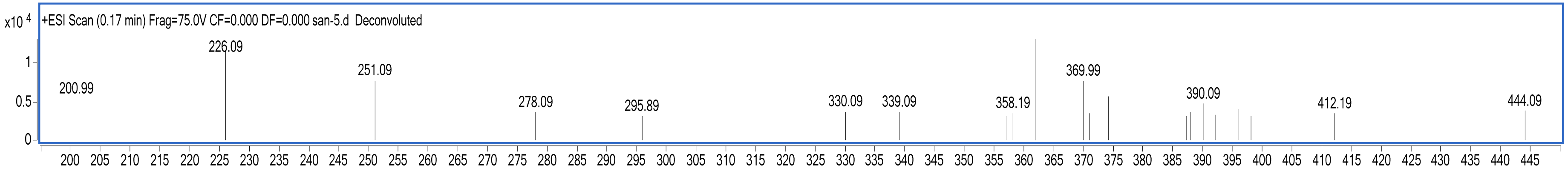
+ESI Scan (0.17 min) Frag=75.0V CF=0.000 DF=0.000 san-5.d Deconvoluted
x10 4
200.99
226.09
251.09
278.09
295.89
330.09
339.09
358.19
369.99
390.09
412.19
444.09

3e

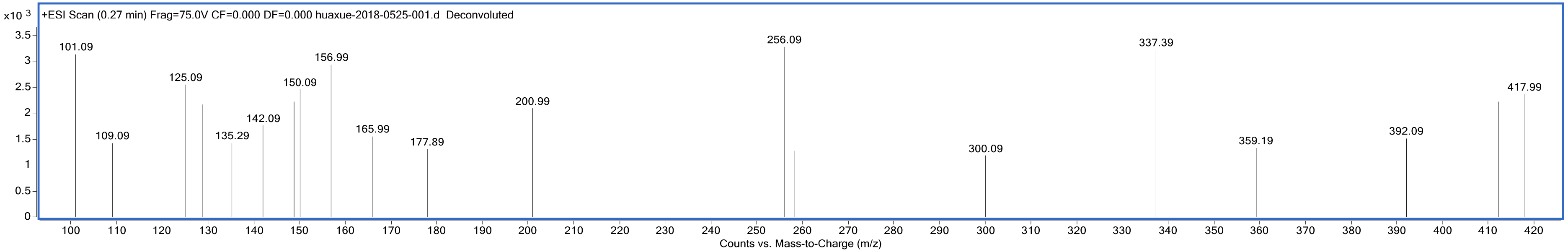
+ESI Scan (0.27 min) Frag=75.0V CF=0.000 DF=0.000 huaxue-2018-0525-001.d Deconvoluted
x10 3
101.09
109.09
125.09
135.29
142.09
150.09
156.99
165.99
177.89
200.99
256.09
300.09
337.39
359.19
392.09
417.99
Counts vs. Mass-to-Charge (m/z)

4a

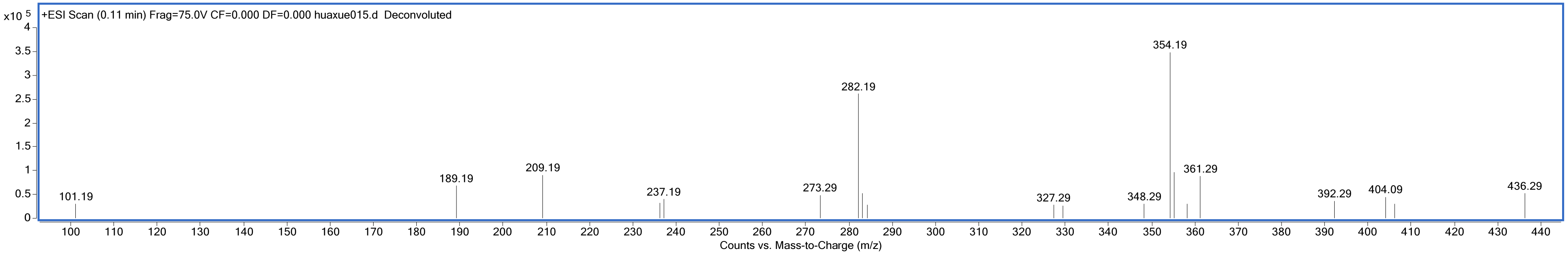
+ESI Scan (0.11 min) Frag=75.0V CF=0.000 DF=0.000 huaxue015.d Deconvoluted
x10 5
101.19
189.19
209.19
237.19
273.29
282.19
327.29
348.29
354.19
361.29
392.29
404.09
436.29
Counts vs. Mass-to-Charge (m/z)

4b

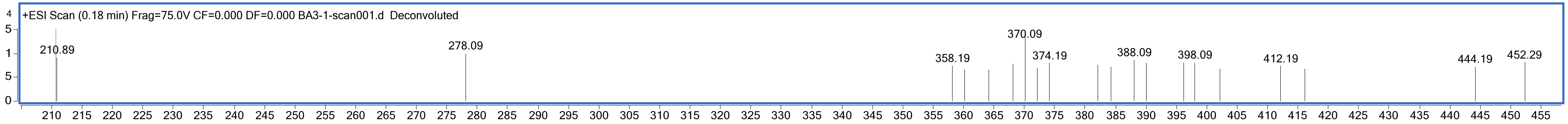
+ESI Scan (0.18 min) Frag=75.0V CF=0.000 DF=0.000 BA3-1-scan001.d Deconvoluted
210.89
278.09
358.19
370.09
374.19
388.09
398.09
412.19
444.19
452.29

4c

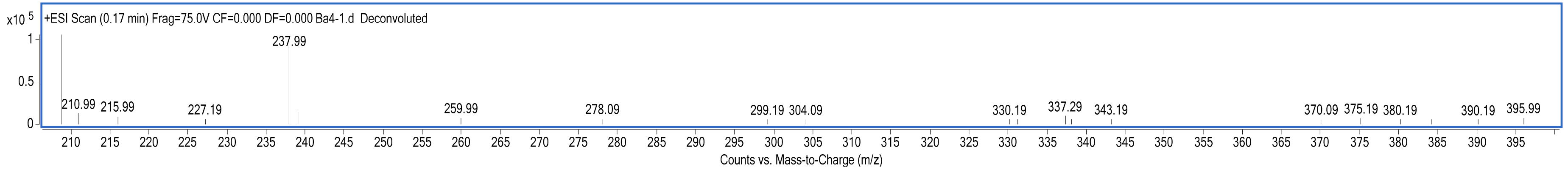
+ESI Scan (0.17 min) Frag=75.0V CF=0.000 DF=0.000 Ba4-1.d Deconvoluted
237.99
210.99
215.99
227.19
259.99
278.09
299.19
304.09
330.19
337.29
343.19
370.09
375.19
380.19
390.19
395.99
Counts vs. Mass-to-Charge (m/z)

4d

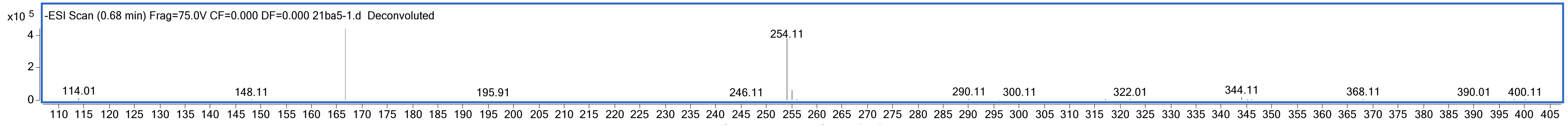
-ESI Scan (0.68 min) Frag=75.0V CF=0.000 DF=0.000 21ba5-1.d Deconvoluted
254.11
114.01
148.11
195.91
246.11
290.11
300.11
322.01
344.11
368.11
390.01
400.11

4e

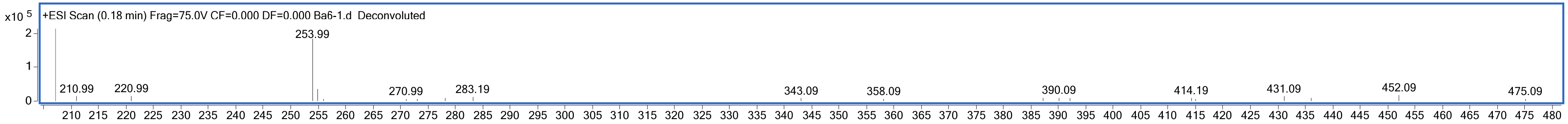
+ESI Scan (0.18 min) Frag=75.0V CF=0.000 DF=0.000 Ba6-1.d Deconvoluted
253.99
210.99
220.99
270.99
283.19
343.09
358.09
390.09
414.19
431.09
452.09
475.09

4f

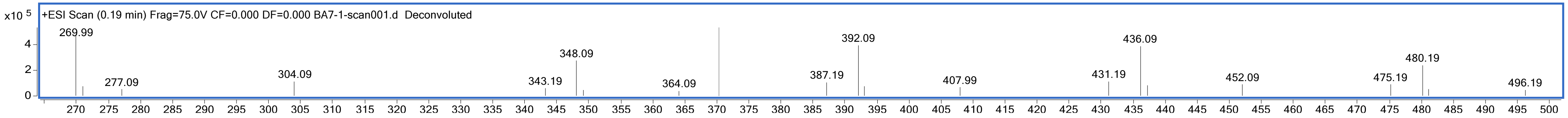
+ESI Scan (0.19 min) Frag=75.0V CF=0.000 DF=0.000 BA7-1-scan001.d Deconvoluted
269.99
277.09
304.09
343.19
348.09
364.09
387.19
392.09
407.99
431.19
436.09
452.09
475.19
480.19
496.19

4g

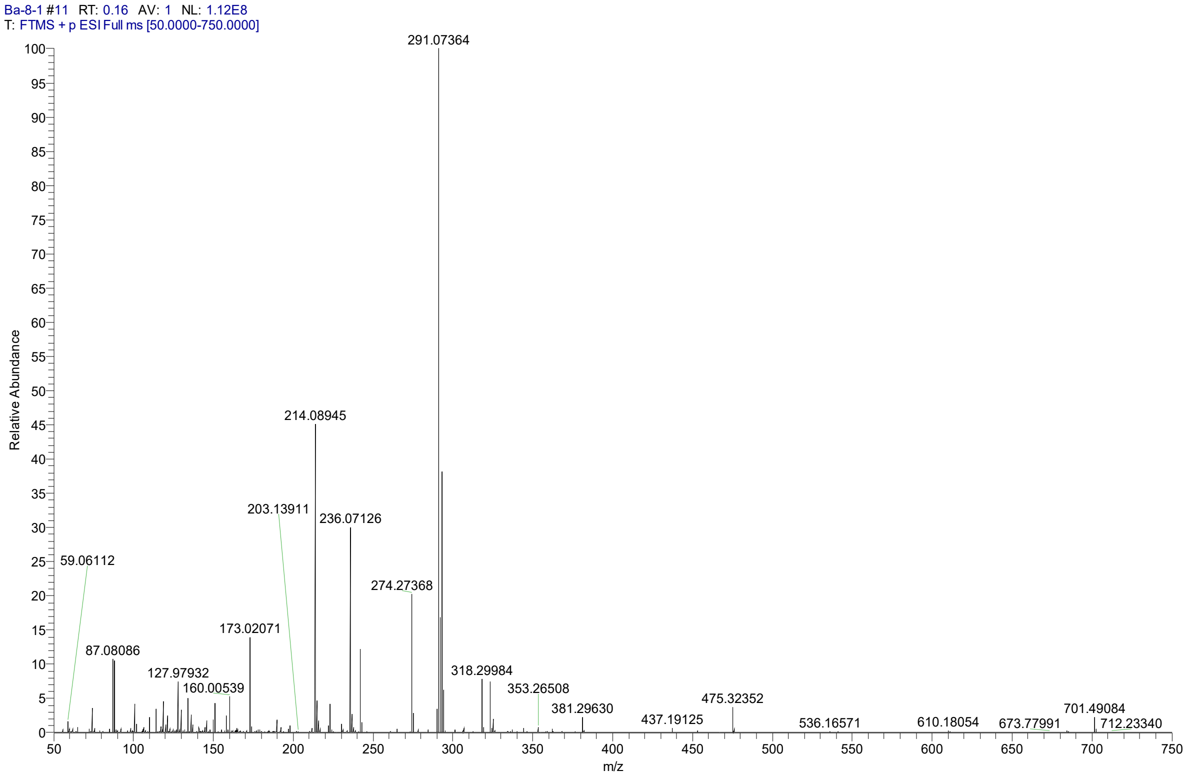
Ba-8-1 #11 RT: 0.16 AV: 1 NL: 1.12E8
T: FTMS + p ESI Full ms [50.0000-750.0000]
291.07364
214.08945
203.13911
236.07126
59.06112
274.27368
173.02071
87.08086
127.97932
160.00539
318.29984
353.26508
381.29630
437.19125
475.32352
536.16571
610.18054
673.77991
701.49084
712.23340
Relative Abundance
m/z

4h

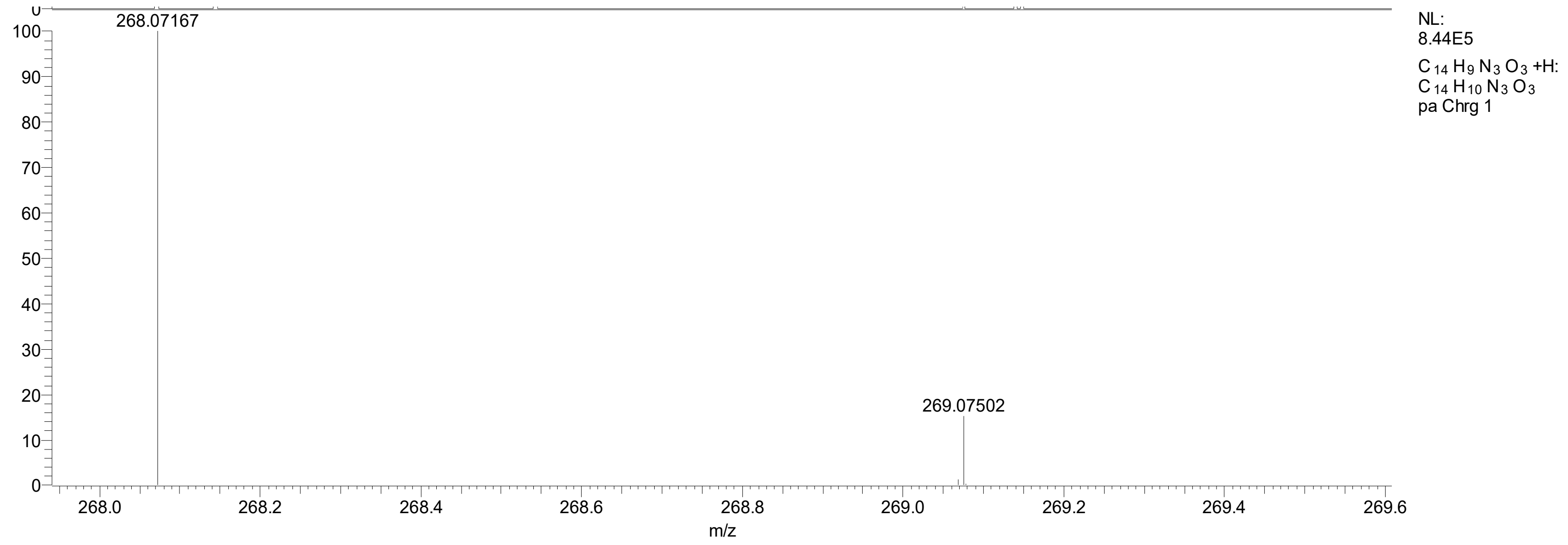

NL:
8.44E5
C 14 H9 N3 O3 +H:
C 14 H10 N3 O3
pa Chrg 1
268.07167
269.07502
m/z

4i

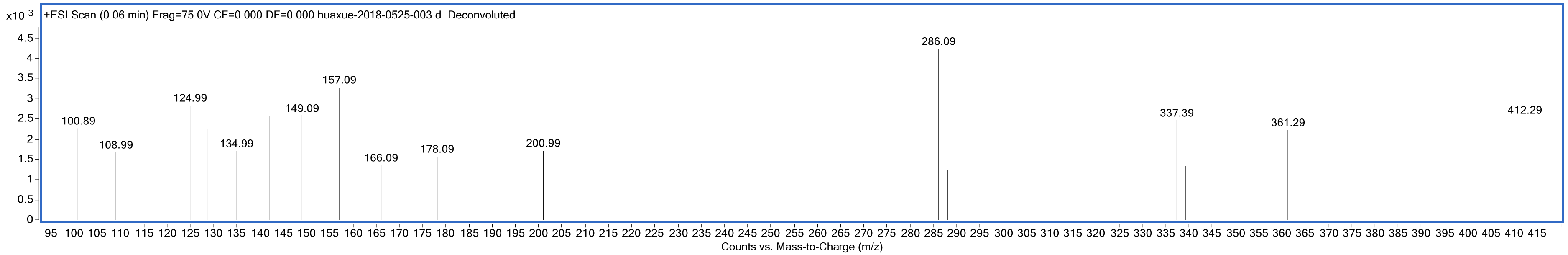

x10 3
+ESI Scan (0.06 min) Frag=75.0V CF=0.000 DF=0.000 huaxue-2018-0525-003.d Deconvoluted
286.09
157.09
124.99
149.09
100.89
108.99
134.99
166.09
178.09
200.99
337.39
361.29
412.29
Counts vs. Mass-to-Charge (m/z)

4j

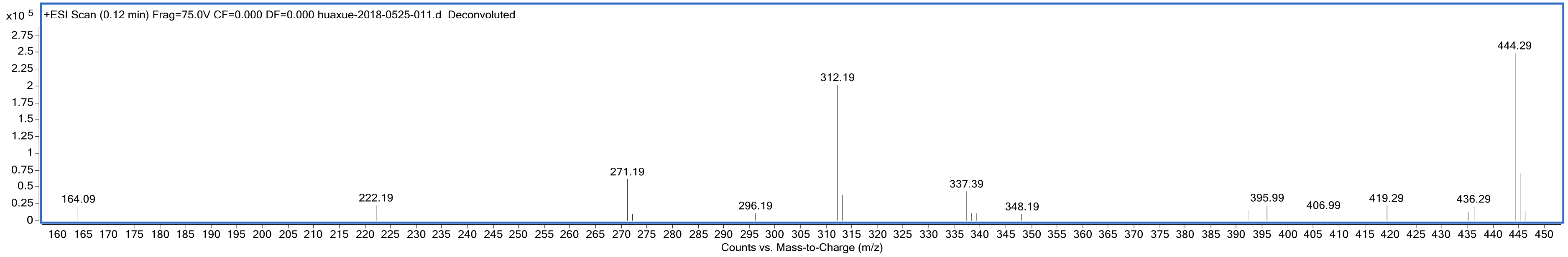
+ESI Scan (0.12 min) Frag=75.0V CF=0.000 DF=0.000 huaxue-2018-0525-011.d Deconvoluted
x10 5
164.09
222.19
271.19
296.19
312.19
337.39
348.19
395.99
406.99
419.29
436.29
444.29
Counts vs. Mass-to-Charge (m/z)

4k

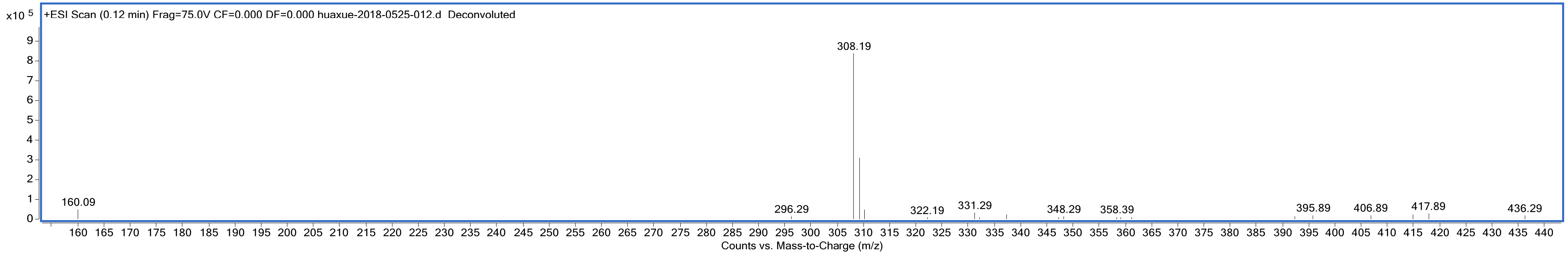
+ESI Scan (0.12 min) Frag=75.0V CF=0.000 DF=0.000 huaxue-2018-0525-012.d Deconvoluted
x10 5
160.09
296.29
308.19
322.19
331.29
348.29
358.39
395.89
406.89
417.89
436.29
Counts vs. Mass-to-Charge (m/z)

4l

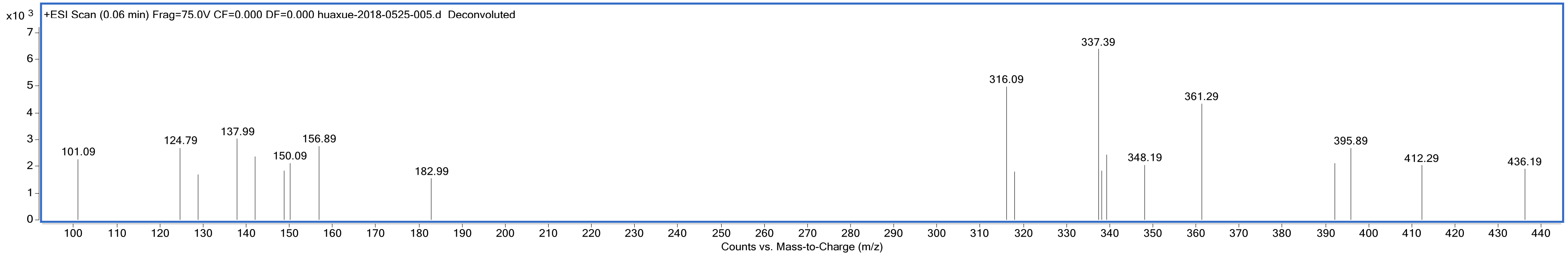
+ESI Scan (0.06 min) Frag=75.0V CF=0.000 DF=0.000 huaxue-2018-0525-005.d Deconvoluted
x10 3
101.09
124.79
137.99
150.09
156.89
182.99
316.09
337.39
348.19
361.29
395.89
412.29
436.19
Counts vs. Mass-to-Charge (m/z)

4m

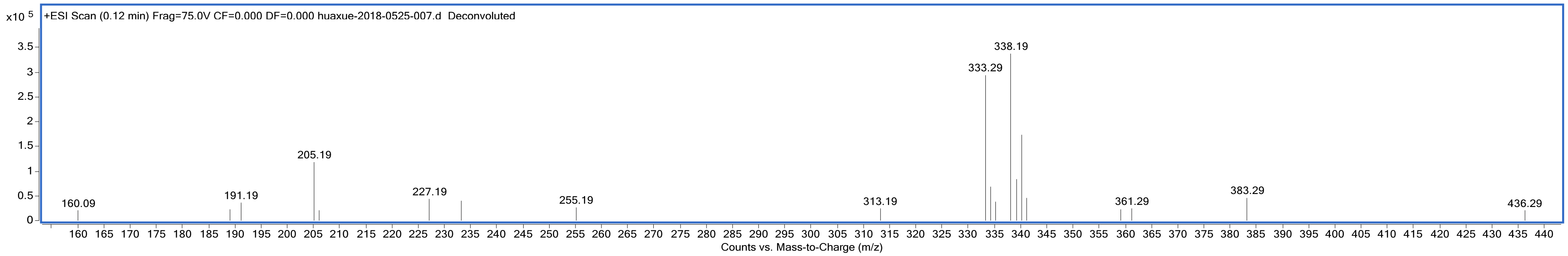
x10 5
+ESI Scan (0.12 min) Frag=75.0V CF=0.000 DF=0.000 huaxue-2018-0525-007.d Deconvoluted
160.09
191.19
205.19
227.19
255.19
313.19
333.29
338.19
361.29
383.29
436.29
Counts vs. Mass-to-Charge (m/z)

4n

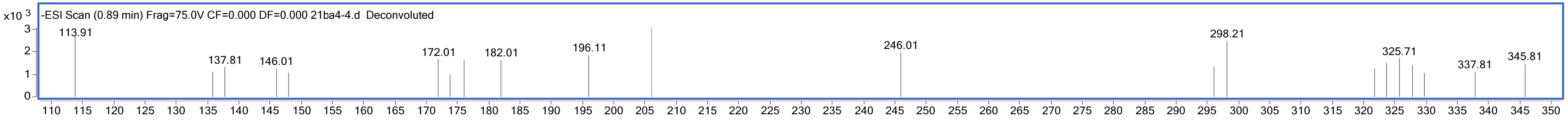
x10 3
-ESI Scan (0.89 min) Frag=75.0V CF=0.000 DF=0.000 21ba4-4.d Deconvoluted
113.91
137.81
146.01
172.01
182.01
196.11
246.01
298.21
325.71
337.81
345.81

4o

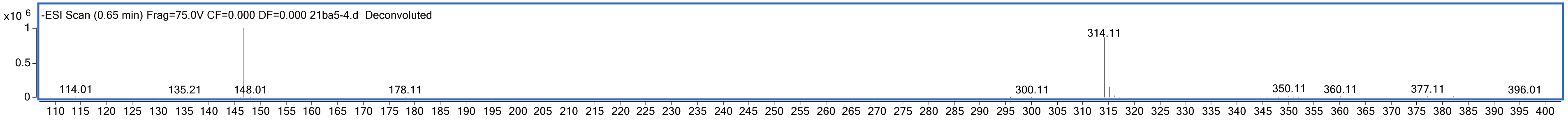
x10 6
-ESI Scan (0.65 min) Frag=75.0V CF=0.000 DF=0.000 21ba5-4.d Deconvoluted
114.01
135.21
148.01
178.11
300.11
314.11
350.11
360.11
377.11
396.01

4p

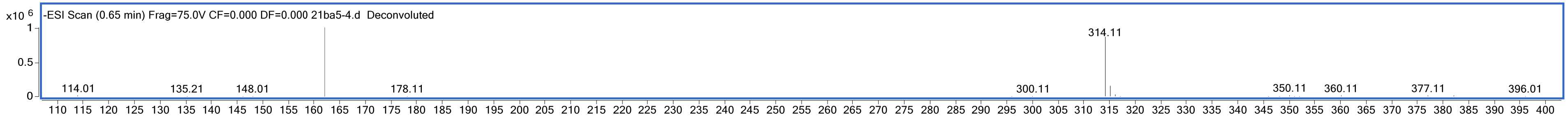
x10 6
-ESI Scan (0.65 min) Frag=75.0V CF=0.000 DF=0.000 21ba5-4.d Deconvoluted
114.01
135.21
148.01
178.11
300.11
314.11
350.11
360.11
377.11
396.01

4q

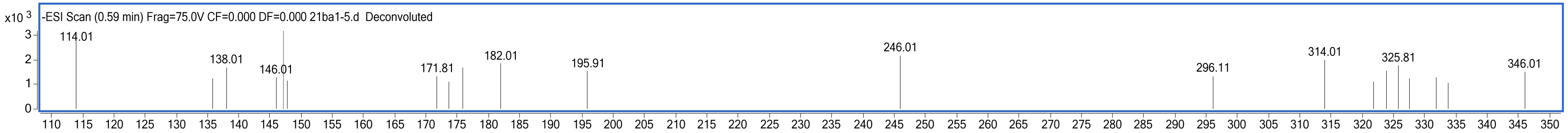
x10 3
-ESI Scan (0.59 min) Frag=75.0V CF=0.000 DF=0.000 21ba1-5.d Deconvoluted
114.01
138.01
146.01
171.81
182.01
195.91
246.01
296.11
314.01
325.81
346.01

4s

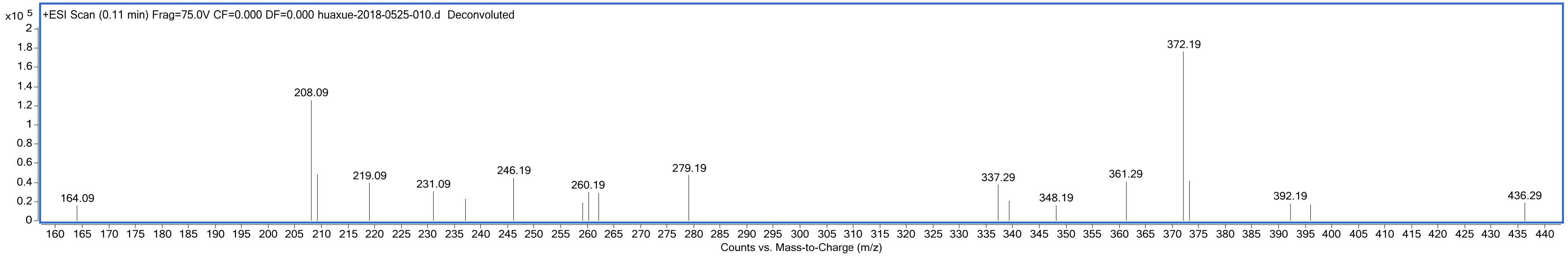
x10 5
+ESI Scan (0.11 min) Frag=75.0V CF=0.000 DF=0.000 huaxue-2018-0525-010.d Deconvoluted
164.09
208.09
219.09
231.09
246.19
260.19
279.19
337.29
348.19
361.29
372.19
392.19
436.29
Counts vs. Mass-to-Charge (m/z)

4t

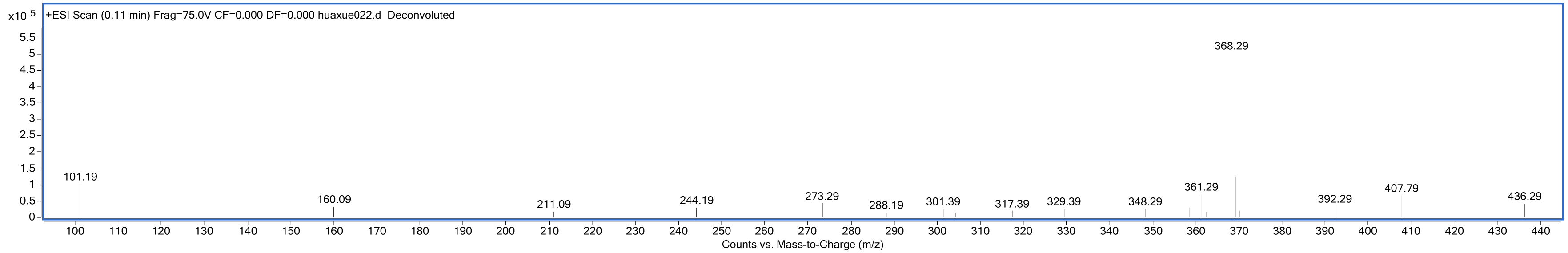
x10 5
+ESI Scan (0.11 min) Frag=75.0V CF=0.000 DF=0.000 huaxue022.d Deconvoluted
101.19
160.09
211.09
244.19
273.29
288.19
301.39
317.39
329.39
348.29
361.29
368.29
392.29
407.79
436.29
Counts vs. Mass-to-Charge (m/z)

4u

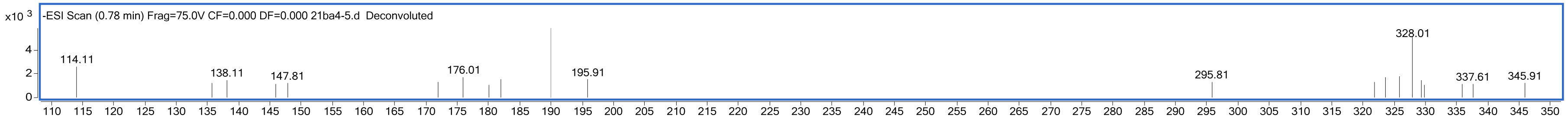
x10 3
-ESI Scan (0.78 min) Frag=75.0V CF=0.000 DF=0.000 21ba4-5.d Deconvoluted
114.11
138.11
147.81
176.01
195.91
295.81
328.01
337.61
345.91

4v

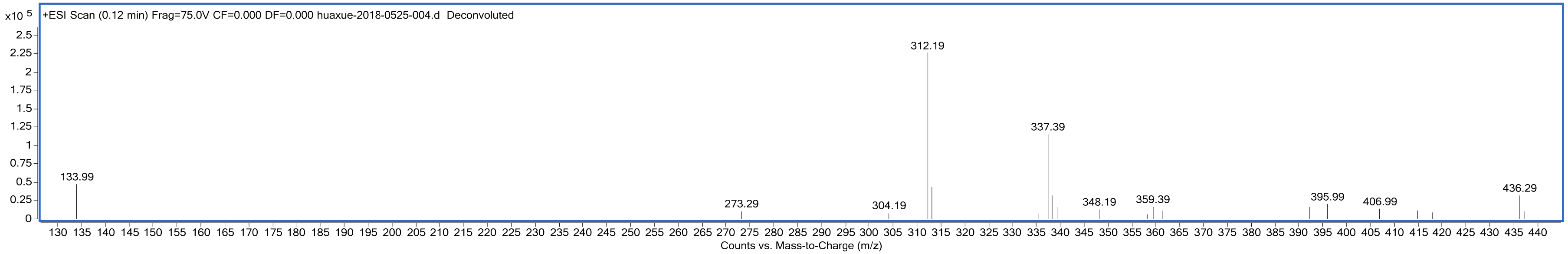
+ESI Scan (0.12 min) Frag=75.0V CF=0.000 DF=0.000 huaxue-2018-0525-004.d Deconvoluted
x10 5
Counts vs. Mass-to-Charge (m/z)
312.19
337.39
133.99
273.29
304.19
348.19
359.39
395.99
406.99
436.29

4w

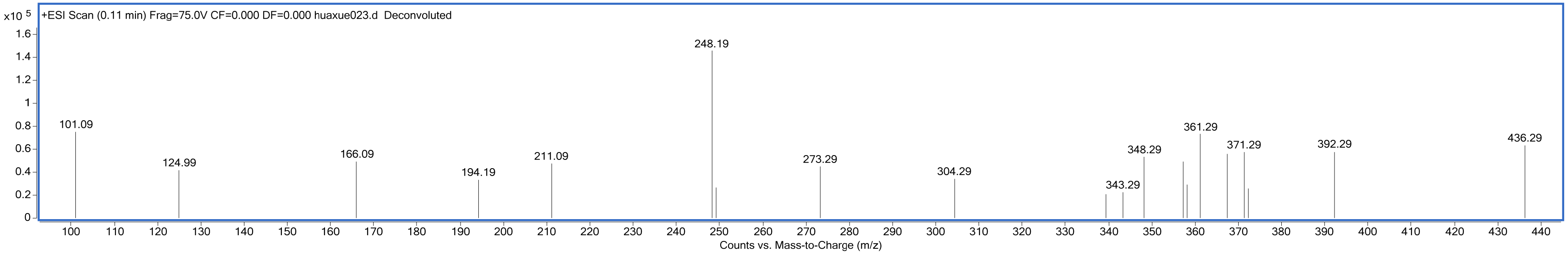
+ESI Scan (0.11 min) Frag=75.0V CF=0.000 DF=0.000 huaxue023.d Deconvoluted
x10 5
Counts vs. Mass-to-Charge (m/z)
248.19
101.09
124.99
166.09
194.19
211.09
273.29
304.29
343.29
348.29
361.29
371.29
392.29
436.29

5a

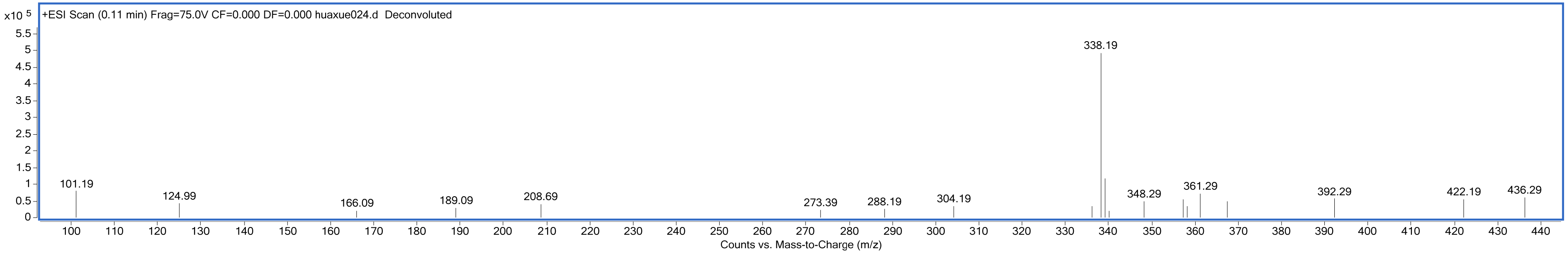
+ESI Scan (0.11 min) Frag=75.0V CF=0.000 DF=0.000 huaxue024.d Deconvoluted
x10 5
Counts vs. Mass-to-Charge (m/z)
338.19
101.19
124.99
166.09
189.09
208.69
273.39
288.19
304.19
348.29
361.29
392.29
422.19
436.29

5b

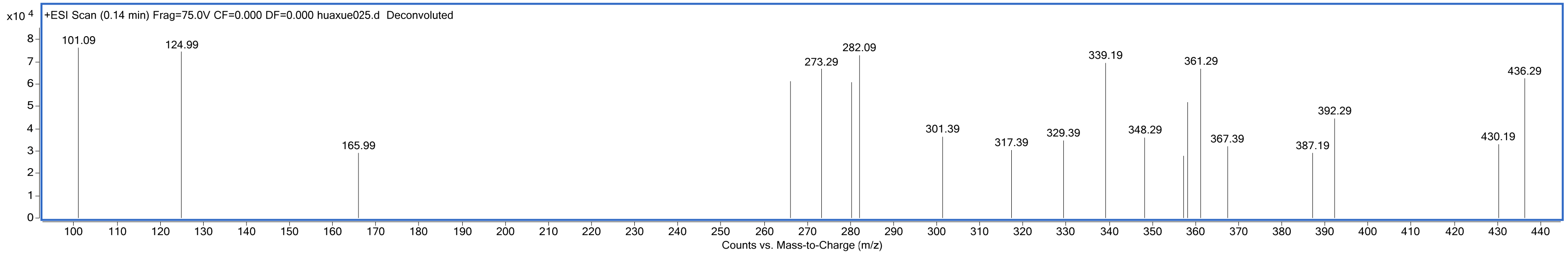

5c

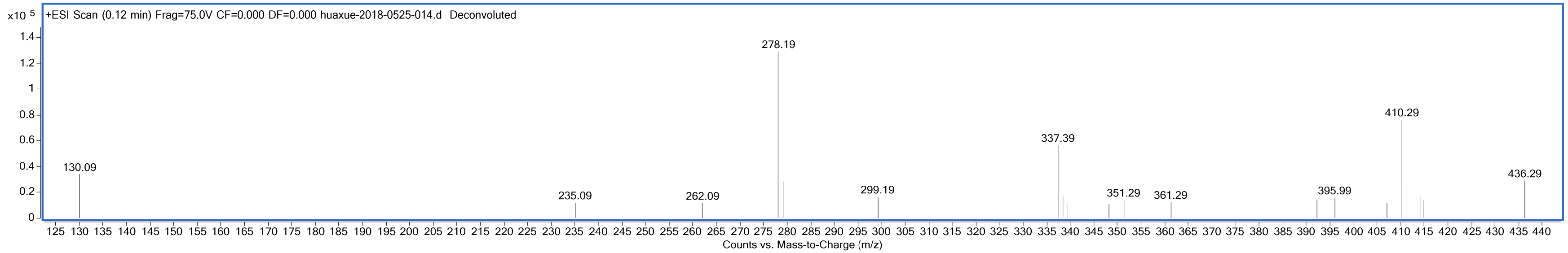

5d

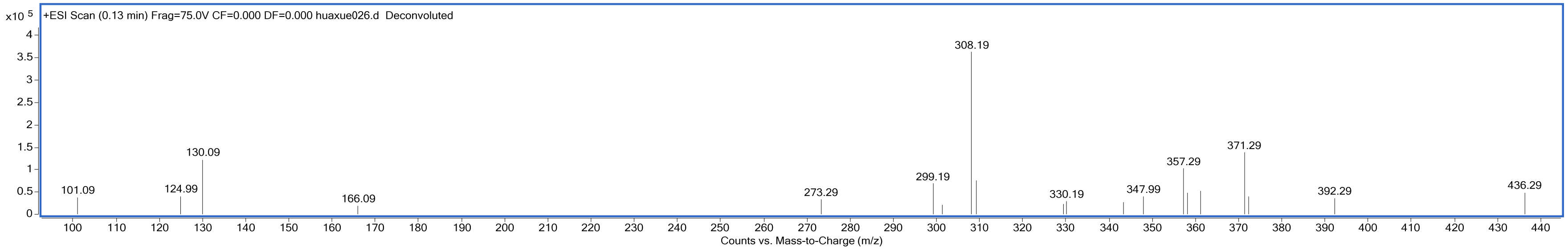

5e

Figure S4. Mass spectra

Table S2. The primary data for table 1(*R. solani*)

R. solani	The value of the diameter of the plaque(mm)												The average inhibition rate (%)	SD
	Group 1		Group 2		Group 3		Blank 1		Blank 2		Blank 3			
Compound	x	y	x	y	x	y	x	y	x	y	x	y		
4a	20.52	21.90	22.50	21.66	20.98	20.10	22.74	23.74	22.50	23.30	23.26	23.54	9.43	3.83
4b	17.86	17.40	19.20	20.20	20.60	18.40	23.06	22.80	21.80	23.08	22.32	22.58	18.68	5.82
4c	20.60	19.24	20.80	19.82	21.40	21.14	23.06	22.80	21.80	23.08	22.32	22.58	10.74	3.54
4d	20.80	18.34	19.24	18.20	18.46	18.72	23.06	22.80	21.80	23.08	22.32	22.58	18.60	2.71
4e	19.06	19.38	19.22	20.14	18.30	20.00	23.06	22.80	21.80	23.08	22.32	22.58	16.61	1.47
4f	6.00	5.66	6.56	6.40	6.14	5.48	18.86	18.30	18.38	19.66	19.84	18.32	80.87	2.40
4g	12.54	13.82	12.30	12.12	12.16	12.36	18.86	18.30	18.38	19.66	19.84	18.32	39.91	3.44
4h	22.80	23.86	25.10	23.36	25.26	24.22	22.74	23.74	22.50	23.30	23.26	23.54	-4.56	3.54
4i	19.54	18.76	19.12	18.54	20.16	19.42	18.86	18.30	18.38	19.66	19.84	18.32	-2.29	3.08
4j	-	-	21.90	21.38	21.34	22.60	22.74	23.74	22.50	23.30	23.26	23.54	6.81	1.16
4k	15.30	17.06	19.62	18.42	20.26	18.40	23.06	22.80	21.80	23.08	22.32	22.58	22.59	8.85
4l	22.24	20.80	21.14	21.52	22.60	23.12	22.74	23.74	22.50	23.30	23.26	23.54	6.33	4.13
4m	22.22	20.70	20.00	19.62	19.52	19.78	23.06	22.80	21.80	23.08	22.32	22.58	11.73	5.11
4n	21.42	20.64	22.12	20.32	22.08	21.24	23.06	22.80	21.80	23.08	22.32	22.58	6.65	1.65
4o	18.08	18.14	17.90	19.36	18.76	17.26	22.74	23.74	22.50	23.30	23.26	23.54	24.43	1.65
4p	22.00	21.44	22.86	20.54	20.94	20.86	23.06	22.80	21.80	23.08	22.32	22.58	5.95	2.39
4q	12.14	11.60	12.86	12.88	15.30	14.68	23.06	22.80	21.80	23.08	22.32	22.58	47.76	8.13
4s	20.70	20.14	22.00	20.76	20.26	21.72	23.06	22.80	21.80	23.08	22.32	22.58	8.55	2.46
4t	22.60	21.12	24.00	21.94	21.20	20.06	23.06	22.80	21.80	23.08	22.32	22.58	4.01	5.97
4u	22.80	21.46	14.50	16.66	21.38	21.68	23.06	22.80	21.80	23.08	22.32	22.58	14.59	18.47
4v	17.18	17.42	18.06	17.06	18.20	17.64	18.86	18.30	18.38	19.66	19.84	18.32	8.18	1.96

4w	20.60	19.08	20.16	19.06	18.94	18.44	18.86	18.30	18.38	19.66	19.84	18.32	-3.06	3.83
5a	20.08	19.22	20.82	21.28	19.42	17.74	23.06	22.80	21.80	23.08	22.32	22.58	14.52	6.32
5b	19.62	18.40	18.06	17.78	18.90	17.98	23.06	22.80	21.80	23.08	22.32	22.58	21.17	2.78
5c	23.06	23.72	21.80	22.42	23.76	21.64	23.06	22.80	21.80	23.08	22.32	22.58	-0.65	3.27
5d	21.84	20.70	20.60	22.18	22.16	20.50	23.06	22.80	21.80	23.08	22.32	22.58	6.51	0.31
5e	18.00	18.00	18.24	17.34	18.10	17.86	23.06	22.80	21.80	23.08	22.32	22.58	23.89	0.59

Diameter of mushroom cake (3mm).

Table S3. The primary data for table 1(*F. graminearum*)

F. graminearum	The value of the diameter of the plaque (mm)												The average inhibition rate (%)	SD
	Group 1		Group 2		Group 3		Blank 1		Blank 2		Blank 3			
Compound	x	y	x	y	x	y	x	y	x	y	x	y		
4a	17.56	17.90	20.10	18.16	16.30	16.74	20.40	19.46	20.96	20.94	23.10	22.80	19.06	7.15
4b	17.20	16.40	18.54	18.10	15.62	15.76	20.40	19.46	20.96	20.94	23.10	22.80	23.75	7.22
4c	15.64	13.52	15.30	16.60	15.72	14.18	20.40	19.46	20.96	20.94	23.10	22.80	33.47	3.88
4d	14.26	14.14	15.00	12.86	13.40	13.90	20.40	19.46	20.96	20.94	23.10	22.80	40.22	1.50
4e	15.10	18.00	14.82	15.00	15.20	16.56	20.40	19.46	20.96	20.94	23.10	22.80	30.07	4.51
4f	0.00	0.00	0.00	0.00	0.00	0.00	23.66	20.90	20.30	20.54	21.36	19.58	100	0.00
4g	-	-	9.26	10.60	10.62	12.68	23.66	20.90	20.30	20.54	21.36	19.58	56.86	6.74
4h	15.00	13.84	17.16	17.92	16.00	15.96	20.40	19.46	20.96	20.94	23.10	22.80	28.98	8.54
4i	19.18	19.14	-	-	19.60	19.44	23.66	20.90	20.30	20.54	21.36	19.58	9.51	1.41
4j	17.14	21.36	21.84	21.96	19.94	22.66	20.40	19.46	20.96	20.94	23.10	22.80	2.52	7.60
4k	18.92	16.78	17.56	16.48	17.52	16.64	20.40	19.46	20.96	20.94	23.10	22.80	21.67	2.53

4l	16.80	16.40	16.14	16.14	16.44	15.50	20.40	19.46	20.96	20.94	23.10	22.80	27.58	1.78
4m	19.94	20.62	18.90	17.36	17.72	18.48	20.40	19.46	20.96	20.94	23.10	22.80	13.35	6.84
4n	20.00	20.60	16.84	17.66	19.10	16.12	20.40	19.46	20.96	20.94	23.10	22.80	15.81	9.12
4o	10.36	11.00	11.20	11.14	12.54	11.00	20.40	19.46	20.96	20.94	23.10	22.80	55.10	2.99
4p	20.50	20.40	19.00	18.70	20.10	19.46	20.40	19.46	20.96	20.94	23.10	22.80	8.66	4.40
4q	12.70	13.42	13.30	12.36	14.20	14.00	20.40	19.46	20.96	20.94	23.10	22.80	43.48	3.70
4s	21.14	20.24	20.76	19.36	21.00	20.56	20.40	19.46	20.96	20.94	23.10	22.80	4.19	2.15
4t	15.72	17.36	15.50	16.00	16.48	15.60	20.40	19.46	20.96	20.94	23.10	22.80	28.27	2.19
4u	22.40	21.24	21.36	22.54	23.00	21.92	20.40	19.46	20.96	20.94	23.10	22.80	-4.38	1.85
4v	16.10	17.44	14.52	14.42	19.18	16.44	23.66	20.90	20.30	20.54	21.36	19.58	26.07	9.47
4w	23.72	21.56	21.48	19.24	16.54	15.62	23.66	20.90	20.30	20.54	21.36	19.58	7.55	18.44
5a	18.72	16.76	21.90	19.00	18.80	18.52	20.40	19.46	20.96	20.94	23.10	22.80	12.73	7.54
5b	15.28	14.76	14.76	14.10	14.66	14.52	20.40	19.46	20.96	20.94	23.10	22.80	36.09	1.67
5c	19.20	18.36	18.24	18.30	20.76	22.86	20.40	19.46	20.96	20.94	23.10	22.80	9.06	10.47
5d	16.90	15.90	17.80	18.00	20.20	18.00	20.40	19.46	20.96	20.94	23.10	22.80	19.02	7.40
5e	16.70	16.90	16.68	15.90	15.20	14.76	20.40	19.46	20.96	20.94	23.10	22.80	28.74	5.14

Diameter of mushroom cake (3mm).

Table S4. The primary data for table 1(*E. turcicum*)

E. turcicum	The value of the diameter of the plaque (mm)												The average inhibition rate (%)	SD
	Group 1		Group 2		Group 3		Blank 1		Blank 2		Blank 3			
Compound	x	y	x	y	x	y	x	y	x	y	x	y		

4a	17.46	16.34	17.88	17.76	17.50	17.54	20.20	20.36	21.06	20.34	21.00	19.92	17.54	2.68
4b	10.00	9.58	9.64	9.80	11.00	9.52	20.20	20.36	21.06	20.34	21.00	19.92	60.39	1.68
4c	15.00	14.70	15.74	15.30	15.48	15.12	20.20	20.36	21.06	20.34	21.00	19.92	30.07	1.95
4d	12.12	12.56	12.34	12.80	12.24	12.46	20.20	20.36	21.06	20.34	21.00	19.92	46.11	0.74
4e	15.56	15.70	16.48	16.46	15.44	15.48	20.20	20.36	21.06	20.34	21.00	19.92	26.47	3.09
4f	5.84	5.56	5.48	5.64	5.12	5.00	19.36	19.54	20.50	20.00	20.90	20.16	85.71	1.97
4g	12.70	14.50	13.50	13.06	13.36	13.72	19.36	19.54	20.50	20.00	20.90	20.16	38.67	1.00
4h	20.18	19.62	18.20	17.26	18.38	18.00	20.20	20.36	21.06	20.34	21.00	19.92	10.72	6.54
4i	20.34	20.52	18.90	18.72	22.20	20.08	19.36	19.54	20.50	20.00	20.90	20.16	-0.29	6.99
4j	18.92	18.60	19.06	19.18	-	-	20.20	20.36	21.06	20.34	21.00	19.92	8.81	1.46
4k	19.28	19.30	19.38	18.32	19.00	19.00	20.20	20.36	21.06	20.34	21.00	19.92	8.20	1.28
4l	15.80	15.24	17.00	16.58	16.54	16.38	20.20	20.36	21.06	20.34	21.00	19.92	24.16	3.77
4m	17.76	17.62	18.00	17.92	18.04	17.64	20.20	20.36	21.06	20.34	21.00	19.92	15.16	0.77
4n	15.18	14.86	15.34	15.44	15.52	15.30	20.20	20.36	21.06	20.34	21.00	19.92	29.79	1.26
4o	15.10	15.64	15.68	15.20	16.26	15.54	20.20	20.36	21.06	20.34	21.00	19.92	28.09	1.65
4p	18.40	17.88	18.86	18.38	18.84	18.72	20.20	20.36	21.06	20.34	21.00	19.92	11.25	1.91
4q	12.86	12.66	13.20	12.94	12.86	12.70	20.20	20.36	21.06	20.34	21.00	19.92	43.54	0.99
4s	19.52	20.00	19.06	18.84	19.48	19.62	20.20	20.36	21.06	20.34	21.00	19.92	6.06	2.40
4t	20.54	19.28	19.78	20.76	20.70	20.72	20.20	20.36	21.06	20.34	21.00	19.92	1.05	2.29
4u	18.76	18.54	19.88	19.52	19.12	18.50	20.20	20.36	21.06	20.34	21.00	19.92	8.16	3.24
4v	16.82	17.60	16.92	17.04	18.10	18.14	19.36	19.54	20.50	20.00	20.90	20.16	15.46	3.53
4w	19.46	18.88	19.32	18.74	-	-	19.36	19.54	20.50	20.00	20.90	20.16	5.72	0.58
5a	15.90	16.14	17.62	16.46	17.40	17.18	20.20	20.36	21.06	20.34	21.00	19.92	21.15	3.85

5b	14.62	14.68	15.00	14.92	15.12	15.18	20.20	20.36	21.06	20.34	21.00	19.92	31.81	1.44
5c	18.90	19.78	17.66	18.00	19.08	19.20	20.20	20.36	21.06	20.34	21.00	19.92	9.78	4.69
5d	16.92	16.66	17.50	17.68	16.72	17.24	20.20	20.36	21.06	20.34	21.00	19.92	19.22	2.39
5e	14.86	15.42	15.82	15.68	14.82	15.42	20.20	20.36	21.06	20.34	21.00	19.92	29.42	2.05

Diameter of mushroom cake (3mm).

Table S5. The primary data for table 1(*B. cinerea*)

B. cinerea	The value of the diameter of the plaque (mm)												The average inhibition rate (%)	SD
	Group 1		Group 2		Group 3		Blank 1		Blank 2		Blank 3			
Compound	x	y	x	y	x	y	x	y	x	y	x	y		
4a	20	20	20.04	18.28	20.08	20.08	20.62	23.9	18.56	15.38	20.48	21.54	2.07	3.17
4b	16.12	17.78	20.09	18.86	23.12	20.08	20.62	23.9	18.56	15.38	20.48	21.54	4.59	14.48
4c	20.26	17.14	20.16	20	22.1	19.4	20.62	23.9	18.56	15.38	20.48	21.54	2.28	6.45
4d	13.88	13.34	13.6	12.38	15.52	16.28	20.62	23.9	18.56	15.38	20.48	21.54	36.77	9.53
4e	14.04	17.6	14.32	15.62	19.78	20.2	20.62	23.9	18.56	15.38	20.48	21.54	19.61	16.71
4f	14	11.98	14.52	13.08	14.76	14.66	20.62	23.9	18.56	15.38	20.48	21.54	38.85	5.35
4g	21.18	19.78	20.96	19.42	20.82	19.76	20.62	23.9	18.56	15.38	20.48	21.54	-1.49	0.92
4h	21.58	22.24	23.18	20	23	19.4	20.62	23.9	18.56	15.38	20.48	21.54	-9.25	2.21
4i	16.76	12.7	20.08	20.08	20.08	20.08	20.62	23.9	18.56	15.38	20.48	21.54	11.09	19.21
4j	24.82	22	25	21	20.08	20.08	20.62	23.9	18.56	15.38	20.48	21.54	-12.96	11.29
4k	23.96	21.24	19	16.7	19.52	20.36	20.62	23.9	18.56	15.38	20.48	21.54	-0.31	14.81
4l	20	20.26	18.28	15.46	18.26	16.28	20.62	23.9	18.56	15.38	20.48	21.54	12.38	11.06
4n	18.32	19.48	22.8	21.18	18.84	17.44	20.62	23.9	18.56	15.38	20.48	21.54	2.51	12.68
4o	16.04	16.04	17.26	16.88	18.88	20	20.62	23.9	18.56	15.38	20.48	21.54	15.94	10.84
4p	21.14	19.28	25.2	21.22	23.38	20.3	20.62	23.9	18.56	15.38	20.48	21.54	-10.41	9.34
4q	15.88	16.92	11.38	13.86	15.76	15	20.62	23.9	18.56	15.38	20.48	21.54	32.84	12.16

4s	20.5	19.37	17.16	19.94	20	16.58	20.62	23.9	18.56	15.38	20.48	21.54	7.18	5.50
4t	17.68	12.78	17.28	18.72	19.7	19.22	20.62	23.9	18.56	15.38	20.48	21.54	15.65	13.36
4u	15.36	14.6	10.22	13.54	23.28	22.06	20.62	23.9	18.56	15.38	20.48	21.54	22.20	34.55
4v	17.08	15.86	19.4	16.36	19.6	17.6	20.62	23.9	18.56	15.38	20.48	21.54	15.11	6.74
4w	21.38	20.14	22.1	17	20.08	20.08	20.62	23.9	18.56	15.38	20.48	21.54	-0.31	3.77
5a	20.14	19.28	19.16	18.04	20.32	20.34	20.62	23.9	18.56	15.38	20.48	21.54	3.32	5.45
5b	10.12	18.02	15.08	16.1	15.48	16.58	20.62	23.9	18.56	15.38	20.48	21.54	30.16	6.40
5c	22	22.56	23.56	24.86	20.08	20.08	20.62	23.9	18.56	15.38	20.48	21.54	-13.12	12.85
5d	18.2	15.88	15.34	15.34	16.76	18.02	20.62	23.9	18.56	15.38	20.48	21.54	21.70	6.82
5e	12.84	17.04	16.36	13.96	15.48	15.32	20.62	23.9	18.56	15.38	20.48	21.54	30.56	1.43

Diameter of mushroom cake (4mm).

Table S6. The primary data for table 1(*C. capsica*)

C. capsica	The value of the diameter of the plaque (mm)												The average inhibition rate (%)	SD
	Group 1		Group 2		Group 3		Blank 1		Blank 2		Blank 3			
Compound	x	y	x	y	x	y	x	y	x	y	x	y		
4b	28.86	28.4	31.18	29	32.86	32.44	28.76	28.98	30.24	32.32	30.76	31.46	-0.14	7.70
4c	29.38	29.64	30.34	29.88	31.28	28.78	28.76	28.98	30.24	32.32	30.76	31.46	2.03	1.23
4d	23.24	24.46	24.86	23.86	25.08	25.7	28.76	28.98	30.24	32.32	30.76	31.46	22.28	2.97
4e	25.72	24.22	26.78	26	25.24	24.7	28.76	28.98	30.24	32.32	30.76	31.46	18.84	3.10
4f	6.74	6.4	7.56	7.78	8.8	7.56	28.76	28.98	30.24	32.32	30.76	31.46	86.85	3.11
4g	17.58	18.26	18.66	16.38	18.22	17.88	28.76	28.98	30.24	32.32	30.76	31.46	47.65	1.05
4h	31.88	29.46	30.56	31.5	33.1	31.22	28.76	28.98	30.24	32.32	30.76	31.46	-3.28	2.94
4j	31.62	31.76	33.68	33.06	31.02	31.6	28.76	28.98	30.24	32.32	30.76	31.46	-6.45	4.15
4k	30.02	28.54	32.62	32.18	31.86	32.54	28.76	28.98	30.24	32.32	30.76	31.46	-3.31	6.61

4l	33.04	31.78	30.36	30.58	31.18	30.84	28.76	28.98	30.24	32.32	30.76	31.46	-3.32	3.79
4n	29.48	30.02	30.54	29.16	29.6	27.38	28.76	28.98	30.24	32.32	30.76	31.46	4.00	2.87
4o	24.54	25.14	24.92	25.28	25.42	24.22	28.76	28.98	30.24	32.32	30.76	31.46	20.82	0.59
4p	30.16	31.22	30.36	30.96	34	32.86	28.76	28.98	30.24	32.32	30.76	31.46	-4.44	6.02
4q	16.34	15.96	15.84	15.38	15.78	15.18	28.76	28.98	30.24	32.32	30.76	31.46	55.54	1.34
4s	32.16	32.16	32.34	30.92	30.32	28.44	28.76	28.98	30.24	32.32	30.76	31.46	-2.41	5.59
4u	29.12	29.24	31.38	31.82	32.74	33.14	28.76	28.98	30.24	32.32	30.76	31.46	-3.10	7.21
4v	30	28.36	27.12	26.24	27.5	36.96	28.76	28.98	30.24	32.32	30.76	31.46	4.00	10.52
4w	28.38	27.02	32.24	30.72	30	28.58	28.76	28.98	30.24	32.32	30.76	31.46	3.52	7.18
5b	25.1	25.86	24.52	22.72	25.66	25.98	28.76	28.98	30.24	32.32	30.76	31.46	20.62	4.48
5e	27.88	28.66	28	31.38	27.58	29.58	28.76	28.98	30.24	32.32	30.76	31.46	5.96	2.83

Diameter of mushroom cake (4mm).

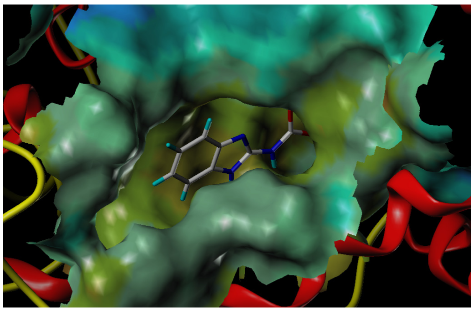

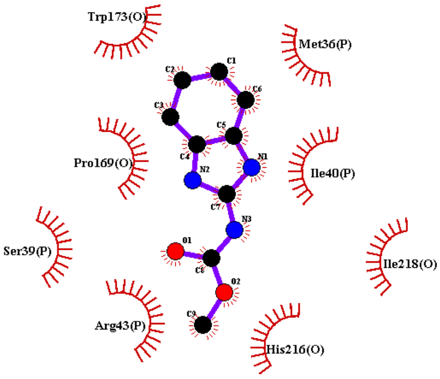

A B

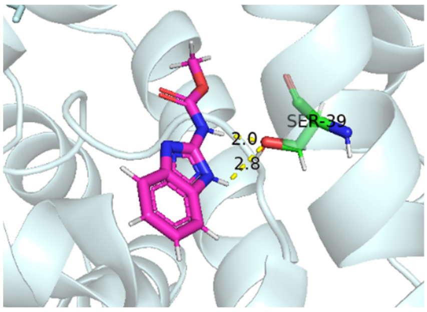

C

Figure S5. 3D docking of **carbendazim** with the active pocket of protein 2fbw (A). 2D ligand hydrophobic interaction (B) of **carbendazim** with amino acid residues. Hydrogen bond interaction of **carbendazim** with amino acid residues (C).
